# TRMT6‐Mediated m^1^A Modification of *CDK9* mRNA Is a Dual‐Pronged Pathogenic Driver for HBV‐Related Hepatocellular Carcinoma

**DOI:** 10.1002/advs.202514172

**Published:** 2026-04-20

**Authors:** Rui Zhang, Dandan Zong, Rui Liu, Yubo Wang, Qingqing Gu, Yao Yao, Wenfang Zheng, Mengmeng Yuan, Simeng Wang, Rongrong Cui, Daxu Li, Siwen Dang, Peng Hou

**Affiliations:** ^1^ Department of Endocrinology and Metabolism The First Affiliated Hospital of Xi'an Jiaotong University Xi'an P. R. China; ^2^ International Joint Research Center for Tumor Precision Medicine of Shaanxi Province The First Affiliated Hospital of Xi'an Jiaotong University Xi'an P. R. China; ^3^ Department of Infectious Diseases Tangdu Hospital The Fourth Military Medical University Xi'an P.R. China; ^4^ Yulin Hospital The First Affiliated Hospital of Xi'an Jiaotong University Yulin P. R. China; ^5^ Department of Radio‐Oncology The First Affiliated Hospital of Xi'an Jiaotong University Xi'an P. R. China; ^6^ Department of Stomatology The First Affiliated Hospital of Xi'an Jiaotong University Xi'an P. R. China; ^7^ Department of Endocrinology and Metabolism The Second Affiliated Hospital Xi'an Jiaotong University Xi'an P. R. China

**Keywords:** CDK9, Hepatocellular carcinoma (HCC), N^1^‐methyladenosine (m^1^A), TARDBP, TRMT6

## Abstract

Hepatocellular carcinoma (HCC) is a leading cause of death worldwide, with hepatitis B virus (HBV) infection being the major risk factor. Dysregulation of mRNA methylation contributes to tumorigenesis and virus replication. However, the association of N^1^‐methyladenosine (m^1^A) modification with HCC progression and HBV replication remains unclear. Here, single‐nucleus RNA sequencing (snRNA‐seq) of 4 HCC and 7 adjacent tissues (2 from this study and 5 from the GSE242889) revealed elevated mRNA methylation in HCCs, with increased expression of m^1^A “writers” and “readers” and decreased expression of m^1^A “erasers”. Among them, m^1^A writer *TRMT6* was up‐regulated in HCC and correlated with poor patient prognosis. TRMT6 knockdown strikingly restrained the malignant phenotypes and tumorigenicity of HCC cells as well as HBV replication. Mechanistically, TRMT6‐mediated m^1^A modification enhanced the stability and translation efficiency of cyclin‐dependent kinase 9 (*CDK9*) mRNA. Elevated CDK9 facilitated HCC progression by up‐regulating its downstream oncogenic effectors, and stimulated HBV replication via TARDBP phosphorylation at Ser254 to enhance pgRNA transcription and repress pgRNA splicing. CDK9 inhibitor FIT‐039 abrogated these effects without obvious toxicity. Thus, TRMT6‐mediated m^1^A modification dually drives HCC malignancy and HBV replication, representing a promising therapeutic target, and CDK9 inhibition may constitute an effective strategy for HBV‐related HCC.

## Graphical abstract

TRMT6‐mediated m^1^A modification in *CDK9* mRNA enhances its mRNA stability and translation efficiency, thereby increasing the protein levels of CDK9. Upregulated CDK9 promotes the progression of HCC by elevating the levels of oncogenic factors including p‐STAT3, MCL1, and BCL‐2. On the other hand, CDK9 phosphorylates TARDBP at Ser254 to activate HBV core promoter and inhibit the pgRNA splicing, ultimately driving HBV replication.

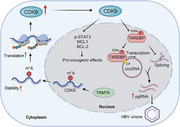

## 1 Introduction

Hepatocellular carcinoma (HCC) is a prevalent malignancy and a cause of death globally, which accounts for 85%–90% of all liver cancer cases [1]. As reported in 2020, the incidence and mortality of HCC rank sixth and third globally [2]. In China, the incidence of HCC ranks fourth in the total and is also the second leading cause of cancer‐related deaths [3], with a 5‐year relative survival rate is 14.4% [4]. HCC is highly aggressive and has a high mortality, and there is a lack of effective treatment. Thus, it is urgent to elucidate its pathogenesis and identify new therapeutic targets to improve the prognosis of patients.

Hepatitis B virus (HBV) infection is a main pathogenic factor of HCC [5], increasing the risk of developing HCC by 10‐ to 30‐fold in affected populations relative to uninfected individuals [6]. HBV contains a 3.2‐kb partially double‐stranded relaxed circular DNA (rcDNA) genome, which is repaired into covalently closed circular DNA (cccDNA) in the nucleus [7]. cccDNA encodes six overlapped transcripts, and a 3.5‐kb transcript termed pregenomic RNA (pgRNA) can be reversely transcribed to replenish the cccDNA pool [8]. High levels of HBV DNA promote the initiation and progression of HCC via HBV DNA integration [9], HBV‐encoded oncoproteins [10], increased oxidative stress [11] and metabolic reprogramming [12]. Thus, effective antiviral therapies can improve the prognosis of HBV‐related HCC patients [13].

Dysregulation of mRNA modifications has been implicated in tumorigenesis, and may serve as an effective target for cancer therapy, such as mRNA methylation [14]. Despite the extensively studied role of N^6^‐methyladenosine (m^6^A) modification in various types of cancer, emerging evidence suggests that other forms of RNA methylation, such as N^1^‐methyladenosine (m^1^A) may also contribute to tumor progression. However, the functional significance of m^1^A, particularly mRNA m^1^A modification, remains poorly defined in human malignancies such as HCC. m^1^A, also known as methylated adenosine at N^1^ position, is one of dynamic chemical modifications in eukaryotic tRNA, mRNA, rRNA, and lncRNA [15]. m^1^A is typically modified in “GUUCRA” motif and is a reversible process regulated by m^1^A “writers”, “erasers”, and “readers” proteins [16]. Methyltransferases, also called “writers”, including TRMT6, TRMT61A, TRMT61B, and TRMT10C, are responsible for installing methyl groups to the N^1^ site of adenosine [17], while the “erasers” such as FTO, ALKBH1, and ALKBH3 catalyze the removal of m^1^A [18]. For m^1^A modification exerting its biological effects, it needs to be recognized by RNA‐binding proteins called “readers”, including YTHDF1‐3 and YTHDC1 [19]. As a m^1^A “writer” protein, TRMT6 typically forms a tetramer complex with TRMT61A to regulate tRNA m^1^A modification, thereby affecting various biological process including tumorigenesis and progression [20, 21]. In addition, TRMT6 has also been involved in m^1^A modification of mRNA [16, 22]. However, whether TRMT6‐mediated mRNA m^1^A modification is related to HCC progression and HBV replication remains unclear.

In this study, we perform single nucleus RNA sequencing (snRNA‐seq) in HCC tissues and adjacent controls, and discover high levels of m^1^A modification and TRMT6 in HCCs. Additionally, increased expression of TRMT6 not only predicts poor patient survival, but also promotes the malignant phenotypes of HCC cells and HBV replication. Further mechanistic studies reveal that TRMT6‐mediated m^1^A modification of *CDK9* mRNA enhances its stability and translation efficiency. As a result, CDK9 is up‐regulated in HCC. On one hand, CDK9 exerts its oncogenic functions by activating its downstream oncogenic effectors. On the other hand, CDK9 promotes HBV replication by phosphorylating TARDBP at Ser254. Thus, this study highlights the dual role of TRMT6‐mediated m^1^A modification of *CDK9* mRNA in HCC progression and HBV replication, and suggests that targeting TRMT6‐mediated up‐regulation of CDK9 may be a promising therapeutic strategy for HCC, especially HBV‐related HCC.

## 2 Results

### 2.1 High Levels of m^1^A Modification and TRMT6 in HCC Tissues by Single‐Nucleus Transcriptome Profiles

**FIGURE 1:**
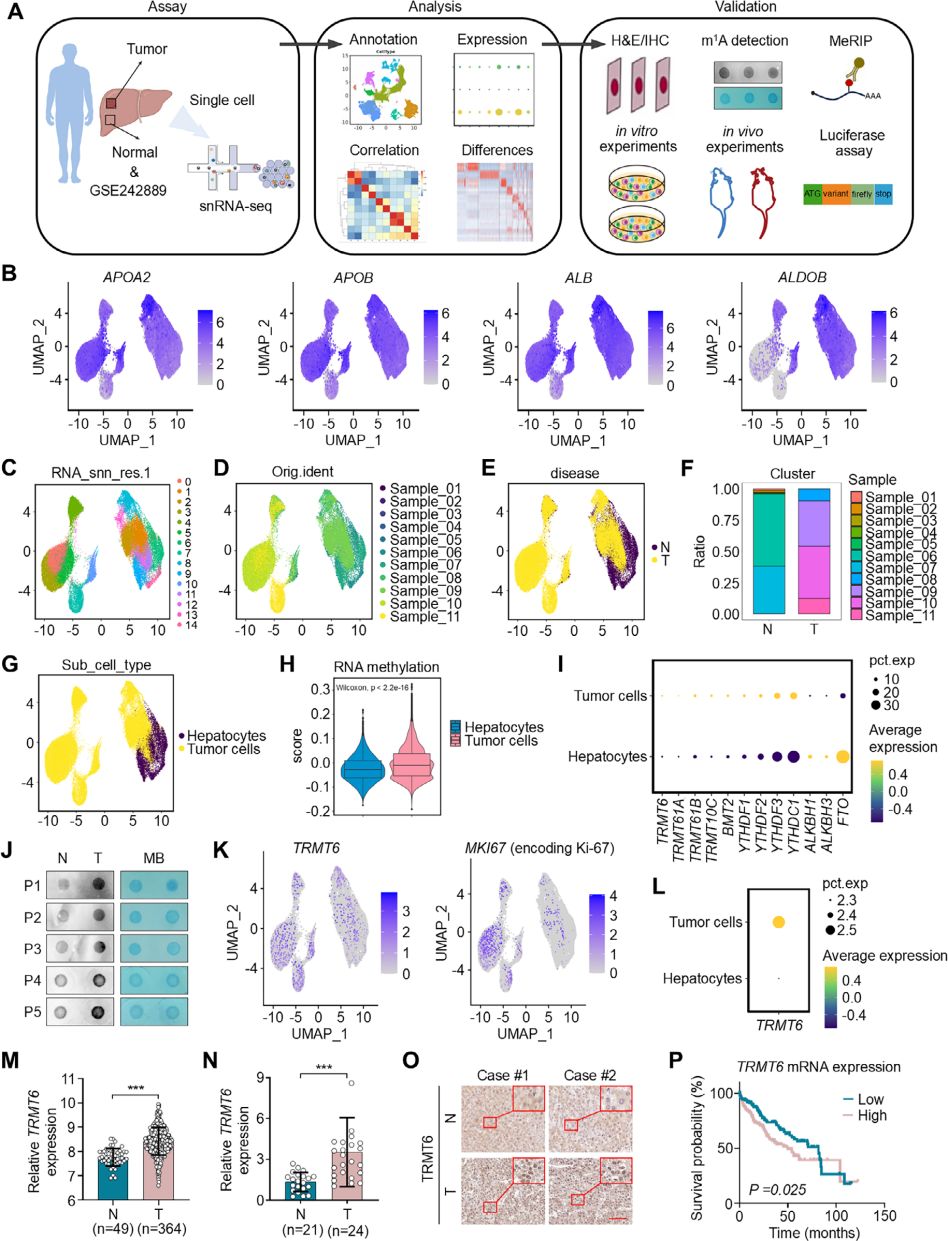
Cell type identification by snRNA‐seq analysis of human HCC and non‐cancerous tissues. (A) Flowchart overview of single‐nucleus RNA‐seq of human HCC and control tissues. (B,C) Hepatocytes in different tissues were identified by UMAP plots. (D,E) Data integration was conducted using harmony to remove batch effects. (F,G) Cells were defined as hepatocytes and tumor cells according to the specific patterns of gene expression in hepatocytes and tumor cells and cell‐derived tissues. (H) The gene set scores of RNA methylation‐related molecules in hepatocytes and tumor cells. (I) Gene expression of m^1^A‐related molecules in hepatocytes and tumor cells. (J) The representative images of m^1^A levels in 5 pairs of HCC tissues (T) and their matched non‐cancerous tissues (N) were determined by dot‐blot assays, and methylene blue staining (MB) exhibited the loading control. (K‐L) *TRMT6* expression in hepatocytes and tumor cells. (M) The mRNA expression of *TRMT6* was analyzed using HCC dataset (T, tumor tissues; N, non‐cancerous liver tissues) in the TCGA database. (N) The mRNA expression of *TRMT6* was analyzed in HCC tissues (T) and non‐cancerous liver tissues (N). *β‐actin* was used as a reference gene. (O) The representative images of paraffin sections from HCC tissues and control tissues using IHC staining, Scale bar, 100 µm. (P) Survival curves of HCC patients with high and low expression of *TRMT6*. The data were shown as the mean ± SD. ***, *P* <0.001.

We performed snRNA‐seq on four HBV‑positive HCC tissues and two adjacent non‑cancerous tissues, and integrated an additional five adjacent non‑cancerous tissues from the GSE242889 dataset for comprehensive analysis (Figure 1A). A total of 76634 cells were included in the subsequent study, with duplicate cells and cells of poor quality removed. We visualized human liver cell populations by using uniform manifold approximation and projection (UMAP) (Figure S1A–D, Supporting Information). Based on the expression matrix of their specific markers, we identified nine major cell types, including fibroblasts (FC), hepatocytes (HEP), endothelial cells (EN), doubled cells (Doubled), proliferating cells (PC), myeloid cells (MC), epithelial cells (EPI), B cells (BC) and T/NK cells (T_NK) (Figure S1E). The ratios of all these cell types in tumor and control tissues are shown in Figure S1F.

We next extracted 40674 hepatocytes for further analysis. After data dimensionality reduction, we visualized human liver cell populations by using UMAP, and the cells were further classified into 15 clusters (Figure 1B,C). Data integration was conducted using harmony to remove batch effects and ensure consistency and comparability (Figure 1D,E). According to the expression of specific makers, cells were defined as hepatocytes and tumor cells (Figure 1F,G). Then, we investigated the scores of RNA methylation‐related gene set in hepatocytes and tumor cells, and found that RNA methylation levels were higher in the latter than the former (Figure 1H; Figure S2A). To explore the role of m^1^A modification in HCC, we analyzed gene expression of m^1^A‐related molecules, and found that the expression of m^1^A writers and readers was higher in tumor cells than hepatocytes, while the expression of its erasers was the opposite, which was lower in tumor cells than in hepatocytes (Figure 1I; Figure S2B). These results suggest that m^1^A modification is linked to HCC tumorigenesis or progression.

To verify the above results, we detected m^1^A levels in 5 paired of HCC and control tissues by dot‐blot assay, and found a significant increase of m^1^A levels in HCC tissues (Figure 1J; Figure S2C). As a major m^1^A writer, *TRMT6* is highly expressed in tumor cells, which was consistent with the expression pattern of proliferation marker *MKI67* (Figure 1K,L; Figure S2D). Next, we analyzed mRNA levels of *TRMT6* using The Cancer Genome Atlas (TCGA) database. Both unpaired (Figure 1M) and paired (Figure S2E) analysis consistently showed a significant increase in *TRMT6* expression in HCCs compared with control subjects. We further evaluated the mRNA and protein levels of TRMT6 in fresh frozen HCC tissues and paraffin‐embedded HCC sections as well as their control subjects. The results of qRT‐PCR (Figure 1N) and immunohistochemistry (IHC) (Figure 1O; Figure S2F) further supported the above conclusion. Kaplan‐Meier survival analysis also indicated that higher *TRMT6* expression was strongly related to poor survival of HCC patients (Figure 1P). These data, taken together, suggest that TRMT6‐mediated m^1^A modification may play an oncogenic role in HCC.

### 2.2 TRMT6‐Mediated m^1^A Modification Promotes the Malignant Phenotypes of HCC Cells

**FIGURE 2:**
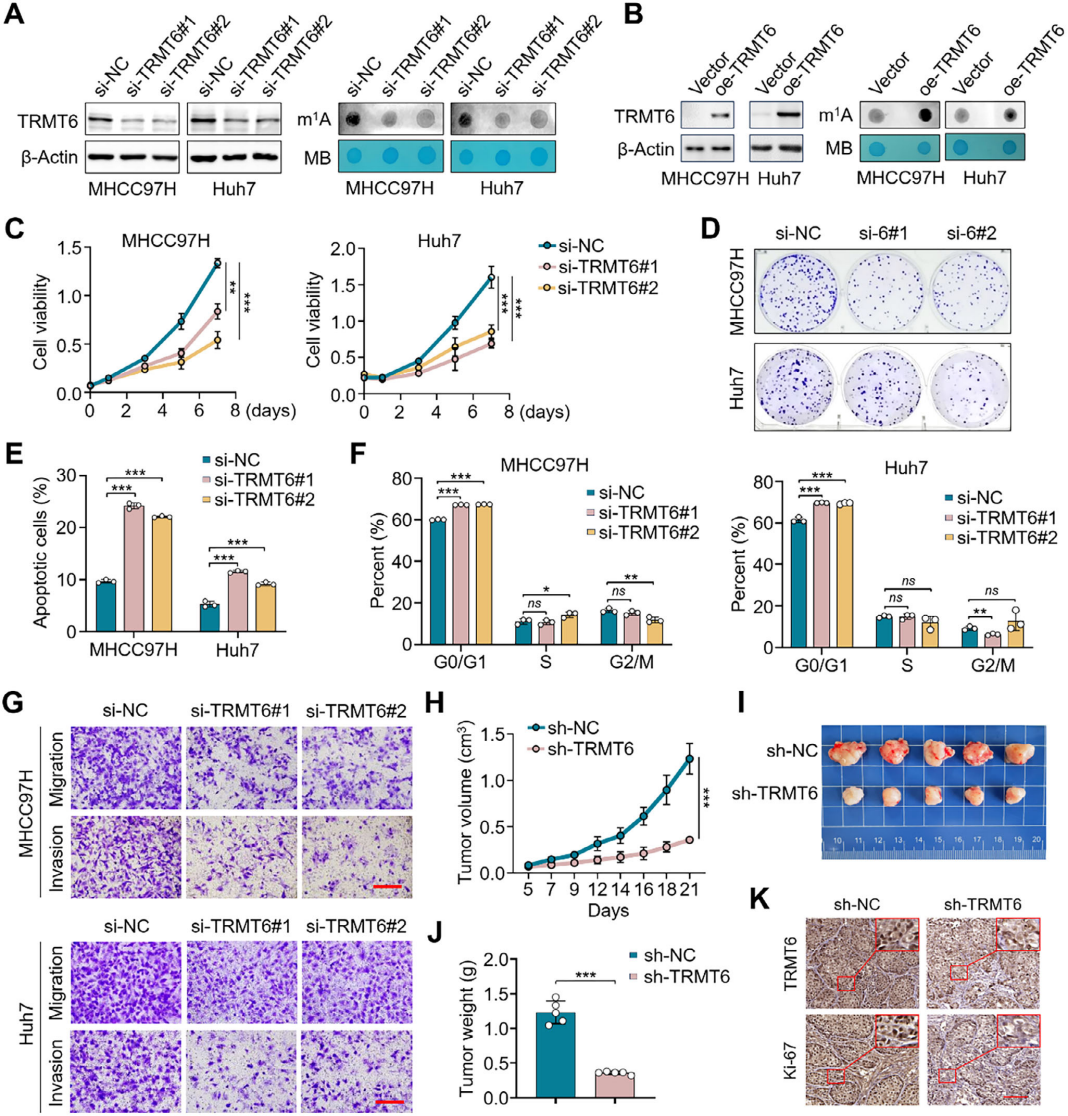
TRMT6‐mediated m^1^A modification enhances the malignant behaviors of HCC cells. Knockdown (A) and overexpression (B) of TRMT6 in MHCC97H and Huh7 cells by siRNAs (si‐TRMT6#1 and #2) was validated by western blotting analysis in left panel. β‐Actin was used as a loading control. The m^1^A levels were measured in TRMT6‐knockdown and ‐overexpressing cells by dot ‐ blot assay in right panels, and methylene blue staining (MB) exhibited the loading control. (C) TRMT6 was knocked down in MHCC97H and Huh7 cells, and its effect on cell viability was evaluated by MTT assay. (D) The effect of TRMT6 knockdown on colony formation ability of MHCC97H and Huh7 cells. Shown were representative images of colony formation. (E) The effect of TRMT6 knockdown on the apoptosis and cell cycle (F) of MHCC97H and Huh7 cells was assessed by flow cytometry. (G) The effect of TRMT6 knockdown on the migration and invasion of MHCC97H and Huh7 cells was evaluated by transwell assays. Shown were representative images of cell migration/invasion. Scale bar, 200 µm. Each assay was performed with three biological replicates. (H) Tumor growth curves comparing nude mice subcutaneously injected with TRMT6‐knockdown MHCC97H cells versus control cells (n = 5/group). (I) The images of dissected tumors and tumor weights (J) of the indicated groups. (K) IHC staining of TRMT6 and Ki‐67 in the indicated tumors. The representative images were shown in the panel. Scale bar, 100 µm. Each experiment was done with three biological replicates. The data were shown as the mean ± SD. *, *P* < 0.05; **, *P* < 0.01; ***, *P* < 0.001; *ns*, no significance.

To investigate the effect of TRMT6‐mediated m^1^A on HCC, we initially knocked down and ectopically expressed TRMT6 in MHCC97H and Huh7 cells (Figure 2A,B, left panel). The low levels of m^1^A in TRMT6‐knockdown cells (Figure 2A, right panel) and high levels of m^1^A in TRMT6‐overexpression cells (Figure 2B, right panel) further demonstrated the regulatory role of TRMT6 in m^1^A modification. Next, we evaluated the impact of TRMT6 on cell viability. The results discovered that TRMT6 knockdown substantially impaired the proliferation (Figure 2C) and colony formation capacity (Figure 2D; Figure S3A) of HCC cells. Furthermore, flow cytometry analysis revealed that apoptotic cells were significantly elevated upon TRMT6 knockdown (Figure 2E; Figure S3B), and TRMT6 knockdown markedly triggered G0/G1 cell cycle arrest (Figure 2F; Figure S3C). Besides, transwell assays indicated that TRMT6 knockdown distinctly suppressed the migration and invasion capability of HCC cells (Figure 2G; Figure S3D). Conversely, TRMT6 overexpression strikingly enhanced their proliferation (Figure S3E) and colony formation (Figure S3F) abilities.

To further evaluated the impact of TRMT6‐mediated m^1^A modification on the tumorigenicity of HCC cells in vivo, we employed a lentivirus system to stably knocked down TRMT6 in MHCC97H cells, and subcutaneously inoculated TRMT6‐knockdown cells and their control cells into nude mice to construct xenograft tumor models. The results exhibited that TRMT6‐knockdown cell‐derived xenograft tumors grew slower (Figure 2H) and weighted less (Figure 2I,J) compared with control tumors. Additionally, the decreased proliferative capacity of TRMT6‐knockdown cells was confirmed by Ki‐67 staining in the xenograft tumors (Figure 2K; Figure S3G). Collectively, these data support an oncogenic role of TRMT6‐mediated m^1^A modification in HCC.

### 2.3 TRMT6‐Mediated m ^1^A Modification Facilitates HBV Replication

**FIGURE 3:**
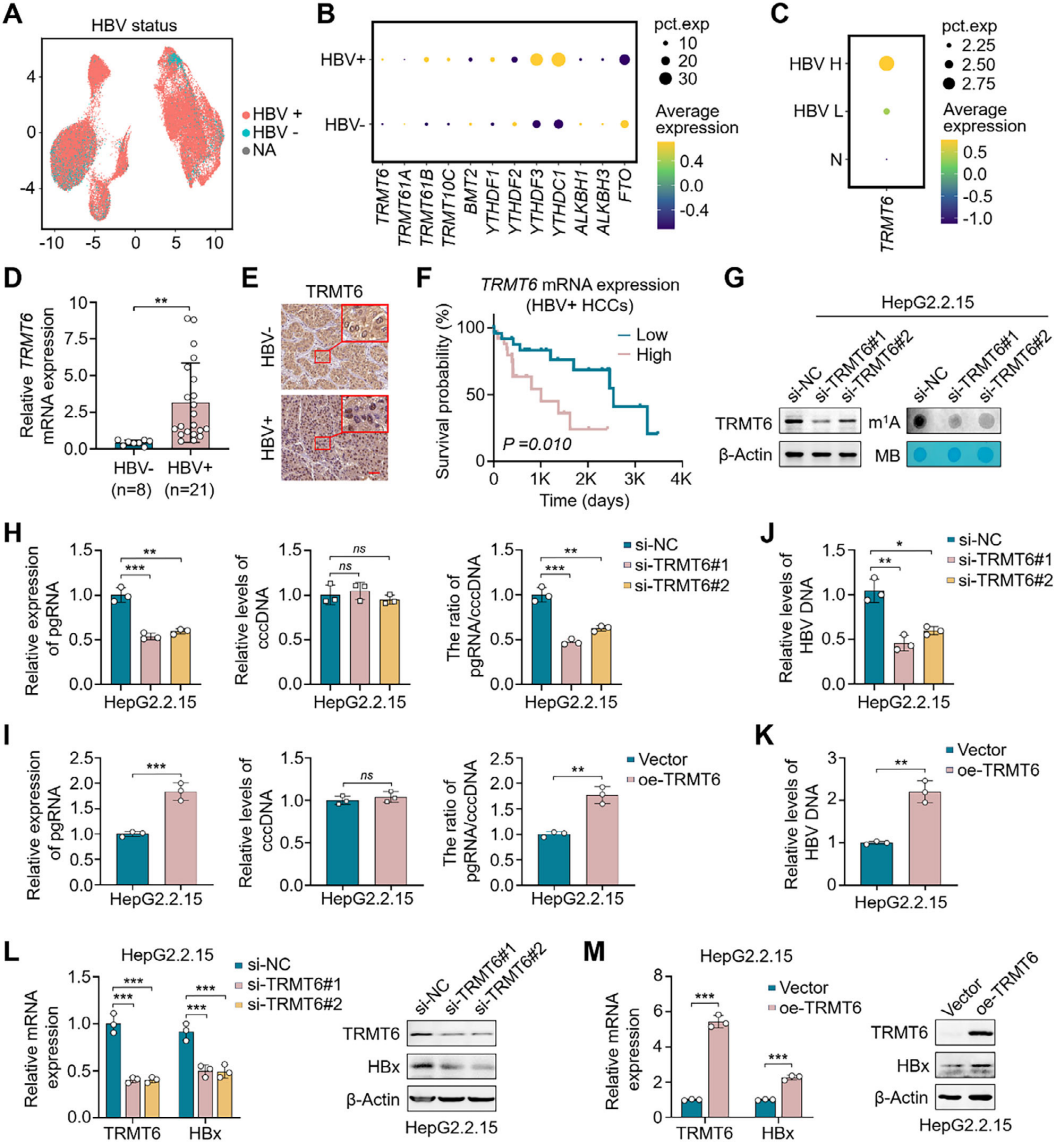
The promoting effect of TRMT6‐mediated m^1^A modification on HBV replication. (A) UMAP showing a universal HBV infection in HCC patients. The HBV infected cells were depicted in red dots. (B) Gene expression of m^1^A‐related molecules in HBV+ and HBV‐ cells. (C) Gene expression of *TRMT6* in HBV‐high and HBV‐low cells. (D) The mRNA expression of *TRMT6* was analyzed in HBV‐ and HBV+ HCC tissues. *β‐actin *was used as a reference gene. (E) The representative IHC staining of TRMT6 in HBV‐ and HBV+ HCC tissues. Scale bar, 50 µm. (F) Survival curves of HBV+ HCC patients with high and low expression of *TRMT6*. (G) The knockdown and overexpression of TRMT6 in HepG2.2.15 cells were confirmed by western blotting analysis in left panel. β‐Actin was used as a loading control. The m^1^A levels were detected by dotblot assay in right panel, and methylene blue staining (MB) serves as the loading control. TRMT6 was knocked down (H) and ectopically expressed (I) in HepG2.2.15 cells. Then, the expression of pgRNA was analyzed by qRT‐PCR (left panel), the levels of cccDNA were measured by TaqMan‐qPCR (middle panel) and the ratio of pgRNA/cccDNA was calculated (right panel). TRMT6 was knocked down (J) and overexpressed (K) in HepG2.2.15 cells, the levels of HBV DNA in cellular supernatant were then measured using a commercial kit. (L) TRMT6 was knocked down in HepG2.2.15 cells, and its effect on mRNA and protein levels of HBx was then evaluated by qRT‐PCR (left panel) and western blotting (right panel) assays. (M) The effect of TRMT6 overexpression on mRNA and protein levels of HBx was assessed by qRT‐PCR (left panel) and western blotting (right panel) assays in HepG2.2.15 cells. *β‐actin* was used as the internal control for qRT‐PCR and TaqMan‐qPCR, and β‐Actin was used as a loading control for western blotting analysis. Each experiment was done with three biological replicates. The data were shown as the mean ± SD. *, *P* < 0.05; **, *P* < 0.01; ***, *P* < 0.001; *ns*, no significance.

HBV infection is a major risk factor for HCC and the incidence of HCC elevates with increasing serum levels of HBV DNA [23]. Considering that effective antiviral therapies can reduce the recurrence rate of HCC and improve the prognosis of HBV‐related HCC patients [24, 25]. Thus, we attempted to determine the potential role of TRMT6‐mediated m^1^A modification in modulating HBV replication. According to HBV infection status, cells were divided into HBV‐positive (HBV+), HBV‐negative (HBV‐) and unknown subgroups (NA) (Figure 3A). We then analyzed the expression of m^1^A‐associated regulators between HBV+ and HBV‐ subgroups. Most m^1^A writers and readers were up‐regulated in the HBV+ subgroup relative to the HBV‐ subgroup, whereas m^1^A erasers exhibited the opposite expression pattern (Figure 3B). Among them, *TRMT6* was expressed at significantly higher levels in high‐titer HBV tissues than in low‐titer HBV tissues and adjacent non‐cancerous tissues (Figure 3C). We also analyzed the mRNA and protein levels of TRMT6 in fresh frozen or paraffin‐embedded HBV+ HCC samples and their control subjects. The results of qRT‐PCR (Figure 3D) and IHC (Figure 3E; Figure S4) further confirmed that TRMT6 was elevated in HBV+ HCC samples. Kaplan‐Meier survival analysis indicated that high *TRMT6* expression was correlated with poor prognosis in HBV+ HCC patients (Figure 3F; Figure S5).

We next knocked down TRMT6 (Figure 3G, left panel; Figure S6A) in HepG2.2.15 cells, which contains two copies of HBV DNA sequence head‐to‐tail integrated into their genome, and expectedly found a significant decrease in m^1^A levels in TRMT6‐knockdown cells compared to control cells (Figure 3G, right panel). Given that pgRNA transcription is crucial for HBV replication and even determines its rate [26], we thus measured the levels of pgRNA and HBV cccDNA to determine the impact of TRMT6 knockdown on the efficiency of pgRNA transcription (pgRNA/cccDNA) in HepG2.2.15 and HepAD38 cells. The results demonstrated that TRMT6 knockdown significantly decreased pgRNA expression (Figure 3H, left panel; Figure S6B, left panel) but not affect HBV cccDNA levels (Figure 3H, middle panel; Figure S6B, middle panel), thus reducing pgRNA transcription efficiency (Figure 3H, right panel; Figure S6B, right panel). Conversely, ectopic expression of TRMT6 (Figure S6C) increased pgRNA transcription efficiency (Figure 3I, right panel; Figure S6D, right panel), as indicated by elevated pgRNA levels (Figure 3I, left panel; Figure S6D, left panel) and unchanged HBV cccDNA levels (Figure 3I, middle panel; Figure S6D, middle panel). Additionally, TRMT6 knockdown decreased the content of HBV DNA in virions released into cellular supernatant (Figure 3J; Figure S6E), while TRMT6 overexpression increased HBV DNA levels (Figure 3K; Figure S6F). Expectedly, both mRNA and protein levels of HBx were markedly decreased in TRMT6‐knockdown cells (Figure 3L; Figure S6G,H) and increased in TRMT6‐overexpressing cells (Figure 3M; Figure S6I,J). These data suggest that TRMT6‐mediated m^1^A modification facilitates HBV replication, thereby increasing the oncogenic risk of HCC.

### 2.4 TRMT6 Up‐Regulates CDK9 in HCC

**FIGURE 4:**
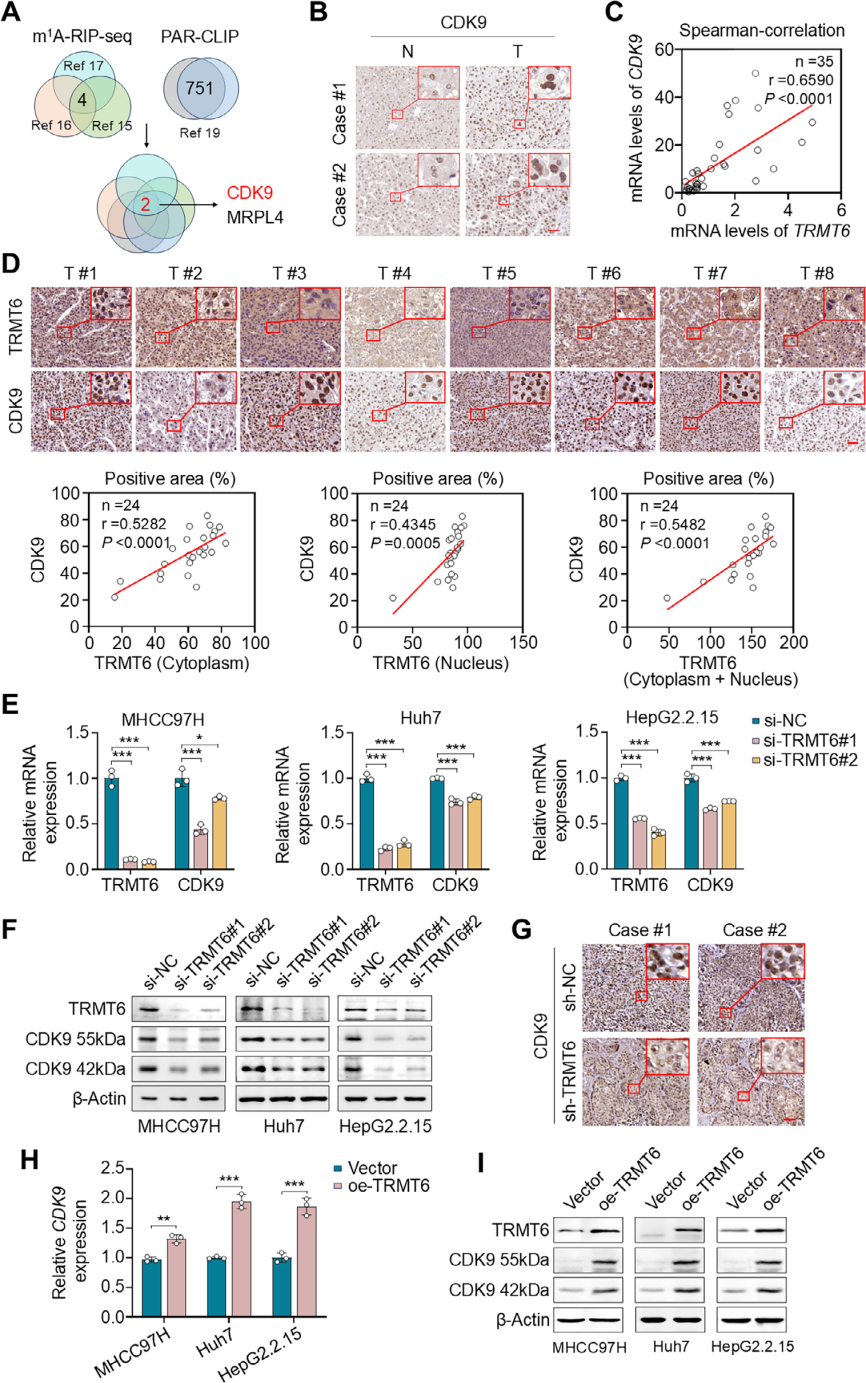
CDK9 is up‐regulated by TRMT6. (A) The Venn diagram showing the analysis of m^1^A containing mRNA transcripts or segments which obtained from three m^1^A‐RIP‐seq datasets (m^1^A‐maps of HepG2 and Hela cells, m^1^A sites accumulated after overexpression of TRMT6/TRMT61A and m^1^A peaks induced by H_2_O_2_) and two PAR‐CLIP datasets (YTHDF2 and YTHDC1‐binding sites). (B) The representative IHC staining of CDK9 in HCC tissues and control tissues. The correlation analysis between mRNA (C) and protein (D) levels of CDK9 and TRMT6 in HCC tissues. TRMT6 was knocked down in MHCC97H, Huh7 and HepG2.2.15 cells, and its effect on mRNA and protein levels of CDK9 was assessed by qRT‐PCR (E) and western blotting (F) assays. (G) The representative IHC staining of CDK9 in the indicated xenograft tumors. Scale bar, 100 µm. TRMT6 was overexpressed in MHCC97H, Huh7, and HepG2.2.15 cells, and its effect on mRNA and protein levels of CDK9 was evaluated by qRT‐PCR (H) and western blotting (I) assays. *β‐actin *was used as the internal control for qRT‐PCR, and β‐Actin was used as a loading control for western blotting analysis. Each assay was done with three biological replicates. The data were shown as the mean ± SD. *, *P* < 0.05; **, *P* < 0.01; ***, *P* < 0.001.

Given that TRMT6 is involved in m^1^A modification of mRNA [16], we next investigate whether TRMT6 promotes the malignant phenotypes of HCC cells by regulating m^1^A modification in mRNAs of major oncogenes. To identify the mRNA transcripts regulated by m^1^A modification, we analyzed three m^1^A‐RIP‐seq datasets derived from genome‐wide maps of m^1^A in HepG2 and Hela cells [17], m^1^A sites accumulated upon overexpression of TRMT6/TRMT61A [16] and m^1^A peaks induced by H_2_O_2_ [15], as well as two PAR‐CLIP datasets derived from YTHDF2 and YTHDC1‐binding m^1^A sites [19], finding that only cyclin‐dependent kinase 9 (CDK9) and mitochondrial ribosomal protein L4 (MRPL4) were consistently identified in all datasets (Figure 4A). MRPL4, a key component of the mitochondrial ribosome [27], remains poorly characterized in human malignancies, particularly in HCC. Nevertheless, we observed that neither mRNA nor protein levels of MRPL4 were altered upon TRMT6 knockdown (Figure S7A,B). CDK9, as a canonical transcriptional CDK [28], has been widely documented to exhibit oncogenic activity in various malignancies, including HCC [29]. Its inhibitor has also been reported to repress HBV replication [30]. This was supported by IHC staining showing that CDK9 expression was elevated in HCC tissues compared to control tissues (Figure 4B; Figure S8A). We further validated these results using the TCGA database (Figure S8B). Survival analysis revealed that high *CDK9* expression was significantly associated with shorter 50‑month disease‑free survival in patients with HCC (Figure S8C). Notably, this adverse prognostic correlation was further pronounced in HBV‑positive HCC patients (Figure S8D).

We next identified a significant positive correlation between mRNA expression of *CDK9* and *TRMT6* in HCCs (Figure 4C; Figure S9). As supported, the protein levels of CDK9 were positively correlated with TRMT6 located in the cytoplasm, nucleus, and whole cell in 24 paraffin‐embedded HCC samples (Figure 4D; Figure S10), further validating the above conclusion. To determine the regulatory effect of TRMT6 on CDK9, we knocked down TRMT6 in MHCC97H, Huh7, and HepG2.2.15 cells, and found that TRMT6 knockdown caused a prominent reduction in both mRNA (Figure 4E) and protein (Figure 4F) levels of CDK9, which has two isoforms with molecular weights of 55 and 42 kDa [31]. This observation was corroborated by IHC staining of CDK9 in xenograft tumor tissues (Figure 4G; Figure S11). Conversely, ectopic overexpression of TRMT6 in these cells up‐regulated the mRNA (Figure 4H) and protein (Figure 4I) levels of CDK9. To exclude the potential involvement of m^6^A modification in regulating CDK9 expression, we treated MHCC97H, Huh7, and HepG2.2.15 cells with the METTL3 inhibitor STM2457, and failed to find consistent downregulation of CDK9 expression, as compared with that observed after TRMT6‐knockdown (Figure S12). Collectively, these data suggest that TRMT6 may regulate CDK9 expression in an m^1^A‐dependent manner.

### 2.5 TRMT6‐Mediated m^1^A Modification of *CDK9* mRNA Enhances Its Stability and Translation Efficiency

**FIGURE 5:**
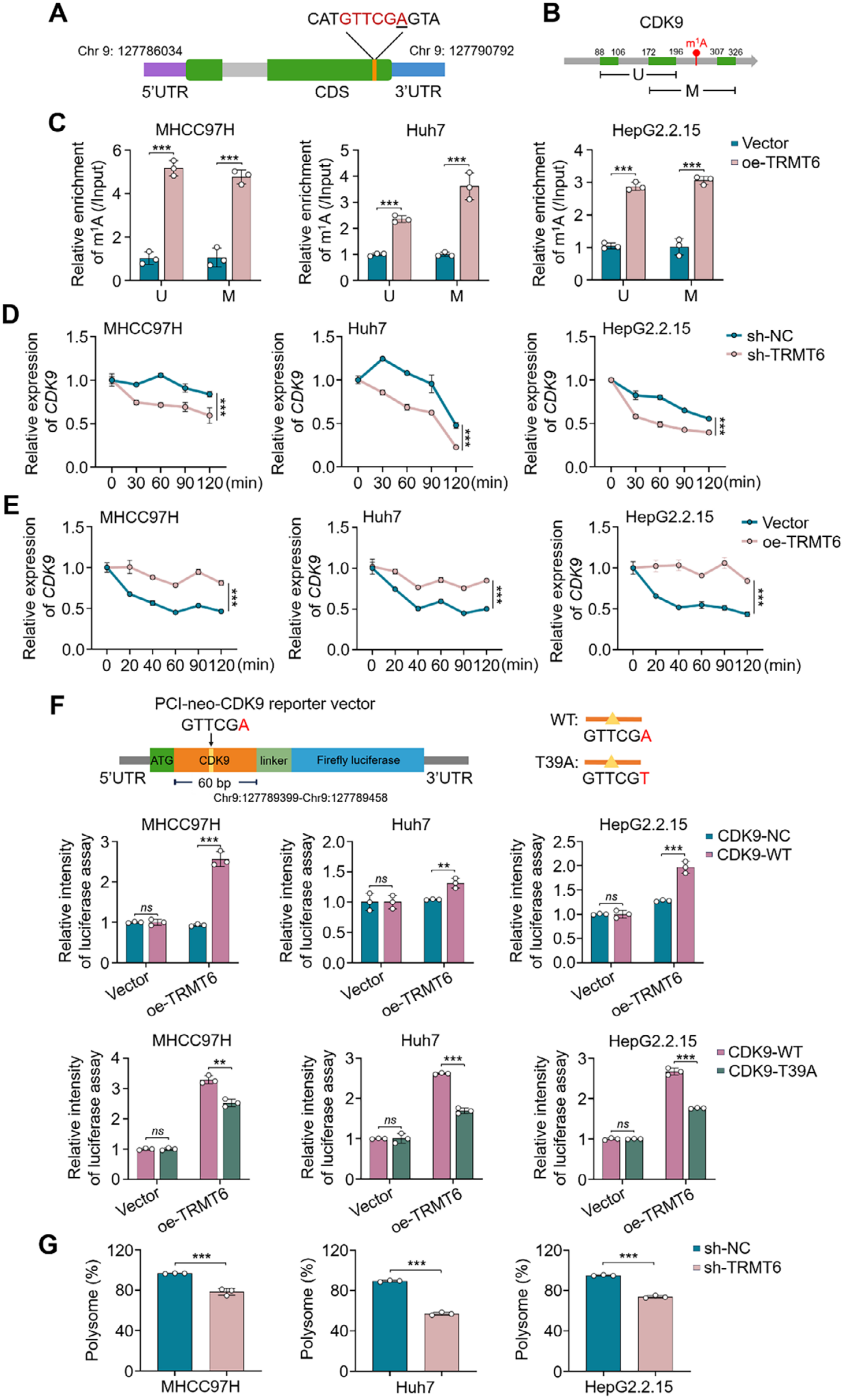
TRMT6‐mediated m^1^A modification enhances the stability and translation efficiency of *CDK9* mRNA. (A) Schematic m^1^A‐containing sequence in the CDS of *CDK9* mRNA. The m^1^A motif was marked in red, and the m^1^A site was underlined. (B) Schematic two amplified fragments (U and M) upstream and across the m^1^A site in MeRIP‐qPCR. The m^1^A site was marked in red. (C) TRMT6 was overexpressed in MHCC97H, Huh7, and HepG2.2.15 cells, and its effect on the relative enrichment of m^1^A was evaluated by MeRIP‐qPCR. Input was used as the normalization control. TRMT6 was knocked down (D) and overexpressed (E) in MHCC97H, Huh7, and HepG2.2.15 cells. After 48 h, 10 µM ACTD was supplemented to the culture medium of the above cells. The mRNA levels of *CDK9* at the indicated time points were measured by qRT‐PCR. *18S* rRNA was used as the normalization control. (F) Schematic dual‐luciferase reporter plasmids containing wild‐type (WT) or mutant m^1^A site (T39A) (upper panel). TRMT6 were overexpressed in MHCC97H, Huh7, and HepG2.2.15 cells. After 24 h, the above cells were transfected with CDK9‐NC (control) and CDK9‐WT plasmids (middle panels) or CDK9‐WT and CDK9‐T39A plasmids (lower panels) for 48 h. Dual‐luciferase reporter assays were then performed to measure the luciferase intensity with the intensity of Renilla luciferase as normalization control. (G) TRMT6 was knocked down in the indicated HCC cells for 48 h, the equal‐OD cell lysis was then subjected to sucrose gradient density centrifugation. The levels of *CDK9* mRNA in all the gradients were separated and detected by qRT‐PCR, and the proportion of *CDK9* mRNA in polysome gradients was calculated. *β‐actin *was used as the internal control. Each experiment was performed with three biological replicates. The data were shown as the mean ± SD. *, *P* < 0.05; **, *P* < 0.01; ***, *P* < 0.001; *ns*, no significance.

We next used RMBase v2.0 database (https://rna.sysu.edu.cn/rmbase/m1Amod.php) to predict the m^1^A motif in *CDK9* mRNA, and found a m^1^A motif “GTTCGA” in its coding DNA sequence (CDS) (Figure 5A). Methylated RNA immunoprecipitation (MeRIP) coupled with qRCR (MeRIP‐qPCR) can be used to determine the levels of m^1^A modification [17]. Thus, we first designed two pairs of primers to detect the m^1^A upstream sequence “U” and m^1^A‐containing sequence “M” in *CDK9* mRNA (Figure 5B). MeRIP‐qPCR results revealed that ectopic expression of TRMT6 in MHCC97H, Huh7, and HepG2.2.15 cells substantially increased m^1^A levels (Figure 5C).

RNA methylation modifications, such as m^6^A and m^1^A, usually affect the stability and translation efficiency of mRNA [14]. Thus, we treated TRMT6‐knockdown or ‐overexpressing MHCC97H, Huh7, and HepG2.2.15 cells as well as their control cells with 10 µM actinomycin D (ACTD) to inhibit mRNA transcription, and then evaluated their effect on the stability of *CDK9* mRNA. The results demonstrated that TRMT6 knockdown strikingly accelerated the decay of *CDK9* mRNA compared to the control (Figure 5D), whereas TRMT6 overexpression enhanced *CDK9* mRNA stability (Figure 5E). We also constructed luciferase reporter plasmids containing either wild‐type m^1^A motif (GTTCGA) or a point‐mutant m^1^A motif (GTTCGT) based on the pCI‐neo vector (Figure 5F, upper panel), which were named as CDK9‐WT and ‐T39A, respectively. The data revealed that TRMT6 overexpression significantly increased the intensity of firefly luciferase of CDK9‐WT reporter plasmid (Figure 5F, middle panels), while CDK9‐T39A substantially alleviated this effect (Figure 5F, lower panels), indicating that m^1^A modification boosted the translation of *CDK9* mRNA. As supported, we demonstrated that TRMT6 knockdown markedly decreased the distribution of *CDK9* mRNA in polysome layers in MHCC97H, Huh7, and HepG2.2.15 cells using polysome profiling assays (Figure 5G; Figure S13). We next screened multiple established m^1^A readers via siRNA‐mediated knockdown. The results showed that none of these known readers significantly regulated CDK9 expression at either the mRNA or protein level across the three cell lines examined (Figure S14). These results led us to reasonably infer that the m^1^A reader responsible for mediating CDK9 regulation is likely not among currently identified ones, and may be a novel reader with dual roles in mRNA stability and translation regulation. These data, taken together, indicate that TRMT6‐mediated m^1^A modification of *CDK9* mRNA enhances its stability and translation efficiency.

### 2.6 CDK9 Inhibition Abrogates the Promoting Effects of TRMT6 on Malignant Behaviors of HCC Cells and HBV Replication

**FIGURE 6:**
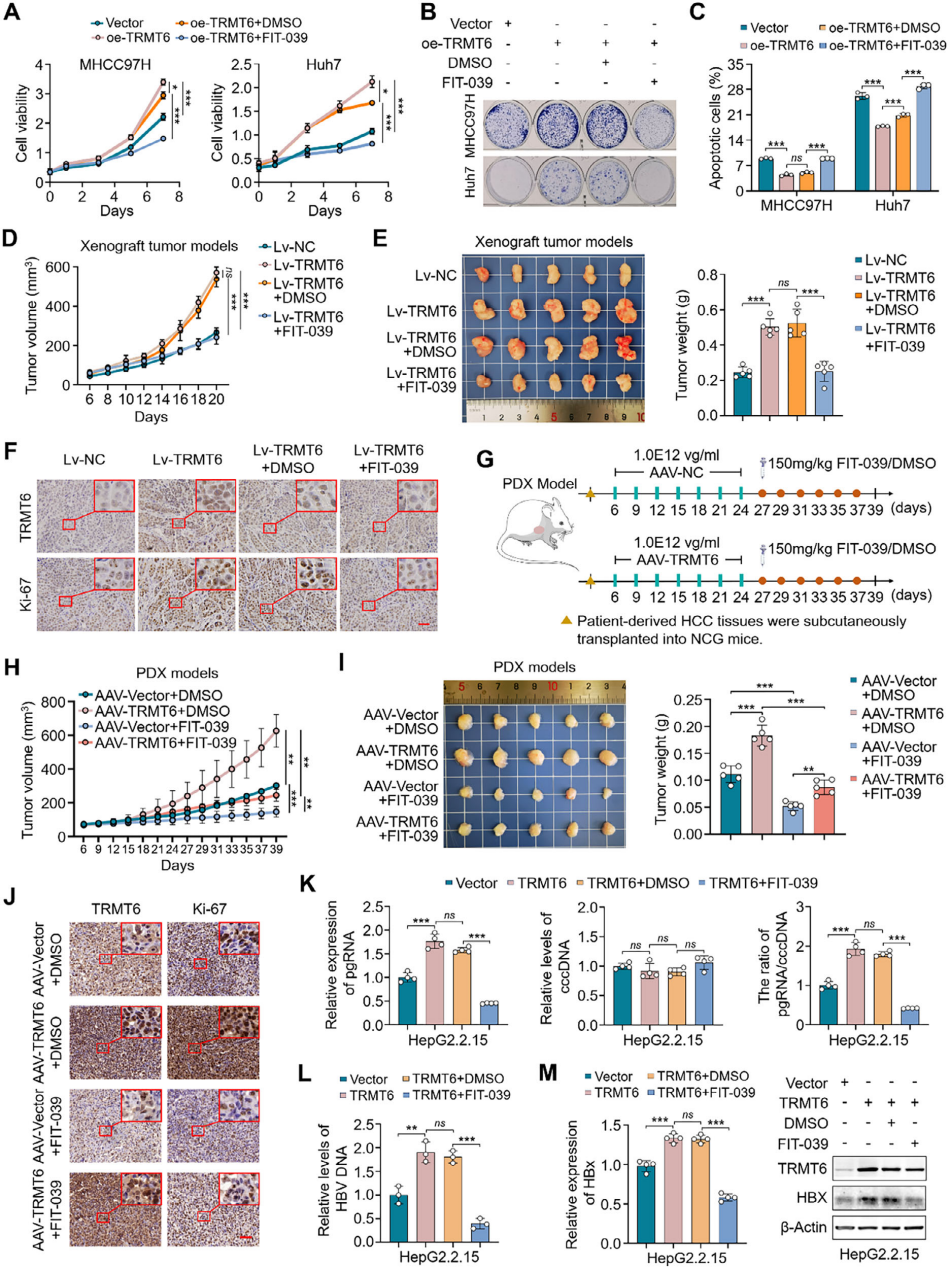
TRMT6‐mediated up‐regulation of CDK9 facilitates the malignant phenotypes of HCC cells and HBV replication. TRMT6 was overexpressed in MHCC97H and Huh7 cells. After 48 h, 10 µM FIT‐039 was added to the culture medium, and its effects on cell viability (A) colony formation (B) cell apoptosis (C) were further evaluated. (D) The growth curves of the indicated tumors (n = 5/group). (E) The images of dissected tumors (left panel) and tumor weights (right panel) of the indicated groups. (F) The representative IHC staining of TRMT6 and Ki‐67 in the indicated tumors. Scale bar, 50 µm. (G) Experimental procedures of the PDX model with TRMT6 overexpression and CDK9 inhibition. (H) The growth curves of indicated tumors from PDX models (n = 5/group). (I) The images of dissected tumors (left panel) and tumor weights (right panel) of the indicated groups from PDX models. (J) The representative IHC staining of TRMT6 and Ki‐67 in the indicated tumors from PDX models. Scale bar, 50 µm. (K) TRMT6 was overexpressed in HepG2.2.15 cells and 10 µM FIT‐039 was added to the culture medium for 48 h. Then, the levels of pgRNA (left panel) were analyzed by qRT‐PCR, the levels of cccDNA (middle panel) were measured by TaqMan‐qPCR and the ratio of pgRNA/cccDNA was calculated (right panel). TRMT6 was overexpressed in HepG2.2.15 cells, and 10 µM FIT‐039 was then added to the culture medium for 48 h. Next, the content of HBV DNA in cellular supernatant was detected using commercial kit (L), and mRNA and protein levels of HBx were analyzed by qRT‐PCR (left panel) and western blotting (right panel) assays (M). *β‐actin* was used as internal control for qRT‐PCR and TaqMan‐qPCR, and β‐Actin was used as a loading control for western blotting analysis. Each assay was done with three biological replicates. The data were shown as the mean ± SD. *, *P* < 0.05; **, *P* < 0.01; ***, *P* <0.001; *ns*, no significance.

To further define the oncogenic function of TRMT6‐mediated mRNA m^1^A modification in HCC via up‐regulation of CDK9, we first treated MHCC97H and Huh7 cells with the selective CDK9 inhibitor FIT‐039, showing that IC_50_ values of FIT‐039 were 22.13 µM in MHCC97H cells and 13.93 µM in Huh7 cells (Figure S15). Meanwhile, we observed that FIT‑039 treatment markedly reversed the pro‑proliferative and colony‑forming effects induced by TRMT6 overexpression (Figure 6A,B). Consistently, CDK9 knockdown in TRMT6‑overexpressing MHCC97H and Huh7 cells similarly attenuated the enhancing effects of TRMT6 on cell proliferation and colony formation (Figure S16). Furthermore, both FIT‑039 treatment and CDK9 silencing efficiently abrogated the suppressive effect of TRMT6 overexpression on cell apoptosis (Figure 6C; Figures S17 and S18).

To further investigate the impact of TRMT6‐mediated up‐regulation of CDK9 on the tumorigenicity of HCC cells in vivo, we stably overexpressed TRMT6 in MHCC97H cells using lentivirus system, and subcutaneously inoculated TRMT6‐overexpressing cells along with their control cells into nude mice. The mice bearing TRMT6‐overexpressing cell‐derived xenograft tumors were then administrated with 150 mg/kg FIT‐039 or the equal dose of DMSO (Figure S19A). The results showed that TRMT6‐overexpressing cell‐derived xenograft tumors grew faster and weighted more compared to control tumors, which were effectively reversed by FIT‐039 (Figure 6D,E). This was supported by IHC staining of nuclear proliferative marker Ki‐67 in the above tumors (Figure 6F; Figure S19B). Furthermore, no significant intergroup differences in body weight were observed throughout the treatment period (Figure S19C). Similarly, serum markers and histomorphology of hepatic and renal function remained unaltered in these mice (Figure S20A,B). Collectively, these results indicate that FIT‑039 administration does not elicit overt toxicity.

To improve the clinical relevance and translational potential of our findings, we constructed HCC patient‐derived xenograft (PDX) models by subcutaneously implanting human HCC tissues into NCG mice, and overexpressed TRMT6 via intratumoral injection of adeno‐associated virus (AAVs). Tumor‑bearing mice were then administered 150 mg/kg FIT‑039 or an equivalent dose of DMSO via intraperitoneal injection (Figure 6G). The results showed that pharmacological inhibition of CDK9 markedly suppressed tumor growth in PDX models, as reflected by reduced tumor volume and weight (Figure 6H,I). IHC staining of Ki‐67 further supported the above results (Figure 6J; Figure S21A). Throughout the treatment period, mouse body weights remained comparable across all experimental groups (Figure S21B). Moreover, serum biochemical profiles (Figure S22A) and H&E staining of liver and kidney sections (Figure S22B) demonstrated that FIT‑039 treatment did not elicit appreciable toxicity.

We next treated TRMT6‐overespressing HepG2.2.15 cells with 10 µM FIT‐039 to evaluate its effect on HBV replication. In parallel, CDK9 was knocked down in TRMT6‐overexpressing HepAD38 cells to exclude potential off‐target effect of FIT‐039 (Figure S23). The results showed that both FIT‐039 and CDK9 silencing effectively reversed TRMT6‐mediated increase in pgRNA transcription efficiency (Figure 6K, right panel; Figure S24A, right panel), as reflected by restored pgRNA levels (Figure 6K, left panel; Figure S24A, left panel) and unaltered cccDNA levels (Figure 6K, middle panel; Figure S24A, middle panel). Furthermore, the stimulatory effects of TRMT6 overexpression on the levels of HBV DNA (Figure 6L; Figure S24B) and HBx (Figure 6M; Figure S24C) could be similarly abrogated by FIT‐039 or CDK9 knockdown. Collectively, our findings support that TRMT6‐mediated m^1^A modification facilitates HCC progression and HBV replication via up‐regulating CDK9, underscoring their potential as the therapeutic targets in for HBV‐related HCC.

### 2.7 CDK9 Enhances the Malignant Phenotypes of HCC Cells by Elevating Its Downstream Oncogenic Effectors

**FIGURE 7:**
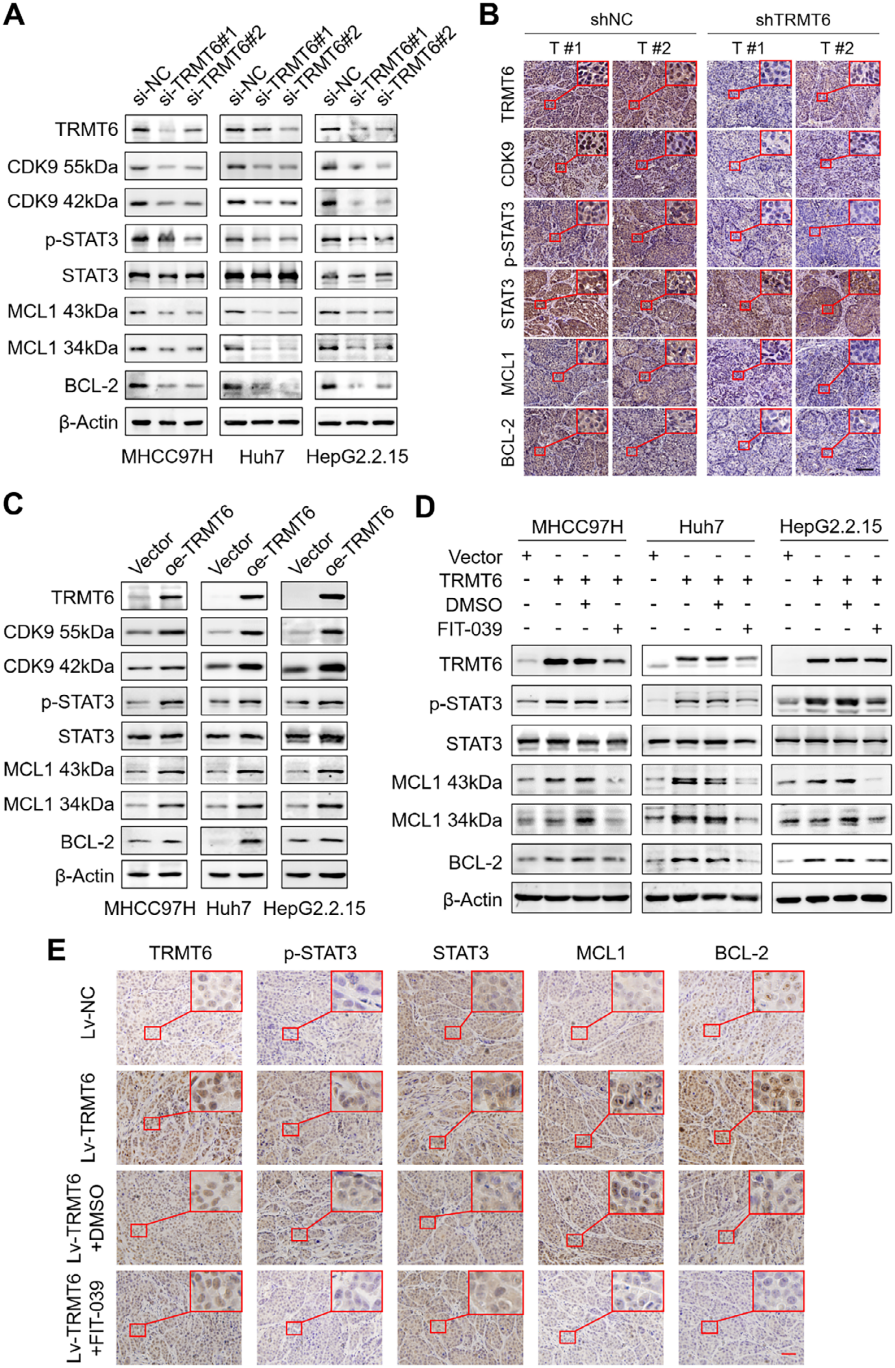
Oncogenic role of TRMT6 in HCC via CDK9‐mediated up‐regulation of oncogenic effectors. (A) TRMT6 was knocked down in MHCC97H, Huh7, and HepG2.2.15 cells, and the effect on the levels of CDK9, p‐STAT3, MCL1, and BCL‐2 was then evaluated by western blotting analysis. β‐Actin was used as a loading control. (B) The representative IHC staining of TRMT6, CDK9, p‐STAT3, MCL1, and BCL‐2 in the indicated tumors. Scale bar, 50 µm. (C) TRMT6 was overexpressed in MHCC97H, Huh7, and HepG2.2.15 cells, and their effect on the levels of CDK9, p‐STAT3, MCL1, and BCL‐2 was then evaluated by western blotting analysis. β‐Actin was used as a loading control. (D) TRMT6 was overexpressed in MHCC97H, Huh7, and HepG2.2.15 cells. Then, 10 µM CDK9 inhibitor FIT‐039 was added to the culture medium for 48 h, and their effect on the levels of the above molecules was assessed by western blotting analysis. β‐Actin was used as a loading control. (E) The representative IHC staining of TRMT6, p‐STAT3, MCL1, and BCL‐2 in the indicated tumors. Scale bar, 50 µm. Each experiment was performed with three biological replicates.

As a serine/threonine (Ser/Thr) kinase, CDK9 exerts its oncogenic activity by up‐regulating key downstream effectors [32]. We thus investigated the impact of TRMT6 silencing or overexpression on the levels of key oncogenic downstream targets of CDK9, including phosphorylated STAT3 (p‐STAT3), MCL1 and BCL‐2, which are widely recognized as critical mediators of HCC pathogenesis [33, 34, 35], in MHCC97H, Huh7, and HepG2.2.15 cells. The results expectedly showed that TRMT6 knockdown markedly reduced the levels of these oncogenic proteins (Figure 7A), which was further validated by IHC staining in TRMT6‐knockdown xenograft tumors and control tumors (Figure 7B, Figure S25A). Conversely, TRMT6 overexpression elevated the levels of p‐STAT3, MCL1 and BCL‐2 (Figure 7C), and this effect was reversed by both FIT‐039 and CDK9 knockdown (Figure 7D; Figure S25B). These observations were further corroborated by IHC staining of these molecules in xenograft tumors from Figure 6E,I (Figure 7E; Figures S25C and S26). These data collectively establish that TRMT6‑mediated up‐regulation of CDK9 drives HCC progression by activating its core pro‑tumorigenic downstream effectors.

### 2.8 CDK9 Facilitates HBV Replication Through Phosphorylating TARDBP at Ser254

Although CDK9 inhibition can hinder HBV replication [30], the underlying molecular mechanism remains poorly defined. Given that CDK9 functions as a kinase primarily by interacting with and phosphorylating its substrate proteins [36], we performed co‐immunoprecipitation (Co‐IP) coupled with liquid chromatography‐tandem mass spectrometry (LC‐MS/MS) to identify potential CDK9 substrates in HepG2.2.15 and HepG2 cells. Gene Ontology (GO) analysis of the candidate proteins in HepG2.2.15 cells revealed 15 terms associated with viral processes and life cycles (Figure S27A; Table S1). We further analyzed the top‐10 up‐regulated and down‐regulated molecules involved in viral process by comparing differential unique peptides between HepG2.2.15 and HepG2 cells (Figure S27B; Table S2). Among them, TAR DNA binding protein (TARDBP, also known as TDP‐43), a nuclear DNA/RNA‐binding protein implicated in mRNA transcription, splicing, stability and translational regulation [37], has been previously linked to HBV replication [38]. Its peptide sequence identified by LC‐MS/MS was showed in Figure S28. Therefore, TARDBP was selected for in‐depth mechanistic characterization in subsequent experiments.

**FIGURE 8:**
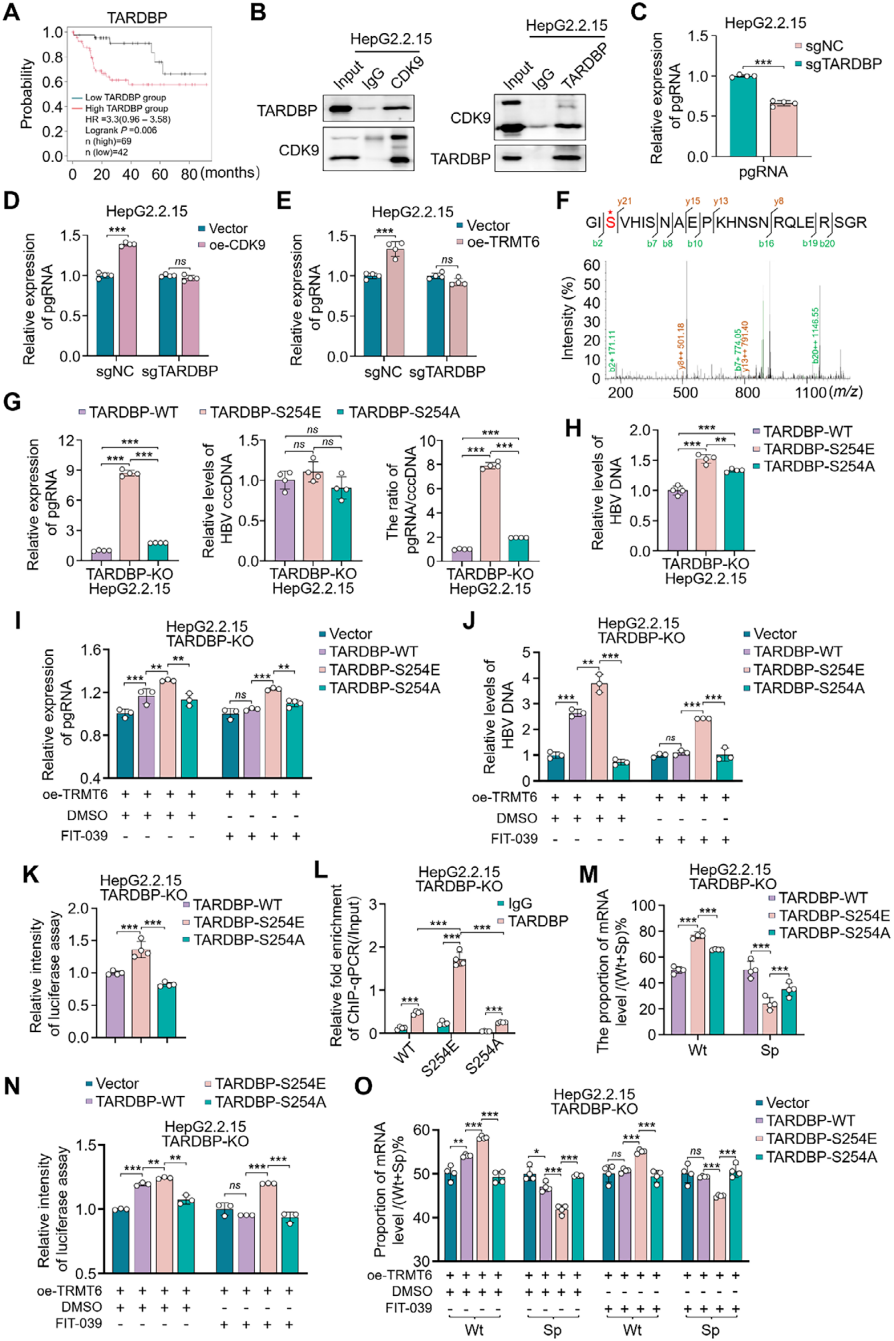
CDK9 facilitates HBV replication via the phosphorylation of TARDBP at Ser254. (A) The survival curves of HBV+ HCC patients with high and low expression of *TARDBP*. (B) Co‐IP was performed to validate the interaction between CDK9 and TARDBP in HepG2.2.15 cells. (C) TARDBP was knocked out in HepG2.2.15 cells, and the mRNA expression of pgRNA was measured by qRT‐PCR. CDK9 (D) and TRMT6 (E) was overexpressed in TARDBP‐knockout HepG2.2.15 cells, the levels of pgRNA were then evaluated by qRT‐PCR. (F) The representative image of unique peptide containing the phosphorylation of TARDBP at Ser254. (G) TARDBP was knockout in HepG2.2.15 cells, and the plasmids with TARDBP wild type (WT), simulated phosphorylation‐activated mutation of S254 (S254E) and simulated phosphorylation‐inactivated mutation of S254 (S254A) were then transfected into the above cells. Then, the levels of pgRNA (left panel) were evaluated by qRT‐PCR, the levels of cccDNA (middle panel) were measured by TaqMan‐qPCR, and the ratio of pgRNA/cccDNA was calculated (right panel). (H) TARDBP‐knockout HepG2.2.15 cells were transfected with TARDBP‐WT, ‐S254E, and ‐S254A plasmids. Their effect on the content of HBV DNA in cellular supernatant was then determined using commercial kits. (I) TRMT6 as well as TARDBP‐WT, ‐S254E, and ‐S254A plasmids were co‐transfected into TARDBP‐knockout HepG2.2.15 cells for 24 h. Then, 10 µM FIT‐039 was added to the culture medium for a further 48 h. The levels of pgRNA were evaluated by qRT‐PCR and (J) the levels of HBV DNA were detected by the commercial kit. *β‐actin* was used as internal control of qRT‐PCR. (K) TARDBP was knocked out in HepG2.2.15 cells, and TARDBP‐WT, ‐S254E, and ‐S254A plasmids and luciferase reporter plasmid containing the HBV core promoter were then transfected into HepG2.2.15 cells for 48 h. Dual‐luciferase reporter assays were then performed to assess the luciferase intensity with the intensity of Renilla luciferase as normalization control. (L) TARDBP was knocked out in HepG2.2.15 cells, and TARDBP‐WT, ‐S254E, and ‐S254A plasmids were transfected into the above cells for 48 h. Their effect on the interaction of TARDBP with the HBV core promoter was determined by ChIP‐qPCR. Input was used as normalization control. (M) The plasmids containing TARDBP‐WT, ‐S254E and ‐S254A were transfected into TARDBP‐knockout HepG2.2.15 cells for 48 h. The relative levels of wild type (Wt) pgRNA and splicing variant (Sp) were evaluated by qRT‐PCR. The proportions of mRNA level of Wt and Sp were then calculated. (N) TRMT6 was overexpressed in TARDBP‐knockout HepG2.2.15 cells, meanwhile, TARDBP‐WT, ‐S254E, and ‐S254A plasmids were transfected into these cells for 24 h. Then, cells were further transfected with the luciferase plasmids containing the HBV core promoter and suppled with 10 µM FIT‐039 for 48 h. Dual‐luciferase reporter assays were performed to determine their effect on the luciferase intensity, with the intensity of Renilla luciferase as normalization control. (O) TRMT6 was overexpressed in TARDBP‐knockout HepG2.2.15 cells, and TARDBP‐WT, ‐S254E, and ‐S254A plasmids were then transfected into these cells for 24 h. Next, 10 µM FIT‐039 was added to the cultural medium for 48 h. The mRNA levels of Wt and Sp were analyzed by qRT‐PCR, and the proportion of Wt and Sp was calculated. *β‐actin* was used as internal control of qRT‐PCR. Each assay was done with three biological replicates. The data were shown as the mean ± SD. *, *P* < 0.05; **, *P* < 0.01; ***, *P* < 0.001; *ns*, no significance.

We initially interrogated *TARDBP* mRNA levels via The Cancer Genome Atlas (TCGA) database, which demonstrated a marked and significant increase in *TARDBP* expression in HCC tissues relative to control tissues (Figure S29A). Subsequent survival analysis indicated a strong correlation between elevated TARDBP expression and poor clinical outcomes in HCC patients, with this association being more pronounced in HBV‐positive HCC cases (Figure S29B; Figure 8A). Also, we verified the interaction between CDK9 and TARDBP in HepG2.2.15 cells by reciprocal Co‐IP assays (Figure 8B). We next knocked out TARDBP in HepG2.2.15 and HepG2‑NTCP cells via CRISPR‑Cas9 system. HepG2‑NTCP cells were established by ectopic expression of NTCP in HepG2 cells (Figure S30A,B), with successful TARDBP depletion confirmed in Figure S30C. TARDBP knockout elicited a striking reduction in pgRNA levels (Figure 8C; Figure S30D). Furthermore, overexpression of either CDK9 (Figure 8D) or TRMT6 (Figure 8E) robustly increased pgRNA levels in control cells, but not in TARDBP‑deficient cells, supporting a model in which the TRMT6–CDK9 axis facilitates HBV replication through TARDBP.

We next investigated how CDK9 regulates HBV replication via TARDBP. Given its kinase activity, we hypothesized that CDK9 directly phosphorylates TARDBP as a substrate. To test this, HepG2.2.15 cells were treated with the CDK9 inhibitor FIT‑039 or DMSO, followed by Co‑IP coupled with LC‑MS/MS to identify CDK9‑regulated TARDBP phosphorylation sites. FIT‑039 significantly reduced TARDBP phosphorylation at multiple serine residues, including Ser254 (Figure 8F; Figure S31; Table S3). In vitro kinase assays further validated that CDK9 directly phosphorylated TARDBP at Ser254 (Figure S32A; Tables S4 and S5), whereas this modification was abolished in the S254A mutant (Figure S32B; Table S6). The RRM2 domain (residues 192–265) of TARDBP binds the HBV core promoter (CP) [39], which is essential for pgRNA transcription [40], and Ser254 is critical for RNA binding affinity of TARDBP [41]. Together, these findings suggest that TARDBP Ser254 phosphorylation may contribute to HBV replication.

To validate the above hypothesis, we generated wild‐type TARDBP (TARDBP‐WT) and its phosphorylation‑mimetic (S254E) and phosphorylation‑deficient (S254A) mutants, each harboring codon‑synonymous mutation within the sgTARDBP recognition sequence. These constructs were then transfected into TARDBP‑knockout HepG2.2.15 and HepG2‑NTCP cells (Figure S33A), and their impacts on HBV replication were assessed. The results revealed that TARDBP‑S254E markedly increased pgRNA levels relative to TARDBP‑WT, whereas TARDBP‑S254A significantly reduced pgRNA abundance compared with TARDBP‑S254E (Figure 8G, left panel; Figure S33B, left panel). Neither mutant altered cccDNA levels (Figure 8G, middle panel; Figure S33B, middle panel), and their effects on pgRNA transcription efficiency mirrored those on pgRNA levels (Figure 8G, right panel; Figure S33B, right panel). Likewise, TARDBP‑S254E significantly elevated the levels of HBV DNA and HBx compared to TARDBP‐WT, while TARDBP‐S254A abolished these effects (Figure 8H; Figure S33C,D). To further validate these results, we ectopically expressed TRMT6 and supplemented with 10 µM FIT‐039 in TARDBP‐knockout HepG2.2.15 cells. The results indicated that TARDBP‑WT increased the levels of pgRNA and HBV DNA, which were further enhanced by TARDBP‑S254E but blunted by TARDBP‑S254A (Figure 8I,J). Notably, following FIT‑039 treatment, only TARDBP‑S254E retained the ability to strongly promote the levels of pgRNA and HBV DNA (Figure 8I,J). These data suggest that CDK9‐mediated phosphorylation of TARDBP at Ser254 facilitates HBV replication.

### 2.9 Phosphorylation of TARDBP at Ser254 Activates the HBV Core Promoter and Inhibits pgRNA Splicing

Based on the above findings, we supposed that phosphorylation of TARDBP at Ser254 promotes HBV replication probably by enhancing CP activity and inhibiting pgRNA splicing. To test this, TARDBP‐WT, ‐S254E, and ‐S254A were ectopically expressed in TARDBP‐knockout HepG2.2.15 and HepG2‐NTCP cells, and CP activity was then measured using a dual‐luciferase reporter containing the HBV CP region (nt1613–nt1849) [42]. The results showed that the intensity of firefly luciferase was significantly increased by TARDBP‐S254E compared to TARDBP‐WT, while was greatly decreased by TARDBP‐S254A in comparison with TARDBP‐S254E (Figure 8K; Figure S34A). ChIP‐qPCR assays further confirmed that TARDBP‐S254E binding to HBV CP was enhanced, whereas TARDBP‐S254A substantially attenuated this effect (Figure 8L; Figure S34B). Given that TARDBP is known to regulate pgRNA splicing [39], we examined the impact of phosphorylation of TARDBP at S254 on the levels and proportions of wild‐type (Wt) and 2.2 kb spliced variant (Sp) pgRNA lacking a 1.3‐kb intron [43], by ectopically expressing TARDBP‐WT, ‐S254E, and ‐S254A in TARDBP‐knockout HepG2.2.15 and HepG2‐NTCP cells. The results indicated that TARDBP‐S254E vastly elevated the Wt proportion and reduced its Sp proportion of pgRNA compared with TARDBP‐WT, and this effect was significantly attenuated by TARDBP‐S254A (Figure 8M; Figure S34C). We next ectopically expressed TRMT6 in TARDBP‐knockout cells and treated these cells with FIT‐039, evaluating their effects on the activity of HBV CP and pgRNA splicing. Compared with the control, the intensity of firefly luciferase (Figure 8N) and the proportion of Wt pgRNA (Figure 8O) were significantly increased by TARDBP‐WT and further enhanced by TARDBP‐S254E, while this effect was drastically reduced by TARDBP‐S254A. These results, taken together, demonstrate that the TRMT6‐CDK9 axis drives TARDBP phosphorylation at Ser254, thereby promoting HBV replication by stimulating pgRNA transcription and inhibiting pgRNA splicing.

**FIGURE 9:**
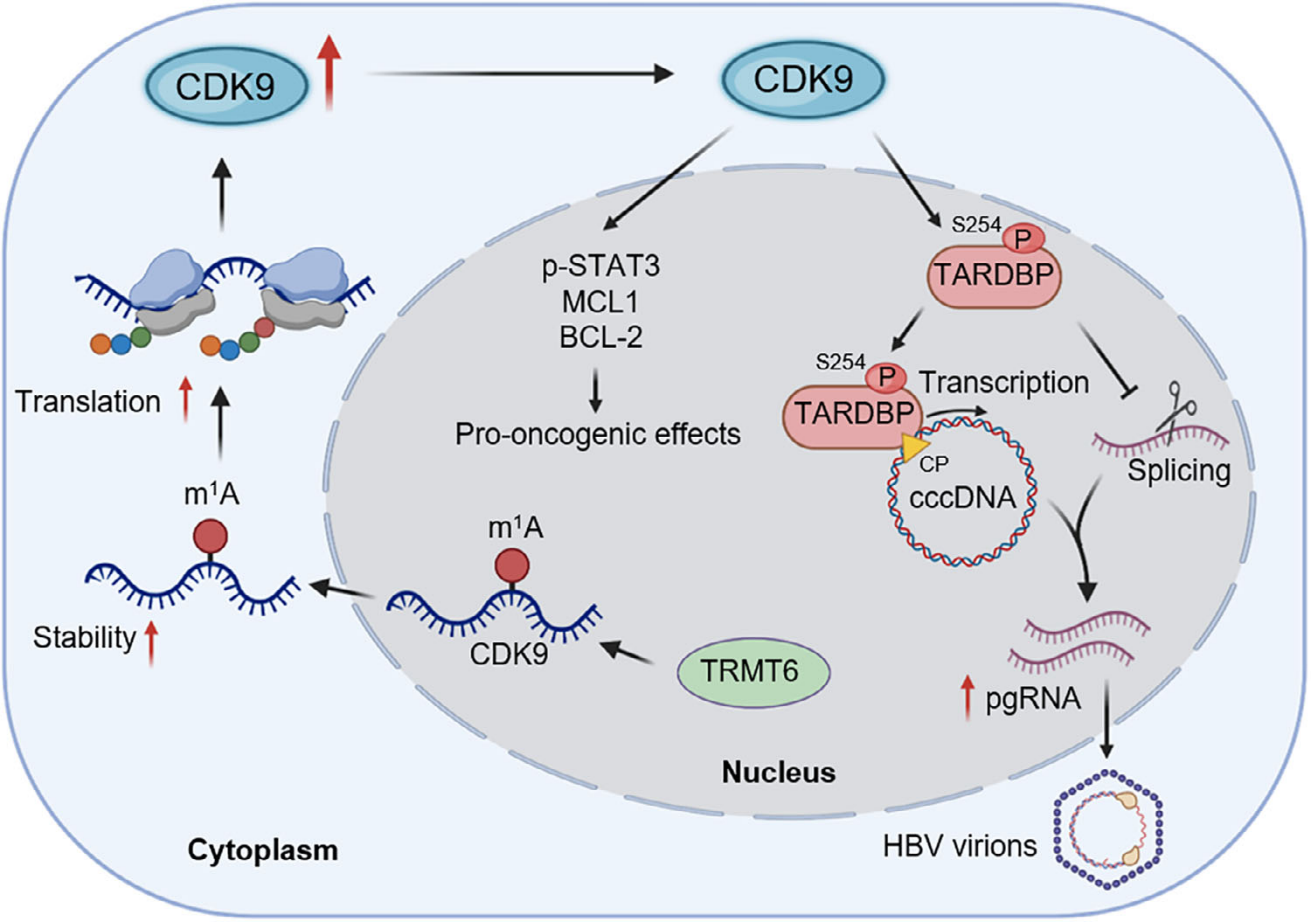
A schematic model showing the dual role of TRMT6‐mediated up‐regulation of CDK9 via m^1^A modification in HCC progression and HBV replication. Briefly, TRMT6 is highly expressed in HCCs to increase the m^1^A levels of *CDK9* mRNA, which enhances its mRNA stability and translation efficiency. As a result, CDK9 is up‐regulated in HCC. On one hand, CDK9 promotes the malignant behaviors of HCC cells by up‐regulating its downstream oncogenic effectors. On the other hand, CDK9 phosphorylated TARDBP at Ser254 to facilitate HBV replication by activating the HBV core promoter to enhance pgRNA transcription and inhibiting pgRNA splicing.

By summarizing the above findings, we elucidate the mechanism by which TRMT6‐mediated m^1^A modification promotes HCC progression and HBV replication by up‐regulating CDK9 (Figure 9). Specifically, increased expression of TRMT6 enhances the stability and translation efficiency of *CDK9* mRNA via m^1^A modification to up‐regulate its expression. CDK9, on one hand, promotes the malignant phenotypes of HCC cells by up‐regulating the levels of its downstream oncogenic effectors such as p‐STAT3, MCL1 and BCL‐2. On the other hand, CDK9 phosphorylates TARDBP at Ser254 to facilitate pgRNA transcription by activating HBV CP and inhibit pgRNA splicing, thereby facilitating HBV replication and increasing the risk of HCC.

## 3 Discussion

HCC is one of the most prevalent malignancies globally, characterized by high morbidity and mortality [2, 3]. As one of the most important factors causing HCC [5], chronic viral hepatitis B (CHB) is challenging to completely eradicate, which increases the risk of HCC [6]. However, the relationship between HCC malignant progression and HBV replication with N^1^‐methyladenosine (m^1^A) modification in mRNA remains unclear. In this study, we discovered increased levels of m^1^A modification and its “writer” TRMT6 in HBV‐infected HCC tissues by snRNA‐seq, and demonstrated that TRMT6‐mediated m^1^A modification of *CDK9* mRNA elevated its stability and translation efficiency, thereby up‐regulating CDK9 in HCC. Subsequently, TRMT6‐mediated mRNA m^1^A modification serves as a dual role in facilitating HCC progression and HBV replication through upregulating CDK9. Thus, targeting this process may become an effective way for the treatment of HBV‐related HCC.

RNA modifications were acknowledged to play crucial roles in tumors [14]. Among them, RNA methylation modification like m^6^A has been well studied in different types of cancers [44], while the functions of m^1^A in comparison with m^6^A has received limited attention, especially in mRNAs. In this study, we discovered that the m^1^A levels within the “GTTCGA” motif in the CDS of *CDK9* mRNA were regulated by TRMT6, indicating that CDK9 is a downstream target of TRMT6. Furthermore, we demonstrated that the m^1^A modification in this site facilitated the stability and translation efficiency of *CDK9* mRNA, thereby resulting in CDK9 up‐regulation in HCC. It is widely acknowledged that mRNA methylation functions by interacting with “reader” proteins. Therefore, we hypothesize that m^1^A‐modified *CDK9* mRNA may recruit a “reader” protein to modulate its degradation. Also, m^1^A in mRNA may change the interaction between mRNA and translation factors, leading to the enhancement of translation process [17]. However, we screened known m^1^A “reader” proteins, including YTHDF1‐3 and YTHDC1 [19], but did not find that they were able to regulate the mRNA and protein levels of CDK9. We therefore speculate that either multiple m^1^A readers cooperate in the multistep m^1^A regulatory process, or the specific reader mediating this function has not yet been identified. To identify the specific m^1^A “reader” proteins governing the mRNA stability and translation efficiency of *CDK9*, we plan to synthesize m^1^A‐modified *CDK9* mRNA fragments corresponding to endogenous modification sites and perform RIP assay to enrich interacting proteins in future studies, enabling the identification of candidate m^1^A readers.

As a pivotal kinase responsible for transcription elongation and mRNA maturation, CDK9 is overexpressed and activated in various relevant pathologic process, such as cardiovascular disease, cancer and viral replication [32]. In HCC, CDK9 activity is elevated due to frequent inactivation of tumor suppressor genes or aberrant CDK activation [45]. It has been reported that multiple post‐translational modifications such as dephosphorylation and phosphorylation participate in the regulation of CDK9 activity [32]. Additionally, mRNA expression of *CDK9* is also regulated by microRNAs (miRNAs) like miR‐206 [46]. However, the regulatory effect of other mechanisms, such as RNA methylation, on CDK9 expression has rarely been studied. Here, we uncover a novel post‐transcriptional regulatory mechanism whereby TRMT6‐mediated mRNA m^1^A modification up‐regulates CDK9 expression.

CDK9 interact with cyclin T1 and phosphorylate RNA Pol II at Ser2, which increases the transcription of its downstream oncogenic effectors such as MCL1 and BCL‐2 [47]. Besides, CDK9 can also interact with STAT3 and up‐regulate the levels of p‐STAT3 to facilitate the proliferation of lung cancer cells [36]. In fact, p‐STAT3, MCL1 and BCL‐2 have also been recognized to play critical roles in HCC [33, 34, 35]. In this study, we proved that these molecules were significantly elevated upon TRMT6 overexpression and could be reversed by CDK9 inhibitor FIT‐039. However, there are still some unknown downstream effectors of CDK9 that may be involved in the occurrence and progression of HCC. It is worth mentioning that FIT‐039, a highly selective CDK9 inhibitor with oral activity, binds to the ATP‐binding pocket, thereby inhibiting the transcription of multiple DNA viruses [48] including HBV [30] and the progression of virus‐related tumors, such as Kaposi's sarcoma‐associated herpes virus (KSHV)‐positive primary effusion lymphoma [49]. Given the results of preclinical toxicity tests showing that an overdose of FIT‐039 had no impact on body weight of mice and their biological markers [48], we expect its clinical application in the treatment of HCC, especially HBV‐related HCC.

While CDK9 inhibition impairs HBV replication, the mechanistic basis remains poorly defined. Among CDK9‐binding partners identified by LC‐MS/MS, TARDBP was not the most up‐regulated. However, this DNA/RNA‐binding protein was initially found to repress HIV LTR transcription [50] and was later shown to act as a transcriptional activator that promotes TNF‐α expression [51] and HBV replication [39]. We therefore prioritized TARDBP as a candidate CDK9 substrate for further investigation. Our data showed that TARDBP knockdown caused a significant reduction in pgRNA levels, further supporting the role of TARDBP in HBV replication. A previous study has suggested that the functional switch of TARDBP between an activator and a repressor may be regulated by post‐translational modifications or interactions with different factors [39], we thus speculate that TARDBP can be phosphorylated by CDK9, and this modification is essential for its activator function. To validate this, we performed Co‐IP combined with LC‐MS/MS in FIT‐039‐treated HCC cells and control cells, and demonstrated that the phosphorylation levels at Ser254, Ser180/Ser183, Ser163, Thr141, and Thr88 were regulated by CDK9. A previous study showed that deleting RRM1 of TARDBP only slightly reduced the interaction with HBV CP, while deletion of RRM2 completely abrogated their interaction [39], suggesting that RRM2 is required for the interaction between TARDBP and HBV CP. Among the above phosphorylation sites, Ser254 was just localized in RRM2 [52] and the function of the phosphorylation at this site is largely unclear. Using the dual‐luciferase reporter system and ChIP‐qPCR assays, we demonstrate that CDK9‐mediated phosphorylation of TARDBP at Ser254 promotes pgRNA transcription by enhancing TARDBP binding to HBV CP.

Exporting non‐canonical viral transcripts which is coupled with splicing is one of the major challenges during HBV RNA processing [39]. Exporting pgRNA from nuclear requires specific proteins to facilitated this process. Considering that TARDBP is a potential pgRNA‐interacting protein [53] and the double mutant of D105A and S254A displays a significant loss in binding affinity with RNA [41], we thus speculate that the phosphorylation at Ser254 will facilitate the interaction of TARDBP with pgRNA. Given that TARDBP interacts with proteins involve in RNA metabolism, such as HNRNPC, HNRNPK, and HSP90, and can also inhibit pgRNA splicing [39], we suppose that phosphorylation at Ser254 may decrease the splicing activity of pgRNA, which was supported by our data. However, the precise mechanism has not been delineated.

In summary, our findings highlight the dual role of TRMT6‐mediated m^1^A modification on *CDK9* mRNA in HCC progression and HBV replication, providing the potential targets for the treatment of HCC, especially HBV‐related HCC. Given that multiple small‐molecule inhibitors of CDK9 have been developed and some are in clinical trials, thus targeting CDK9 will be a promising therapeutic strategy for HBV‐related HCC.

## 4 Experimental Section

### 4.1 Human HCC Specimens

With the institutional review board approval (No. XJTU1AF2025LSYY‐312 and No. XJTU1AF2026LSYY‐0112) and a waiver of informed consent, HCC tissues and adjacent non‐cancerous liver tissues (control subjects) used in this study were obtained from The First affiliated Hospital of Xi'an Jiaotong University. All experiments were conducted in accordance with both the Declarations of Helsinki and Istanbul. The information of all patients was presented in Tables S7–S11.

### 4.2 Preparation of snRNA‐seq Library

Tissues were harvested and washed in pre‐cooled PBSE (PBS buffer containing 2 mM EGTA). Nuclei isolation was carried out using GEXSCOPE Nucleus Separation Solution (1185011, Singleron Biotechnologies, Nanjing, China) refer to the manufacturer's product manual. Isolated nuclei were resuspended in PBSE to 10^6^ / 400 µl, filtered through a 40 µm cell strainer, and counted with Trypan blue. Nuclei enriched in PBSE were stained with DAPI (1:1,000) (D1306, Thermo Fisher, MA, USA). Nuclei were defined as DAPI‐positive singlets. The concentration of single nucleus suspension was adjusted to 3‐4 × 10^5^ nuclei/mL in PBS. Single nucleus suspension was then loaded onto a microfluidic chip and the libraries were constructed according to the protocol of FocuSCOPE Single Cell mRNA × HBV Library Kit (Singleron Biotechnologies, Nanjing, China). The resulting snRNA‐seq libraries were sequenced on the Illumina NovaSeq 6000 platform (Illumina, San Diego, CA) with 150 bp paired‐end reads.

### 4.3 Process Data of snRNA‐seq

Raw reads were processed to generate gene expression profiles using CeleScope v1.12.0 (Singleron Biotechnologies) with default parameters. After pro‐processing, R2 reads were aligned against the hg38 transcriptome using STAR v2.6.1b [54]. Uniquely mapped reads were then assigned to exons with FeatureCounts (v2.0.1) [55]. Successfully assigned reads with the same cell barcode, UMI and gene were grouped together to generate the gene expression matrix for further analysis. HBV‐enriched libraries were analyzed using CeleScope v 1.12.0 (Singleron Biotechnologies). After pro‐processing, R2 reads were aligned against the HBV genome using STAR v2.6.1b with outFilterMatchNmin set to 80. After obtaining the BAM file, in order to remove ambient contamination, we adopted a two‐step filtration process. First, a UMI needs to be supported by a certain number of reads. Second, an HBV‐positive cell needs to be supported by a certain number of UMIs. Thresholds for supporting reads and UMIs were determined by the Otsu's method [56].

### 4.4 Doublet Removal and Data Integration

Potential doublets were identified using DoubletFinder with default parameters. A doublet score was calculated for each cell, and a threshold based on a bimodal distribution was applied. The expected doublet rate was set to 5%. Cells predicted as doublets or with a doublet score exceeding 0.25 were filtered out. To integrate cells from different individuals into a shared space for downstream analysis, batch effect correction was performed using the Harmony algorithm, regressing out variation associated with sample origin (“orig.ident”). This ensured consistent clustering and enhanced cross‐sample comparability.

### 4.5 Clustering and Cell Type Identification

Clustering followed the Seurat v4.0 integration workflow [57]. Variable genes were identified using the “FindVariableFeatures” function, and the top 2 000 variable genes were selected. Principal component analysis (PCA) was performed using the first 20 principal components. The “RunHarmony” function was applied to the PCA embeddings to remove batch effects. Clustering was performed using the “FindClusters” function (operating on a k‐nearest neighbor graph) with a resolution parameter of 1.0. Results were visualized in UMAP and t‐distributed stochastic neighbor embedding (t‐SNE) plots. To identify differentially expressed genes (DEGs), the “FindMarkers” function in Seurat (based on a Wilcoxon rank‐sum test) was applied with default parameters. DEGs were defined as genes expressed in over 25% of cells within a cluster and exhibiting an average log2 (fold change) greater than 2 compared to all other clusters. Cell type annotation for each cluster was determined by integrating the expression of canonical markers identified among the DEGs with established literature knowledge. Marker gene expression was visualized using Seurat's “FeaturePlot” and “VlnPlot” functions. The Seurat clustering workflow was re‐run within each major cell type to identify potential subtypes.

### 4.6 Gene Set Score Analysis

Module scores representing specific gene expression programs were calculated for single cells using the “AddModuleScore” function in Seurat. Briefly, all analyzed genes were binned based on their average expression across the dataset. Control genes were randomly selected from each bin. The module score for a given gene set was computed as the average expression level of those genes in a single cell, minus the average expression level of the assigned control gene set from the same expression bins.

### 4.7 Cell Lines

HCC cell lines MHCC97H (201128A, RRID: CVCL_4972, Zhejiang, China) and Huh7 (SCSP‐526, RRID: CVCL_0336, Shanghai, China) were purchased from Zhejiang Meisen Cell Technology Co., Ltd. and National Collection of Authenticated Cell Cultures (NCACC), respectively. HepG2.2.15 cell line (200912F, RRID: CVCL_L855, Zhejiang, China) was purchased from Zhejiang Meisen Cell Technology Co., Ltd., which is an ideal cell model for studying HBV replication in vitro. HepAD38 cells (CRL‐3561, RRID: CVCL_M177, Zhejiang, China) were generously provided by Dr. Chao Fan (Department of Infectious Disease, Tangdu Hospital, the Fourth Military Medical University). All cell lines were cultured in DMEM medium (G4511, Servicebio, Wuhan, China) supplemented with 10% fetal bovine serum (26140079, Gibco, USA) at 37°C ‐in a humid incubator (Panasonic, Osaka, Japan) in 5% CO_2_. In addition, HepAD38 cells were maintained in DMEM medium supplemented with 0.3 µg/mL tetracycline (T0912L, TargetMol, MA, USA). To establish HepG2‑NTCP cells, lentiviruses expressing NTCP were produced in HEK293T cells transfected with pLV3‐CMV‐SLC10A1‐3×FLAG‐CopGFP‐Puro plasmids (P45509, Miaoling Bio, Wuhan, China). HepG2 cells (SCSP‐510, RRID: CVCL_0027, Shanghai, China) were then infected with these viruses and subjected to puromycin selection for 7 days. All procedures on HepG2.2.15 and HepAD38 cells were performed in a biosafety level 2 laboratory (P2 laboratory).

### 4.8 RNA Isolation and Quantitative Reverse Transcription PCR (qRT‐PCR)

The procedures of qRT‐PCR were similarly performed as described previously [58]. The mRNA levels of the candidate genes were normalized to *β‐actin* or *18S* rRNA. The primer sequences were presented in Table S12.

### 4.9 Western Blotting Analysis

The protocol was described in a previous study [59], and the antibodies used were presented in Table S13.

### 4.10 Dot‐Blot Assay

Total RNAs were isolated by Trizol (15596026CN, Invitrogen, CA, USA) reagent and purified by chloroform/isoprop anol precipitation. The purified mRNAs were denatured, and 400 ng mRNA was dropped onto the positively charged nylon membrane (FFN13, Beyotime, Shanghai, China) directly followed by air‐drying for 10 min and crosslinked to membrane in the Stratalinker 2400 UV Crosslinker (LUYOR, Shanghai, China) twice at 1200 microjoules for 20 s. Next, the membrane was washed in RNase‐free wash buffer for 5 min to wash off unbound mRNAs, followed by incubation in BSA (9048‐46‐8, Amresco, USA) for 1 h. The membrane was then incubated in anti‐m^1^A antibody (1:1000, ab208196, Abcam, Cambridge, UK) dilution overnight at 4°C and washed four times for 4 min each in washing buffer followed by incubating the membrane with HRP‐conjugated rabbit IgG (1:2500, ZB‐2301, ZSGB‐Bio, Beijing, China) secondary antibody for 1.5 h. Then, immunoblotting signals were harvested immediately by Tanon 5200. Meanwhile, 0.02% methylene blue (457250, Sigma‐Aldrich, MO, USA) staining was used to determine the amount of mRNA spotted on the membrane.

### 4.11 DNA Isolation

Cells were collected and then washed with PBS, followed by adding 1% SDS‐PK buffer (300 µL) and 20% PK (3 µL, 39450‐01‐6, Merck, Darmstadt, Germany) and incubating at 48°C overnight. Next day, cells were supplemented with DNA extraction solution (700 µL, P1012, Solarbio, Beijing, China) and centrifuged at 12500 rpm for 15 min at 4°C. The supernatant was mixed with ammonium acetate (750 µL, 39450‐01‐6, Merck, Darmstadt, Germany) solution and stood at ‐20°C overnight. After centrifugation at 12 500 rpm for 45 min, the precipitation was supplemented with 75% methanol (1 mL, 67‐56‐1, Fuyu Chemical, Tianjin, China) and centrifuged at 13500 rpm for 10 min, and the precipitation was air‐dried and diluted in sterile water.

### 4.12 Detection of HBV cccDNA

Cell‐derived genomic DNA was incubated with Plasmid‐Safe ATP‐dependent DNase (PSAD) (E3101K, Biosearch technologies, UK) for 30 min at 37°C, following incubation at 70°C for 30 min to get circular duplex DNA. DNA samples were mixed with the primers, TaqMan probes, and Premix Ex Taq (RR390A, Takara, Japan), and the levels of HBV cccDNA were then quantified by qPCR. *β‐actin* served as a reference gene. The sequences of primers and TaqMan probes were listed in Table S14.

### 4.13 Detection of HBV DNA

Cellular supernatant was collected and incubated with 10% PEG8000 (P5413, Sigma‐Aldrich, MO, USA) for 5 h on the ice with gentle shaking, followed by centrifugation at 8000 rpm for 25 min at 4°C. HBV particles condensed in the bottom of tube were then resuspended in the DMEM medium without FBS. HBV particles were cleaved by Sample Release Agent (S1011, Sansure Biotech Inc, Hunan, China) and HBV DNA was then measured based on the manufacturer's instructions of Hepatitis B Virus Nucleic Acid Assay Kit (Sansure Biotech Inc, Hunan, China).

### 4.14 siRNA Transfection

siRNAs (RiboBio, Guangzhou, China) and 1.2‐fold volume X‐treme GENE siRNA Transfection Reagent (61506000, Roche Diagnosis, Basel, Switzerland) were separately diluted in Opti‐MEM (50 µL, 31985070, Invitrogen, CA, USA) and stood for 5 min. The X‐treme dilution were then mixed into siRNAs dilutions. After standing for 15 min, the solution was then added into cells with fresh cell culture medium (400 µL) slightly. Cells were incubated for 4‐6 h, following by changing fresh culture medium. The siRNA sequences were listed in Table S15.

### 4.15 Plasmid Transfection

Plasmids (1 µg, Miaoling Bio, wuhan, China) were diluted in Opti‐MEM medium (200 µL) followed by standing for 5 min, and X‐tremeGENE HP DNA Transfection Reagent (2.5 µL, 41106502, Roche, Basel, Switzerland) was then mixed into the Opti‐MEM and incubated for a duration of 15 min. The mixture was supplemented into plates with fresh cell culture medium (800 µL), and changed fresh culture medium after incubating 4‐6 h.

### 4.16 Establishment of Target Genes‐Knockdown, ‐Knockout, and ‐Overexpressing Cells

Knockdown or overexpression of target genes was achieved by transfecting lentivirus purchased from Genechem (Shanghai, China). Knockout of target genes was accomplished with using lentiviral‐based CRISPR‐Cas9 system. Cells were incubated with lentivirus at optimal MOI value for 12 h and cultured in fresh medium for three days. Then, above cells were selected by puromycin (2 µg/mL, P8230, Solarbio, Beijing, China). The sequences of shRNAs and sgRNAs were listed in Table S16, and off‐target effect and efficiency of sgTARDBP were summarized in Table S17 [60].

### 4.17 Co‐Immunoprecipitation (Co‐IP)

Cellular samples were rinsed with PBS‐MG132 (M8699, Sigma‐Aldrich, MO, USA) buffer and lysed with protein lysis buffer (P0013B, Beyotime, Shanghai, China) fortified with phosphatase inhibitor and PMSF for 20 min on ice. Subsequent to centrifugation at 12000 rpm for 20 min, the supernatant was divided into three tubes on average and subjected to overnight antibodies incubation at 4°C. The antigen‐antibody complex was supplemented with Protein A/G Plus Agarose beads (sc‐2003, Santa Cruz Biotechnology, TX, USA) for 2 h. The antigen‐antibody‐beads complex was then collected, and the proteins were isolated and measured by western blotting analysis.

### 4.18 Cell Viability Assay

Cells with different treatments were cultured in 96‐well plates (1000/well) and incubated. Cell viability were evaluated by MTT (M5655, Sigma‐Aldrich, MO, USA) assay and the half maximal inhibitory concentration (IC_50_) was calculated as described previously [59].

### 4.19 Colony Formation Assay

Cells with different treatments were cultured in 6‐well plates (2000/well) for 12 days. Cell colonies underwent fixation using methanol for 20 min, followed by staining with crystal violet (HY‐B0324A, MCE, NJ, USA) for 8 min, and counted by Image J.

### 4.20 Cell Migration/Invasion Assay

Cells were collected and resuspended with DMEM with 0.5% FBS. Then, cells were countered and adjusted to 1×10^4^ cells /150 µL. Next, these cells (150 µL) were added into the upper layer, and 1 mL DMEM with 20% FBS was added into the lower layer of transwell chambers. After incubating for 48 to 72 h, transwell chambers (Corning, NY, USA) were washed with PBS and cells inside upper chambers were removed by cotton swabs, and transwell chambers were then fixed with methanol and air‐dried at room temperature. After staining with crystal violet, transwell chambers were washed with sterile water. The matrigel (354230, Corning, NY, USA) was diluted and hydrated inside the transwell chambers to measure cell invasion ability. Cell migration/invasion abilities were evaluated by cell numbers outside the chambers.

### 4.21 Cell Apoptosis Assay

Cells and culture medium were collected by centrifugation at 800 rpm for 5 min. Next, cells were rinsed with PBS and resuspended in 100 µL binding buffer. Then, Annexin‐V‐FITC and PI were added to the above buffer based on instructions of apoptosis kit (FXP018, 4A BIOTECH, Suzhou, China). Apoptotic cells were analyzed by flow cytometry.

### 4.22 Methylated RNA Immunoprecipitation‐qPCR (MeRIP‐qPCR)

Cells were cultured in 10 cm‐plates and subjected to different treatments. The procedures of RNA fragmentation and m^1^A‐RIP were performed as described previously [22]. In brief, 40 µg poly (A) RNA were diluted in 72 µL RNA‐free water and fragmented with magnesium RNA fragmented buffer (6150S, NEB, MA, USA). Then, RNA was condensed by sodium acetate (S889, Sigma‐Aldrich, MO, USA), liner acrylamide (10408ES03, Yeasen, Shanghai, China) and 100% ethanol, followed by standing at80°C for 30 min. Following a 25‐min centrifugation at 14000 rpm, RNA precipitation was washed with 300 µL 70% ethanol, subsequently undergoing another round of centrifugation at 14000 rpm for 5 min following by air‐dried at room temperature. Next, the condensed RNA was dissolved with 105 µL RNA‐free water. Five µL RNA was packed as input and the rest RNA was incubated with anti‐m^1^A antibody (2 µg, ab208196, Abcam, Cambridge, UK) in IPP buffer supplemented with RNasin RNase inhibitors (N261A, Promega, WI, USA) overnight at 4°C. Forty µL Protein A/G Ultralink Resin (53132, Pierce, IL, USA) were prewashed twice by 1 × IPP buffer containing BSA (0.5 mg/mL, 9048‐46‐8, Sigma‐Aldrich, MO, USA) and mixed with the RNA‐ m^1^A antibody complex at 4°C for 2 h. The mixture was washed with IPP buffer and then mixed with N^1^‐methyladenosine (PR3032, Berry Associates, IN, USA) for 1 h. Subsequently, RNA was separated with phenol‐chloroform precipitation, washed with 70% ethanol, and reverse transcribed by SuperScript III Reverse Transcriptase (18080044, Invitrogen, CA, USA). m^1^A enrichment of each sample was further measured by qPCR. Input served as the normalization control. The primers used for MeRIP‐qPCR are listed in Table S18.

### 4.23 mRNA Stability Assay

Cells were subjected to different treatments. After culturing for 48 h, cells were supplemented with actinomycin D (ACTD) (10 µg/mL, HY‐17559, MCE, NJ, USA) and sampled at different time points. Total RNA was isolated and mRNA levels of *CDK9* at different time points were then measured by qRT‐PCR, with *18S* rRNA serving as the reference gene.

### 4.24 Dual‐Luciferase Reporter Assay

Five plasmids were constructed base on different vectors: (1) a PCI‐neo vector inserting an ATG start codon, a 60 bp sequence containing the m^1^A site of *CDK9*, and firefly luciferase; (2) a point‐mutant variant of (1) with “GTTCGA”replaced by “ GTTCGT”; (3) a negative control plasmid; (4) the pRL‐TK vector for Renilla luciferase expression; (5) a pGL3‐basic vector containing HBV core promoter. These plasmids were transfected into cells as instructions of standard protocols. Cells undergoing different treatment were cultured and transfected with above plasmids for 48 h. Luciferase intensity was evaluated by the Dual‐Glo luciferase Assay System (E2920, Promega, WI, USA).

### 4.25 Polysome Profiling

Cells were subjected to different treatment. Upon reaching 80% confluence, cells were collected in PBS‐ cycloheximide (CHX) (S7418, Selleck, TX, USA) solution, following by centrifugation at 200 g for 5 min. Cells were suspended with 450 µL lysis buffer supplemented with CHX, DTT (W610344, Energy Chmical, Shanghai, China), proteasome inhibitor (P003, NCM Biotech, Suzhou, China) and RNase inhibitor (AM2694, Thermo Fisher, MA, USA), followed by a 5‐min incubation on ice. Subsequently, 10% Triton X‐100 (X100, Sigma‐Aldrich, MO, USA) and 10% sodium deoxycholate (D6750, Sigma‐Aldrich, MO, USA) were added and the mixture was incubated on ice for 5 min. After centrifugation at 16000 g at 4°C, a tenth of cell lysis was packed as input and the rest cell lysis was used for the further experiment.

A sucrose gradient was prepared by filtering 10%, 15%, 20%, 25%, 30%, 35%, 40%, 45% and 50% sucrose solution supplemented with CHX, DTT, proteasome inhibitor and RNase inhibitor in the bottom of ultracentrifugal tube successively, and equal‐OD cell lysis was added on the top of it. The sucrose gradient was ultracentrifuged at 222228 g for 2 h at 4°C. Sucrose gradient was packed each 800 µL and the OD_260nm_ value was measured. RNA in each gradient was isolated with chloroform‐isopropyl alcohol/glycogen‐70% ethyl alcohol. The mRNA levels of *CDK9* in each gradient were determined by qRT‐PCR.

### 4.26 Chromatin Immunoprecipitation (ChIP)

The detailed procedure of ChIP was performed based on the instructions of SimpleChIP Enzymatic Chromatin IP Kit (Magnetic Beads) (9003, Cell Signal Technology, MA, USA). The purified DNA from different groups was subjected to qPCR. The primers used for ChIP‐qPCR are listed in Table S19.

### 4.27 In Vitro kinase Activity Assay

Kinase reactions were performed in a 30 µL volume containing 25 mM Tris‐HCl (pH 7.5, V900312, Merck, Germany), 10 mM MgCl_2_ (AM9530, ThermoFisher, MA, USA), 1 mM MnCl_2_ (244598, Merck, Germany), 1 mM DTT (W610344, Energy Chemical, Shanghai, China), 100 µM ATP (51963‐61‐2, Macklin, Shanghai, China), 200 ng recombinant human CDK9 & cyclin T1 proteins (C40‐18CG, Sino Biological, Beijing, China) and 30 µg TARDBP S254 wild‐type or S254A mutant peptide substrate (GL Biochem, shanghai, China). After incubation at 30°C for 60 min, phosphorylation at TARDBP Ser254 was analyzed by LC‐MS/MS. Peptide sequences were presented in Table S20.

### 4.28 Tumorigenicity Assay in Nude Mice

Four‐week‐old BALB/c nude mice, obtained from Huachuang Sino Pharmatech Co., Ltd were bred under specific pathogen‐free (SPF) condition. 5 × 10^5^ TRMT6‐knockdown MHCC97H cells or their control cells per 150 µL PBS were subcutaneously injected into the right flank of mice. Tumor sizes were measured every four days, and tumor volumes were calculated by the formula: (length × width^2^)/2. At the end of experiments, mice were humanely euthanized and tumors were excised. Two‐way ANOVA was employed to statistically assess tumor dimensions and weights.

In in vivo rescue experiment, 5 × 10^5^ TRMT6‐overexpresing MHCC97H cells or their control cells per 150 µL PBS were subcutaneously injected into the right flank of mice. When the volumes of TRMT6‐overexpressing cell‐derived tumors reached 80‐90 mm^3^, mice were categorized into three distinct groups. One group did not receive any treatment as control, and the other two groups was administrated with 150 mg/kg FIT‐039 (HY‐18944, MCE, NJ, USA) or the equal dose of DMSO once every two days. At the end of experiments, mice were humanely euthanized and tumors were excised. Two‐way ANOVA was employed to statistically assess tumor dimensions and weights. The animal studies were approved by the Animal Ethics Committee of Xi'an Jiaotong University (No. XJTUAE‐2024‐2524), and all animals received human care during the experiment.

### 4.29 Tumorigenicity Assay in Patient‐Derived Xenograft (PDX) Model

Six‐week‐old female NCG mice (NOD/ShiLtJGpt‐Prkdcem26Cd52Il2rgem26Cd22/Gpt) were purchased from GemPharmatech Co., Ltd and housed in SPF environment. All mice had sterile food and water throughout the experiment. Frozen human HCC tissues were reactivated, and non‐cancerous and necrotic tissues were removed before being cut into 1–2 mm^3^ fragments for implantation. Five NCG mice were anesthetized, and tumor fragments were implanted subcutaneously into the area of back. Tumor volumes were measured every 3 days, and calculated as the formula: (length × width^2^)/2. Once tumors reached 900–1000 mm^3^, mice were euthanized, and tumor fragments were then harvested and re‐implanted into 20 new NCG mice.

Six days after tumor implantation, NCG mice were randomly divided into four groups. Mice in two groups received intratumoral injection of AAV‐TRMT6 (1.0 × 10^1^ ^2^ vg/mL), while the remaining two groups were administered AAV‐NC (1.0 × 10^1^ ^2^ vg/mL) every 3 days for a total of 7 injections. Mice were then treated with 150 mg/kg FIT‐039 or an equivalent volume of DMSO by subcutaneous injection every 2 days for 6 doses. Tumor size was monitored at each injection, and tumor volume was calculated as the formula: (length × width^2^)/2. At the endpoint, mice were euthanized and tumors were harvested. Tumor volumes and weights were analyzed by two‐way ANOVA. All animal procedures were approved by the Institutional Review Board (No. XJTU1AF2026LSYY‐0112) and the Animal Ethics Committee of Xi'an Jiaotong University (No. XJTUAE‐2025‐3830) and performed in accordance with institutional guidelines for humane animal care.

### 4.30 Immunohistochemistry (IHC) and Hematoxylin ‐Eosin (H&E) Staining

The protocols for IHC and H&E staining in paraffin‐embedded sections and xenograft tumors were described previously [61]. The sections were scanned by Pannoramic SCAN (3D HISTECH, Budapest, Hungary).

### 4.31 Statistical Analysis

Data analysis was conducted using SPSS and Graphpad Prism 8.0. Student's *t*‐test, and Mann‐Whitney *U* test was employed to compare two variables, and ANOVA was used to compare three or more. Each data set was analyzed separately. Data was expressed as mean ± standard deviation (SD). A *p*‐value < 0.05 was considered statistically significant.

## Author Contributions

P.H. initiated and supervised the study. P.H., R.Z., and S.W.D. contributed to conception and study design. R.Z., D.D.Z., Y.B.W., Q.Q.G., W.F.Z., M.M.Y., S.M.W., and D.X.L. conducted experiments. P.H. and R.L. provided animals, tissue samples, and facilities, etc. R.Z., D.D.Z., Y.Y., and R.R.C. analyzed and interpreted data. P.H., R.Z., and D.D.Z. wrote and revised the manuscript. All authors reviewed and approved the final manuscript.

## Conflicts of Interest

The authors declare no conflicts of interest.

## Supporting information




**Supporting File 1**: advs75266‐sup‐0001‐SuppMat.docx.

**Supporting File 2**: advs75266‐sup‐0002‐Data.zip.

**Supporting File 3**: advs75266‐sup‐0003‐TableS1.xlsx.

**Supporting File 4**: advs75266‐sup‐0004‐TableS3.xlsx.

**Supporting File 5**: advs75266‐sup‐0005‐TableS4.xlsx.

**Supporting File 6**: advs75266‐sup‐0006‐TableS5.txt.

**Supporting File 7**: advs75266‐sup‐0007‐TableS6.xlsx.

**Supporting File 8**: advs75266‐sup‐0008‐TableS17.csv.

## Data Availability

The data that support the findings of this study are available on request from the corresponding author. The data are not publicly available due to privacy or ethical restrictions.
