# Supplementary material for: TRMT6‐Mediated m1A Modification of CDK9 mRNA is a Dual‐Pronged Pathogenic Driver for HBV‐Related Hepatocellular Carcinoma
Source: Adv Sci (Weinh). 2026 Apr 20;13(39):e14172. doi: 10.1002/advs.202514172 (PMC13334973; doi:10.1002/advs.202514172)
Supplement: Supplementary file 1 — Supporting File 1: advs75266‐sup‐0001‐SuppMat.docx. [file ADVS-13-e14172-s002.docx]

**TRMT6-Mediated m^1^A Modification of *CDK9* mRNA Is a Dual-Pronged Pathogenic Driver for HBV-Related Hepatocellular Carcinoma**

Rui Zhang^1,2,3#^, Dandan Zong^5#^, Rui Liu^4^, Yubo Wang^1,2^, Qingqing Gu^1,2^, Yao Yao^1,2^, Wenfang Zheng^1,2^, Mengmeng Yuan^1,2^, Simeng Wang^1,2^, Rongrong Cui^1,2^,

Daxu Li^6^, Siwen Dang^7 *^, and Peng Hou^1,2 *^

^1^ Department of Endocrinology and Metabolism, The First Affiliated Hospital of Xi’an Jiaotong University, Xi’an 710061, P.R. China.

^2^ International Joint Research Center for Tumor Precision Medicine of Shaanxi Province, The First Affiliated Hospital of Xi’an Jiaotong University, Xi’an 710061, P.R. China.

^3^ Department of Infectious Diseases, Tangdu Hospital, The Fourth Military Medical University, Xi’an 710038, P.R. China.

^4^ Department of Radio-Oncology, The First Affiliated Hospital of Xi’an Jiaotong University, Xi’an 710061, P.R. China.

^5^ Yulin Hospital, the First Affiliated Hospital of Xi’an Jiaotong University, Yulin 719000, P.R. China.

^6^ Department of Stomatology, The First Affiliated Hospital of Xi'an Jiaotong University, Xi’an 710061, P.R. China.

^7^ Department of Endocrinology and Metabolism, The Second Affiliated Hospital, Xi'an Jiaotong University, Xi'an 710061, P.R. China.

^#^These authors contributed equally.

* Corresponding authors:

E-mail (Peng Hou): [phou@xjtu.edu.cn](mailto:phou@xjtu.edu.cn)

E-mail (Siwen Dang): dangsiwen@stu.xjtu.edu.cn

**Supplementary Figures**

**
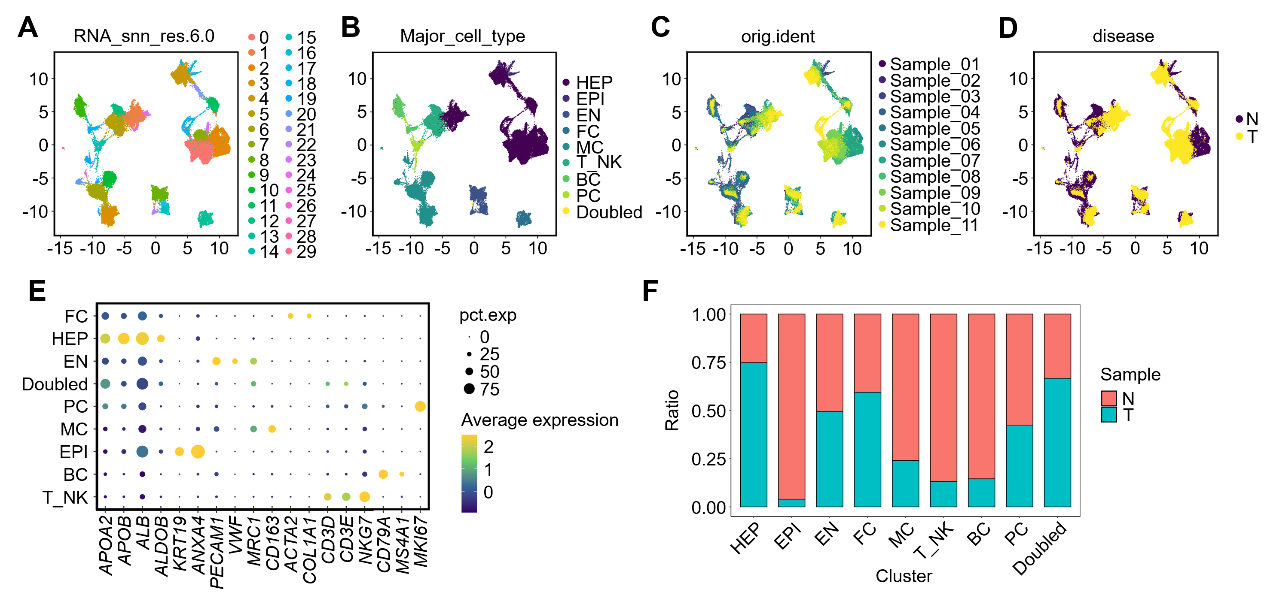
**

**Figure S1.** Cell distribution by single-cell RNA-seq analysis of human HCC tissues and normal controls. A-B) UMAP plots displaying the major cell types in human HCC tissues and normal controls. C-D) UMAP plots displaying the samples, accessions and states of cells in HCC and control tissues. E) The gene expression of specific makers in different cell types. F) The ratio of different cell types in tumor (T) or normal cells (N).

**
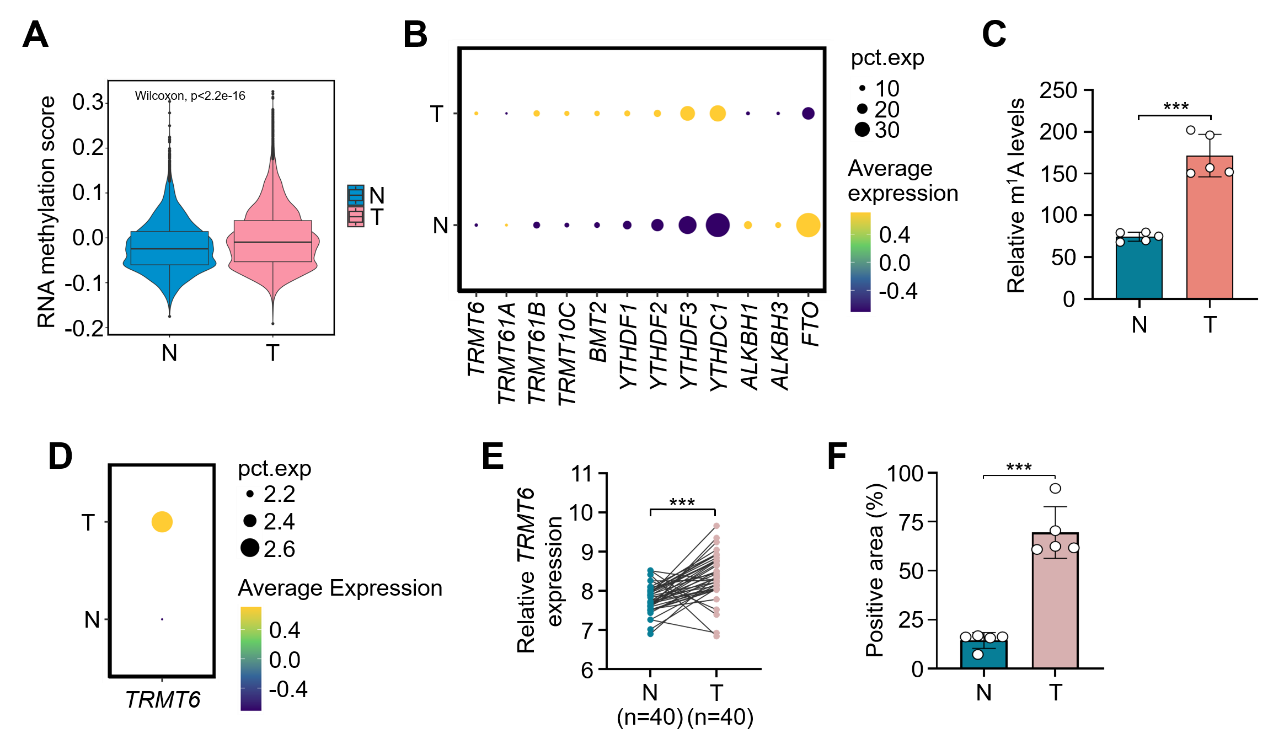
**

**Figure S2.** Increased levels of m^1^A modification and its “writer” TRMT6 in HCCs. A) The gene set scores of RNA methylation-related molecules in normal and tumor cells. B) Gene expression of m^1^A-related gene set in normal and tumor cells. C) The statistically analysis of m^1^A levels in 5 pairs of HCC tissues (T) and their matched non-cancerous tissues (N) determined by dot-blot assays (left panel). D) *TRMT6* expression in normal and tumor cells. E) The relative mRNA expression of *TRMT6* was analyzed in HCC tissues (T) and their matched non-cancerous liver tissues (N) using TCGA database by paired *t*-test. F) The statistically analysis of TRMT6 protein levels in paraffin sections from HCC tissues and control tissues using IHC staining (related to Fig. 1O). The data were shown as the mean ± SD. ***, *P* <0.001.

**
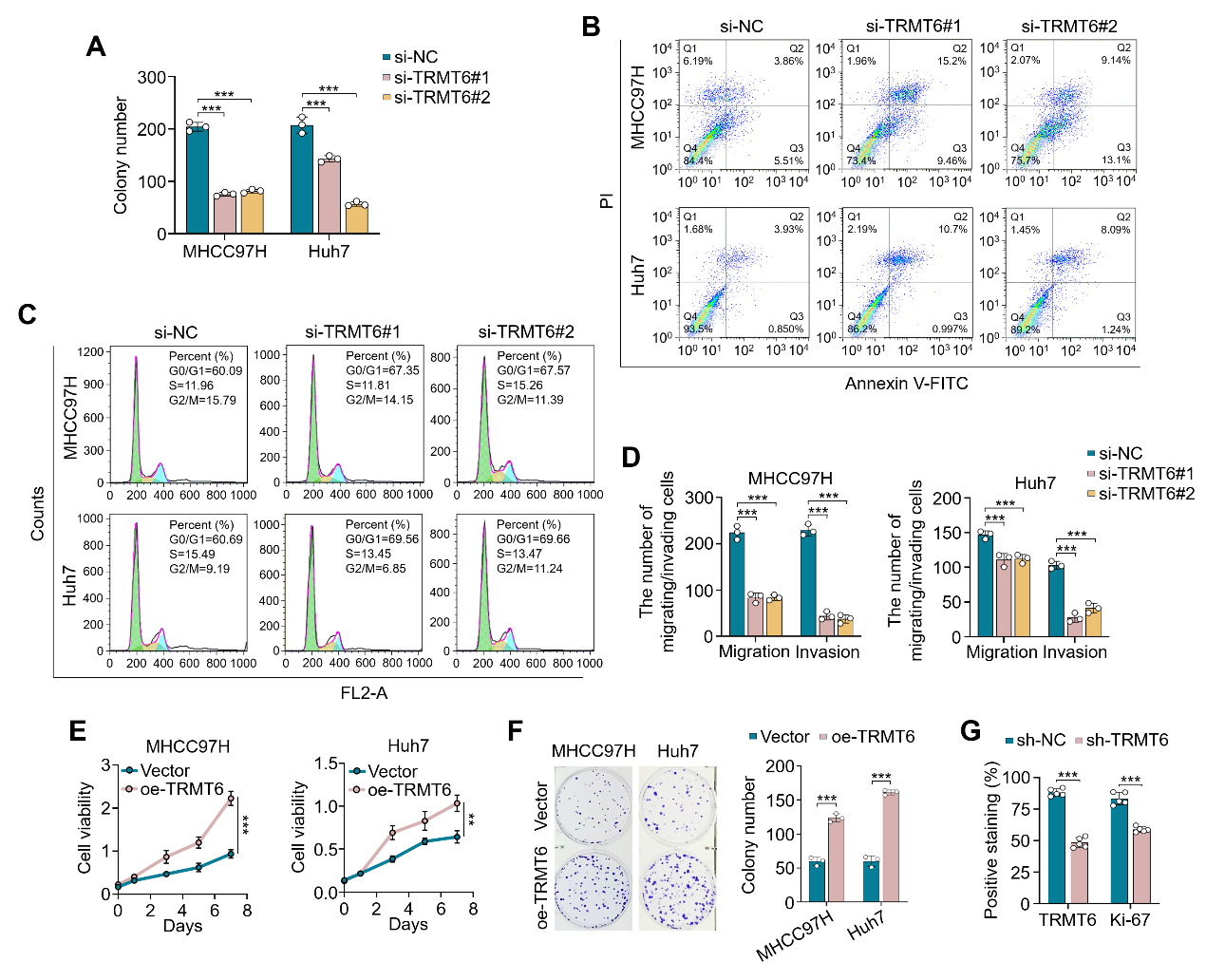
**

**Figure S3.** TRMT6 enhances the malignant behaviors of HCC cells. A) TRMT6 was knocked down in MHCC97H and Huh7 cells, and evaluated its effect on colony formation ability. The statistical analysis was shown in the panel. TRMT6 was knocked down in MHCC97H and Huh7 cells, and cell apoptosis(B) and cell cycle (C) were evaluated by flow cytometry. Shown were representative images of flow cytometry (related to Fig. 2E and Fig. 2F). D) The effect of TRMT6 knockdown on the migration and invasion of MHCC97H and Huh7 cells was evaluated by transwell assays. Shown were the statistical results of migrating and invading cells (related to Fig. 2G). Student’s t‑test was used for statistical analysis. E) TRMT6 was ectopically expressed in MHCC97H and Huh7 cells, and its effect on cell viability was evaluated by MTT. F) The effect of TRMT6 overexpression on colony formation ability of MHCC97H and Huh7 cells. The representative images were shown in the left panel and the statistical analysis was shown in the right panel. G) IHC staining of TRMT6 and Ki-67 in the indicated tumors. The representative images were shown in the left panel, the proportion of positive staining in each image was statistically analyzed in the right panel. Scar bar, 100 μm. The data were shown as the mean ± SD. **, *P* <0.01; ***, *P* <0.001.


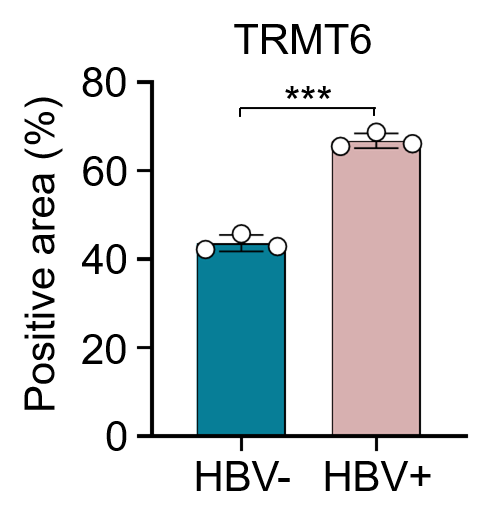


**Figure S4.** The statistically analysis of TRMT6 protein levels in paraffin sections from HBV+ and HBV- HCC tissues using IHC staining (related to Fig. 3E). The data were shown as the mean ± SD. ***, *P* <0.001.


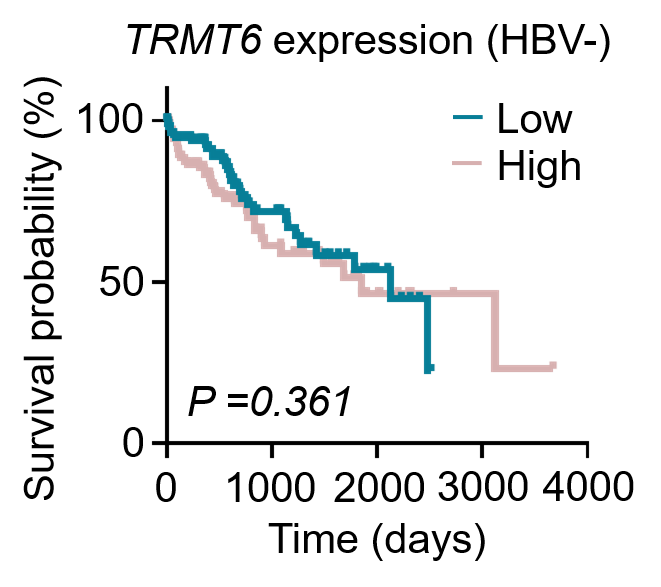


**Figure S5.** Survival curves of HBV- HCC patients with high and low expression of *TRMT6*.

**
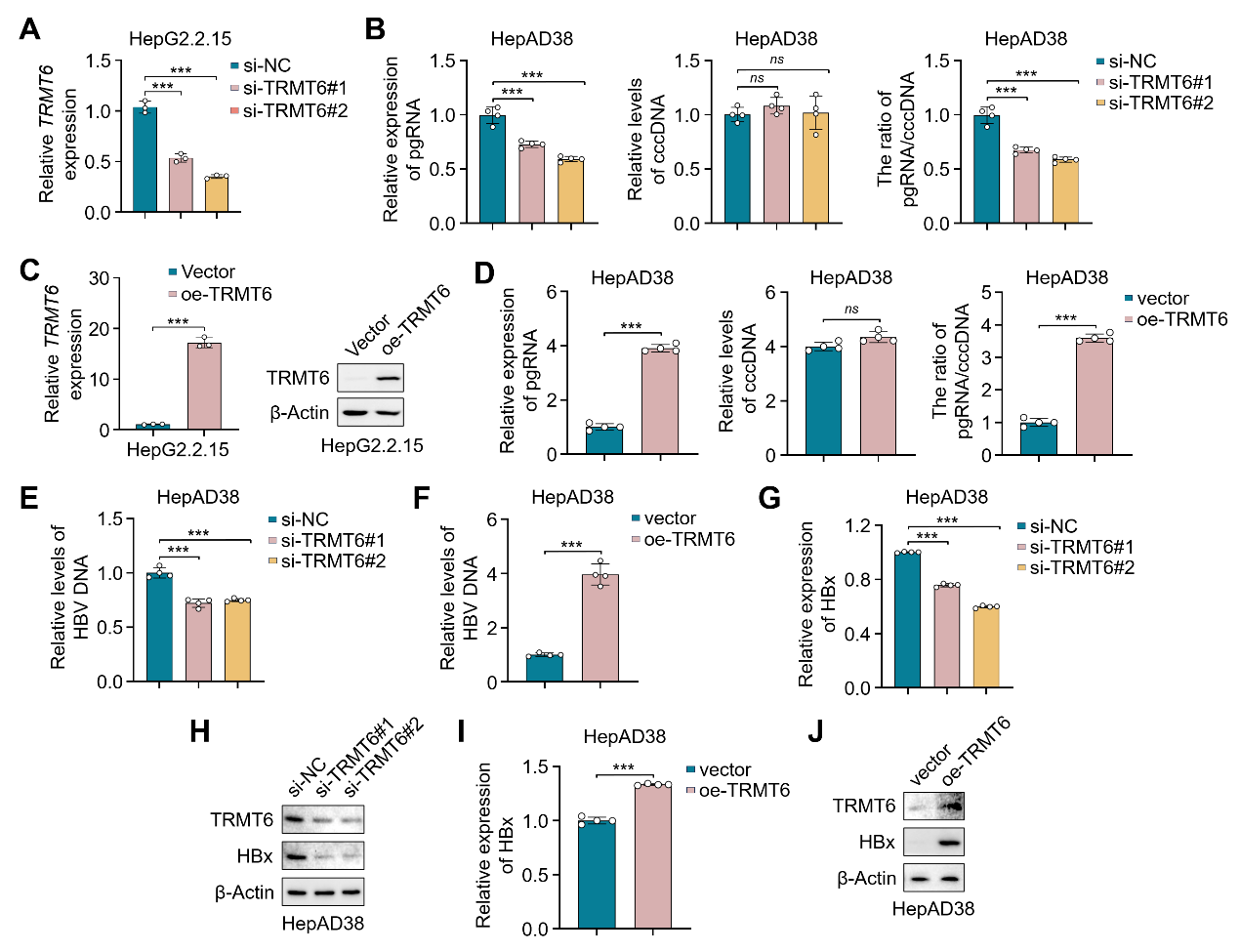
Figure S6.** The effect of TRMT6-mediated m^1^A modification on HBV replication. A) The knockdown of TRMT6 in HepG2.2.15 cells by siRNAs was validated by qRT-PCR, *β-actin* was used as the reference gene of qRT-PCR. B) TRMT6 was knocked down in HepAD38 cells, and the expression of pgRNA was analyzed by qRT-PCR (left panel), the levels of cccDNA were measured by TaqMan-qPCR (middle panel) and the ratio of pgRNA/cccDNA was calculated (right panel). C) The overexpression of TRMT6 by plasmids was validated by western blotting analysis, β-Actin serves as the loading control. D) TRMT6 was overexpressed in HepAD38 cells, and the expression of pgRNA was analyzed by qRT-PCR (left panel), the levels of cccDNA were measured by TaqMan-qPCR (middle panel) and the ratio of pgRNA/cccDNA was calculated (right panel). TRMT6 was knocked down (E) and overexpressed (F) in HepAD38 cells, the levels of HBV DNA in cellular supernatant were then measured using commercial kit. TRMT6 was knocked down in HepAD38 cells, and its effect on mRNA (G) and protein levels (H) of HBx was then evaluated by qRT-PCR (left panel) and western blotting (right panel) assays. The effect of TRMT6 overexpression on mRNA (I) and protein levels (J) of HBx was assessed by qRT-PCR (left panel) and western blotting (right panel) assays in HepAD38 cells. *β-actin* was used as the internal control for qRT-PCR and TaqMan-qPCR, and β-Actin was used as a loading control for western blotting analysis. The data were shown as the mean ± SD. ***, *P* <0.001; *ns*, no significance.

**Figure S7.** TRMT6 shows no regulation effect on MRPL4. TRMT6 was knocked down in MHCC97H and Huh7 cells, and mRNA (A) and protein levels (B) of MRPL4 were measured. *β-actin* was used as the internal control for qRT-PCR, and β-Actin was used as a loading control for western blotting analysis. The data were shown as the mean ± SD. *ns*, no significance.


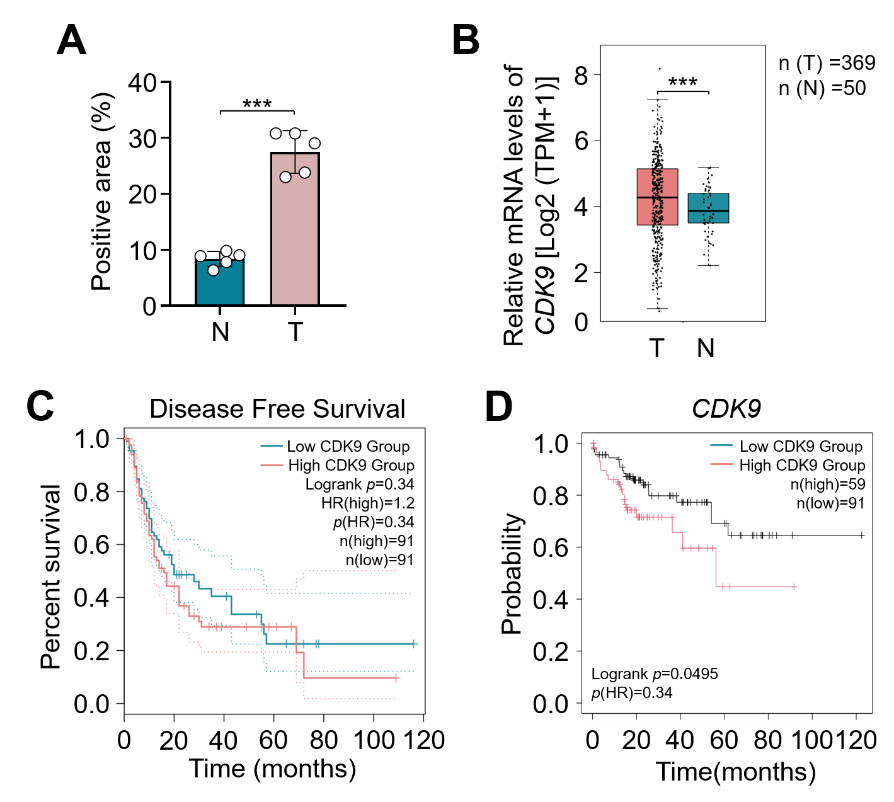


**Figure S8.** High expression of *CDK9* is correlated to poor prognosis of HCC patients. A) The protein levels of CDK9 in HCC sections (T) and controls (N) were statistically analyzed by IHC staining (related to Fig. 4B). B) The relative mRNA expression of *CDK9* was analyzed in HCC tissues (T) and control subjects (N) using TCGA database. C) The disease-free survival curves of HCC patients with low and high expression of *CDK9*. D) Survival curves of HBV+ HCC patients with high and low expression of *CDK9*. The data were shown as the mean ± SD. ***, *P* <0.001.


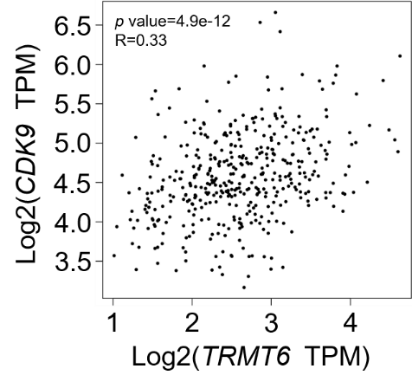


**Figure S9.** The correlation between mRNA levels of *CDK9* and *TRMT6* (data from TCGA database).


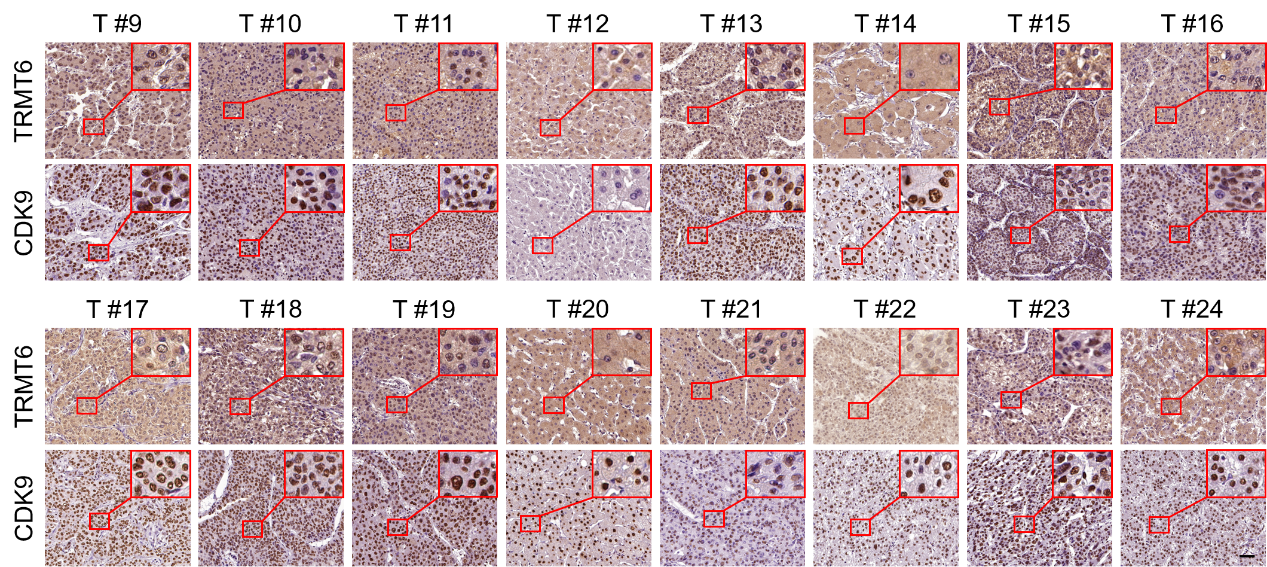


**Figure S10.** Representative IHC staining of TRMT6 and CDK9 in HCC tissues (related to Fig. 4D). Scale bar, 50 μm.


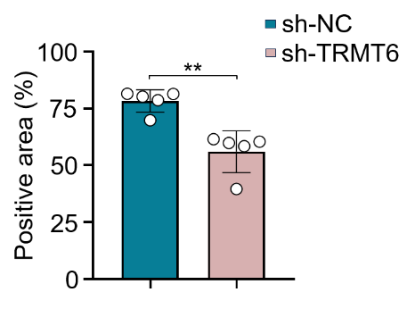


**Figure S11.** The positive area of CDK9 staining in the indicated tumors (related to Fig. 4G). The data were shown as the mean ± SD. **, *P* <0.01.


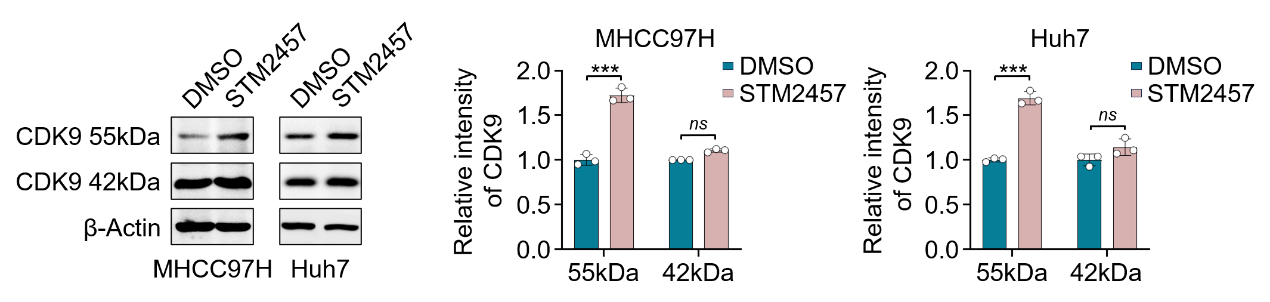


**Figure S12.** MHCC97H and Huh7 cells were treatment with METTL3 inhibitor STM2457, and its effect on CDK9 expression was assessed by western blotting analysis (left panel). β-Actin was used as a loading control. The statistical analysis of western blotting strips from replicate experiments was showed on the middle and right panels. The data were shown as the mean ± SD. ***, *P* <0.001; *ns*, no significance.


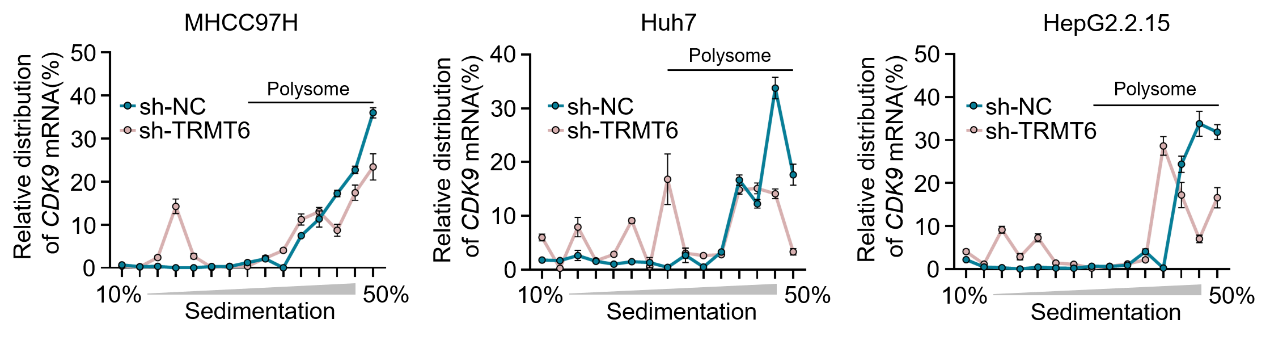


**Figure S13.** TRMT6 enhances the translation efficiency of *CDK9* mRNA. TRMT6 was knocked down in MHCC97H, Huh7 and HepG2.2.15 cells. After 48 h, the equal-OD cell lysis was then subjected to sucrose gradient density centrifugation. The levels of *CDK9* mRNA in all the gradients were determined by qRT-PCR. *β-actin* was used as the internal control. The data were shown as the mean ± SD.

**
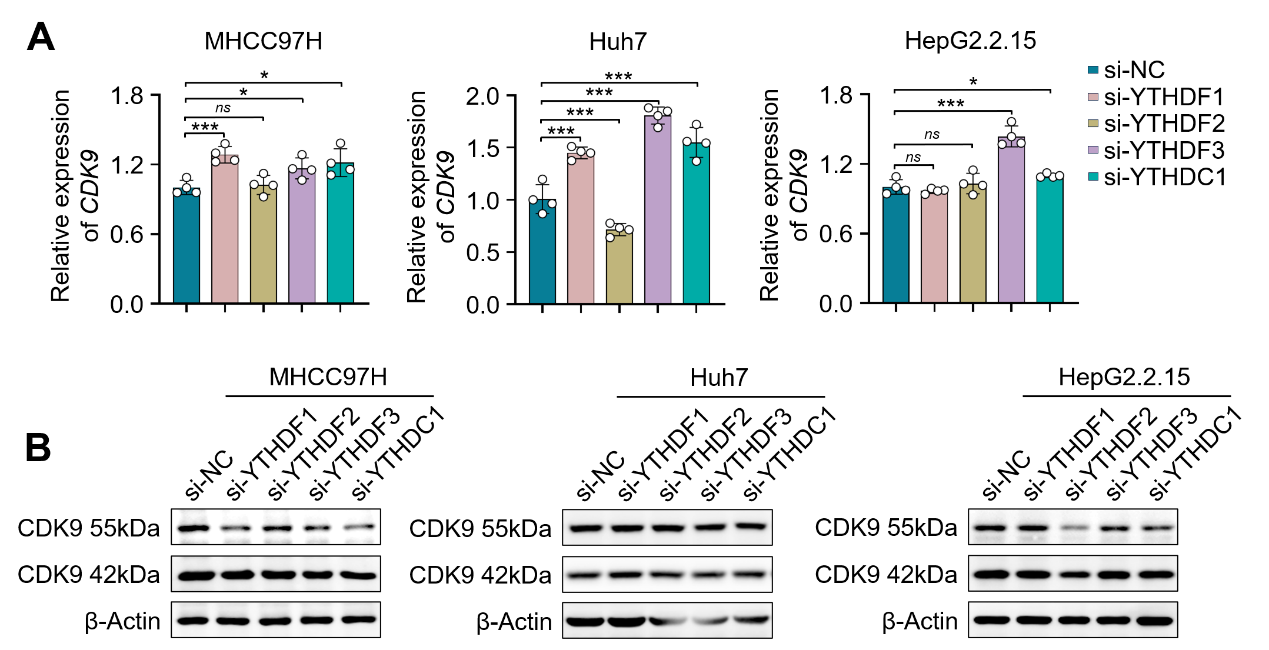
**

**Figure S14.** The screening of m^1^A readers. m^1^A readers (YTHDF1-3 and YTHDC1) were knocked down in MHCC97H, Huh7 and HepG2.2.15 cells. The mRNA (A) and protein (B) levels of CDK9 were assessed by qRT-PCR and western blotting assays. *β-actin* was used as the internal control for qRT-PCR, and β-Actin was used as a loading control for western blotting analysis. The data were shown as the mean ± SD. *, *P* <0.05; ***, *P* <0.001; *ns*, no significance.

**
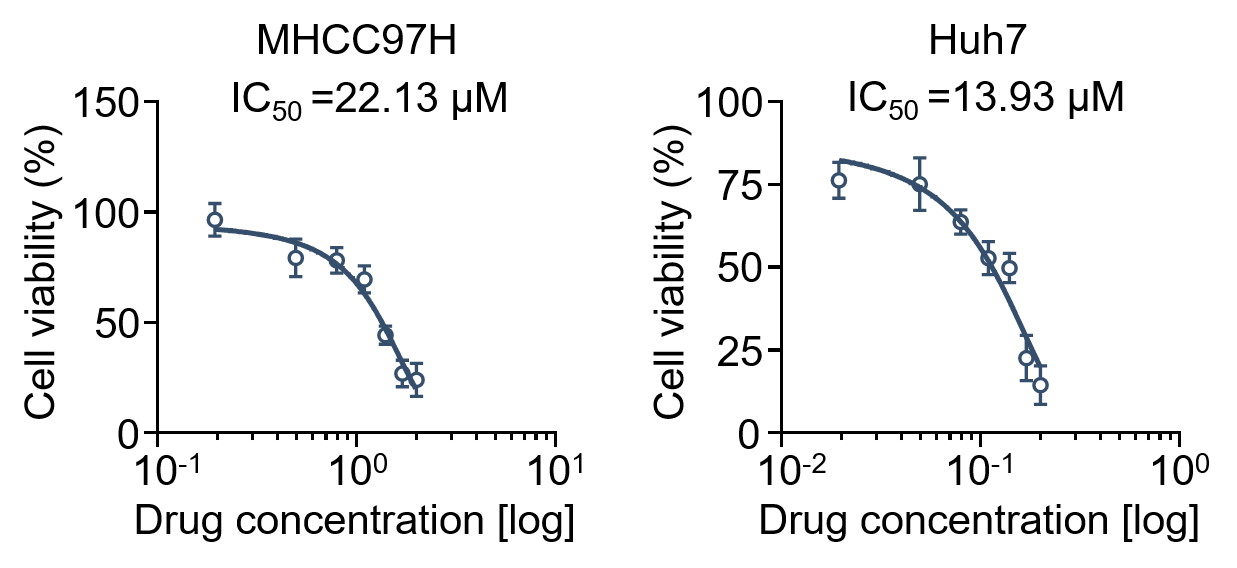
**

**Figure S15.** The IC_50_ assessment of FIT-039. MHCC97H and Huh7 cells were treated with the indicated doses of FIT-039 for 48 h. Cell viability was measured by MTT assays, and the IC_50_ values were the calculated using Reed-Muench method. The data were shown as the mean ± SD.

**
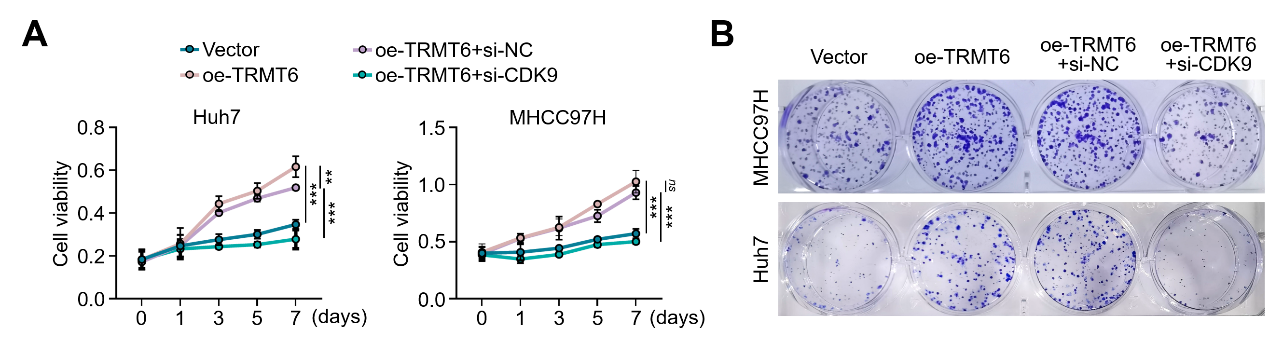
**

**Figure S16.** CDK9 knockdown abrogates malignant behaviors of HCC cells. TRMT6 was overexpressed and CDK9 was then knocked down in MHCC97H and Huh7 cells, and their effects on cell viability (A) colony formation (B) were further evaluated. The data were shown as the mean ± SD. **, *P* <0.01; ***, *P* <0.001; *ns*, no significance.

**
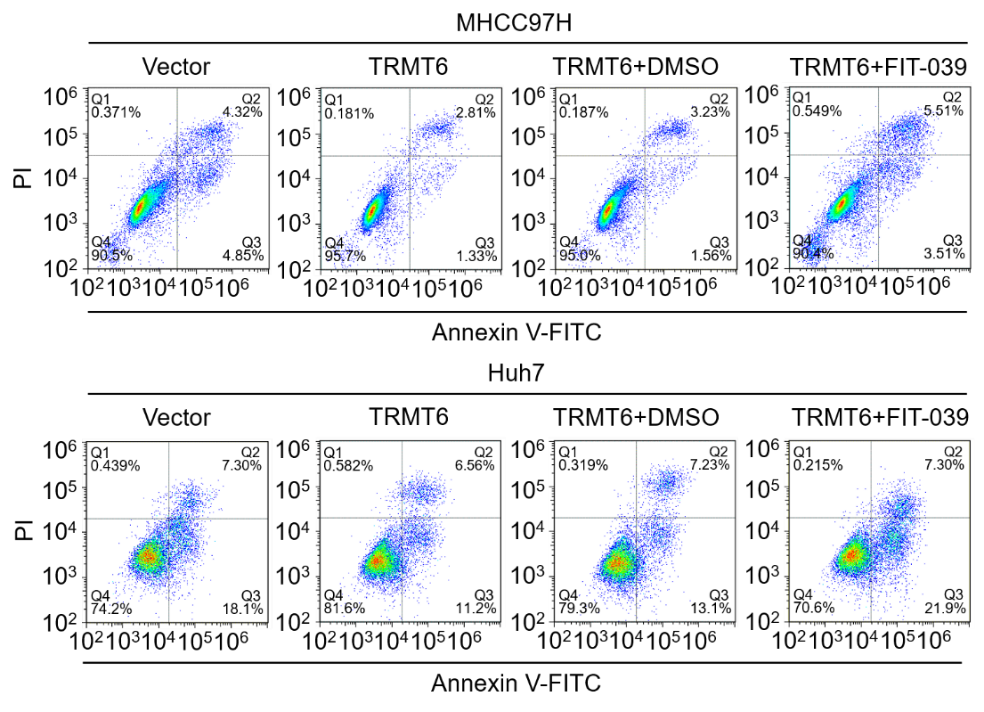
**

**Figure S17.** TRMT6 was overexpressed in MHCC97H (upper panel) and Huh7 cells (lower panel), and 10 μM FIT-039 was then added to the medium for 48 h. Their effect on cell apoptosis was evaluated by flow cytometry. Shown were the representative images of cell apoptosis (related to Fig. 6D).


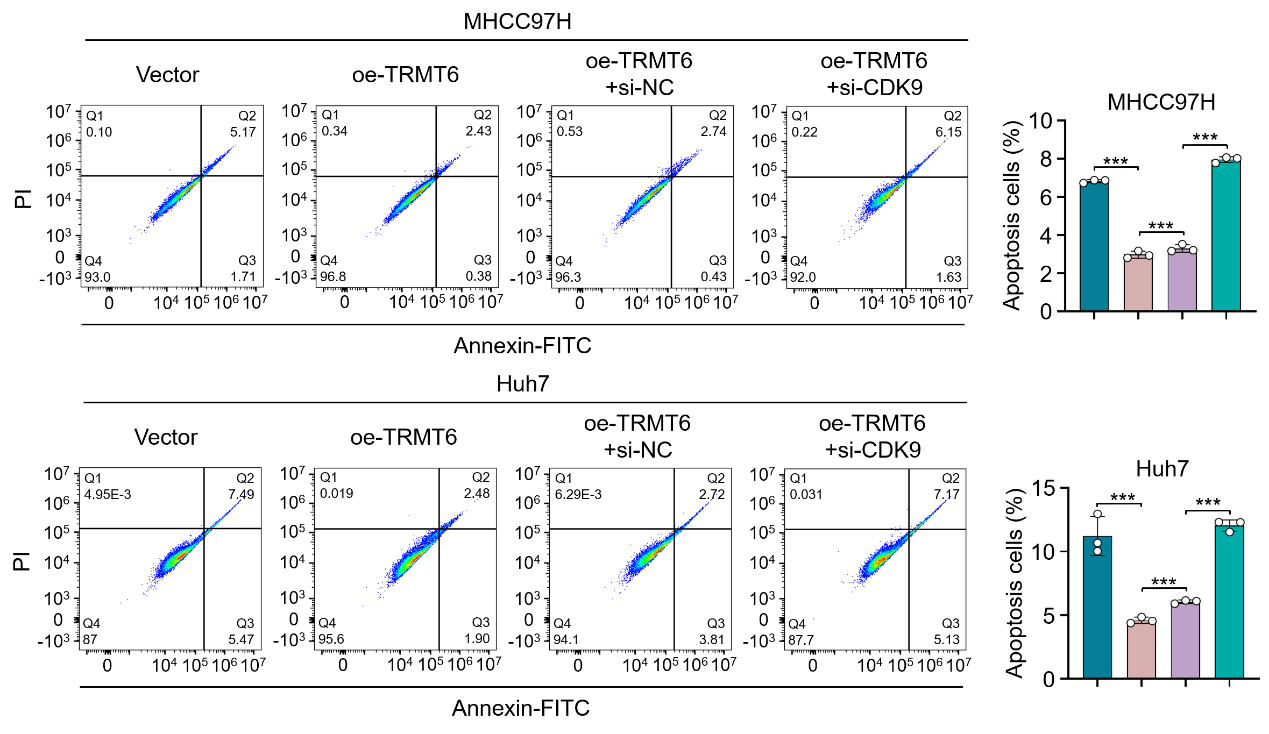


**Figure S18.** TRMT6 was overexpressed and CDK9 was then knocked down in MHCC97H and Huh7 cells, and its effects on cell apoptosis were further evaluated. The data were shown as the mean ± SD. ***, *P* <0.001.

**Figure S19.** CDK9 inhibition abrogates the tumorigenic ability of HCC cells in nude mice. A) The experiment procedure of TRMT6-overexpressing MHCC97H cell/control cell-derived xenograft tumor model. B) The positive area of TRMT6 and Ki-67 staining in the indicated tumors (related to Fig. 6G). C) Growth curves of body weight in the indicated groups. The data were shown as the mean ± SD. ***, *P* <0.001; *ns*, no significance.


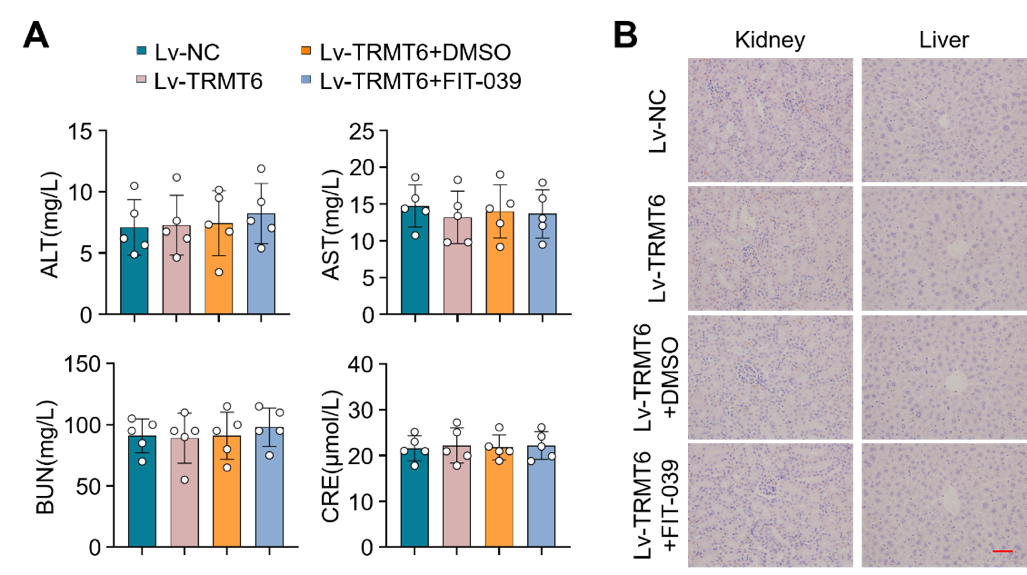


**Figure S20.** Safety evaluation of FIT-039 in nude mice. A) The levels of alanine transaminase (ALT), aspartate aminotransferase (AST), blood urea nitrogen (BUN) and serum creatinine (CRE) in the indicated groups. B) Representative H&E staining of kidney and liver sections from the indicated groups. Scale bar, 50 μm. Data were presented as mean ± SD.


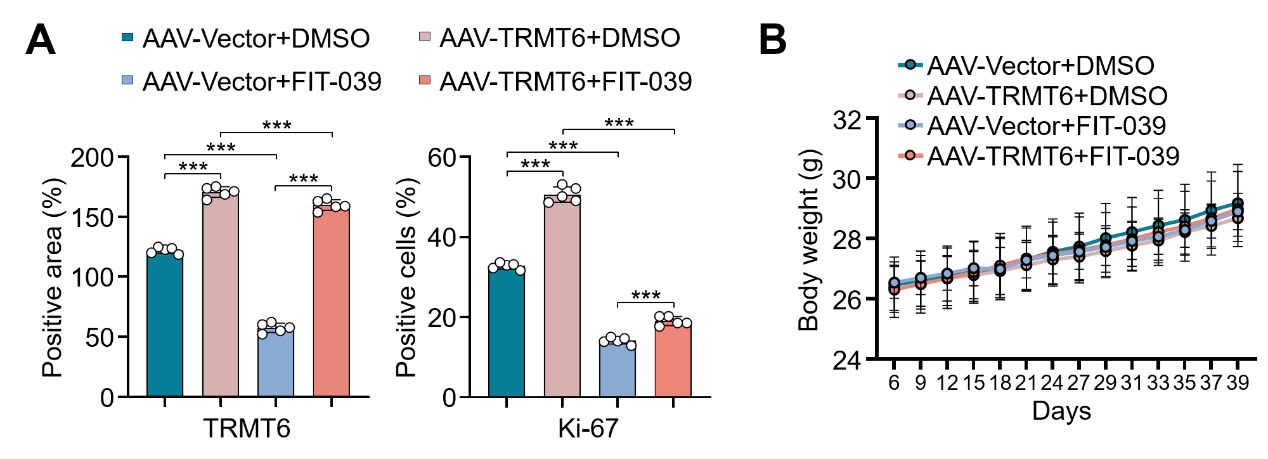


**Figure S21.** CDK9 inhibition abrogates the tumorigenesis ability of HCC tissues in PDX models using NCG mice. A) The positive area of TRMT6 and Ki-67 staining in the indicated tumors from PDX models (related to Fig. 6J). B) The growth curves of body weight in the indicated groups. The data were shown as the mean ± SD. ***, *P* <0.001.

**
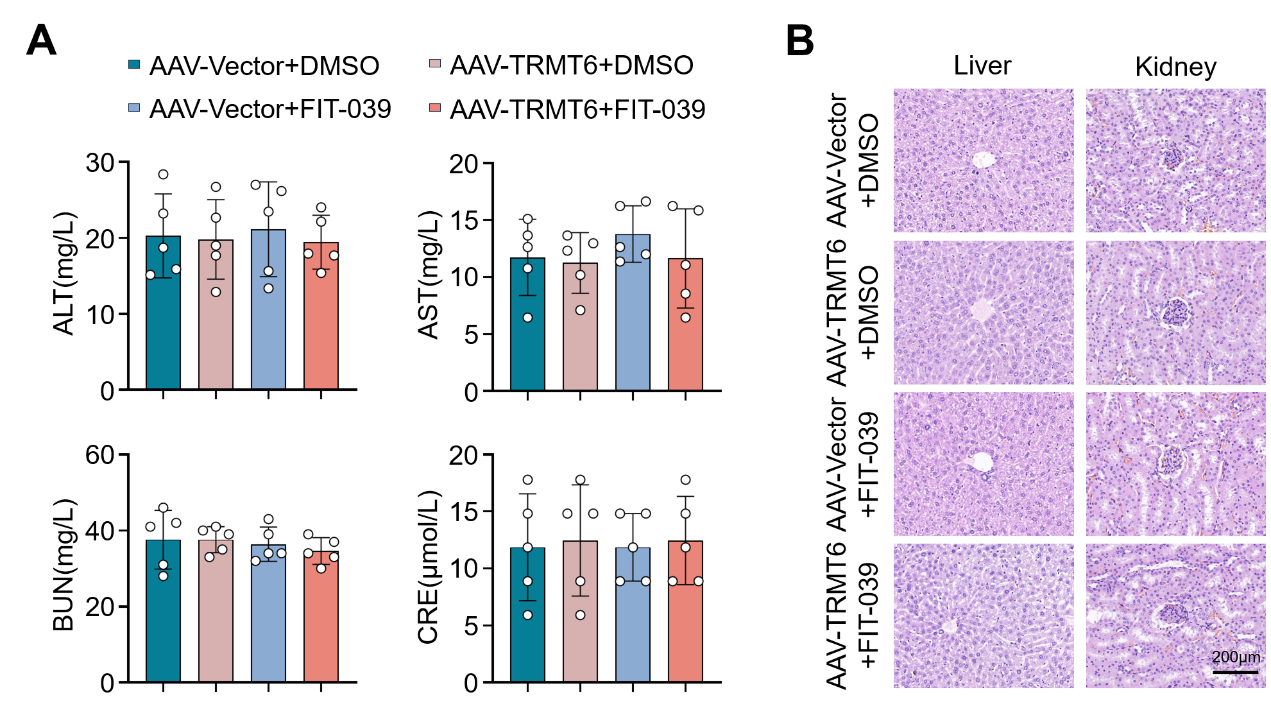
**

**Figure S22.** Safety evaluation of FIT-039 in PDX models. A) The levels of alanine transaminase (ALT), aspartate aminotransferase (AST), blood urea nitrogen (BUN) and serum creatinine (CRE) in the indicated groups. B) Representative H&E staining of kidney and liver sections from the indicated groups. Scale bar, 200 μm. Data were presented as mean ± SD.

**Figure S23.** The overexpression of TRMT6 by plasmids and the knockdown by siRNA were validated by qRT-PCR, *β-actin* serves as the internal control. The data were shown as the mean ± SD. ***, *P* <0.001; *ns*, no significance.

**Figure S24.** CDK9 knockdown abrogates HBV replication. A) TRMT6 was overexpressed and CDK9 was knocked down in HepAD38 cells. Then, the levels of pgRNA (left panel) were analyzed by qRT-PCR, the levels of cccDNA (middle panel) were measured by TaqMan-qPCR and the ratio of pgRNA/cccDNA was calculated (right panel). TRMT6 was overexpressed in HepG2.2.15 cells, and 10 μM FIT-039 was then added to the culture medium for 48 h. Next, the content of HBV DNA in cellular supernatant was detected using commercial kit (B), and mRNA and protein levels of HBx were analyzed by qRT-PCR (left panel) and western blotting (right panel) assays (C). *β-actin* was used as internal control for qRT-PCR and TaqMan-qPCR, and β-Actin was used as a loading control for western blotting analysis. The data were shown as the mean ± SD. **, *P* <0.01; ***, *P* <0.001; *ns*, no significance.


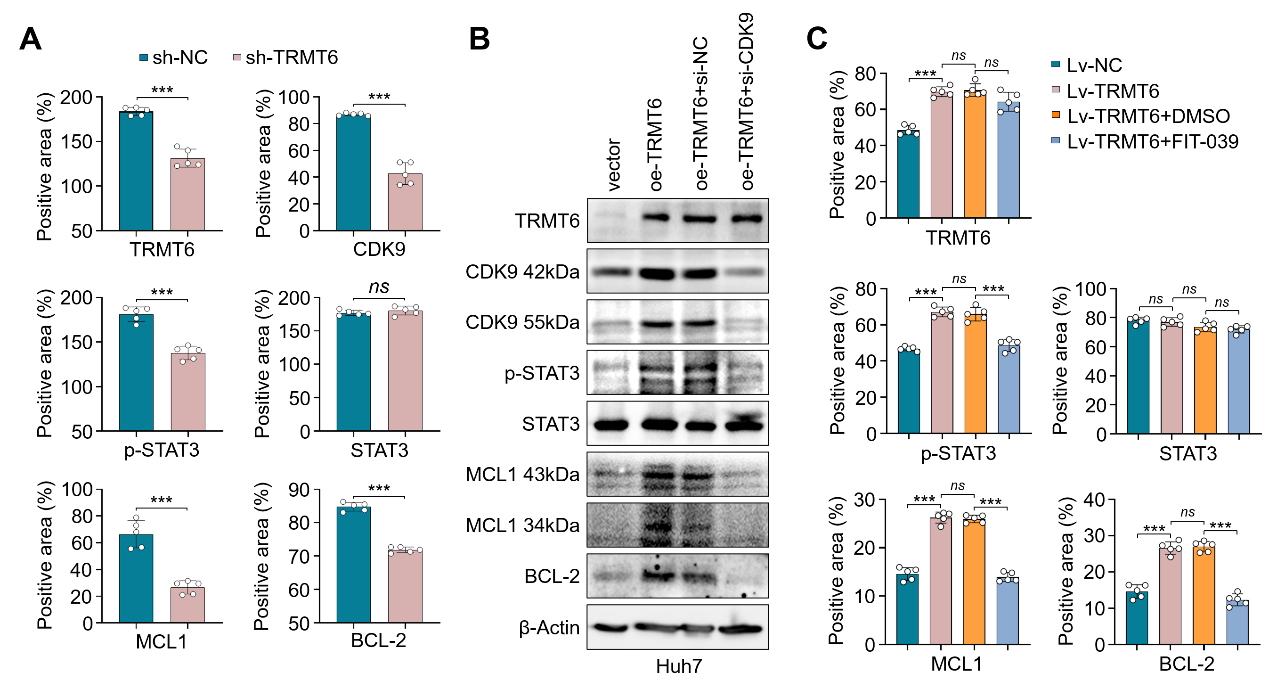


**Figure S25.** Oncogenic role of TRMT6 in HCC via CDK9-mediated up-regulation of oncogenic effectors. A) Positive area of TRMT6, CDK9, p-STAT3, STAT3, MCL1 and BCL-2 staining in the indicated tumors (related to Fig. 7B). B) TRMT6 was overexpressed and CDK9 was knocked down in Huh7 cells, and the downstream oncogenic factors of CDK9 were detected by western blotting assay. C) Positive area of TRMT6, p-STAT3, STAT3, MCL1 and BCL-2 staining in the indicated tumors (related to Fig. 7E). *β-actin* was used as internal control for qRT-PCR and TaqMan-qPCR, and β-Actin was used as a loading control for western blotting analysis. The data were shown as the mean ± SD. ***, *P* <0.001; *ns*, no significance.

**Figure S26.** The representative IHC staining of TRMT6, p-STAT3, MCL1 and BCL-2 in the indicated tumors (upper panel). Scale bar, 100 μm. Positive area of TRMT6, p-STAT3, STAT3, MCL1 and BCL-2 staining in the indicated tumors (lower panel). The data were shown as the mean ± SD. ***, *P* <0.001; *ns*, no significance.

**Figure S27.** The analysis of LC-MS/MS results. A) Co-IP assays coupled with LC-MS/MS analysis were performed in HepG2.2.15 cells using anti-CDK9 antibody, and the 15 virus-related terms were investigated by GO analysis. B) The lysates of HepG2.2.15 and HepG2 cells were subjected to Co-IP assays and LC-MS/MS analysis. The top-10 up-regulated and down-regulated molecules involved in viral process were shown by comparing differential unique peptides between HepG2.2.15 and HepG2 cells.


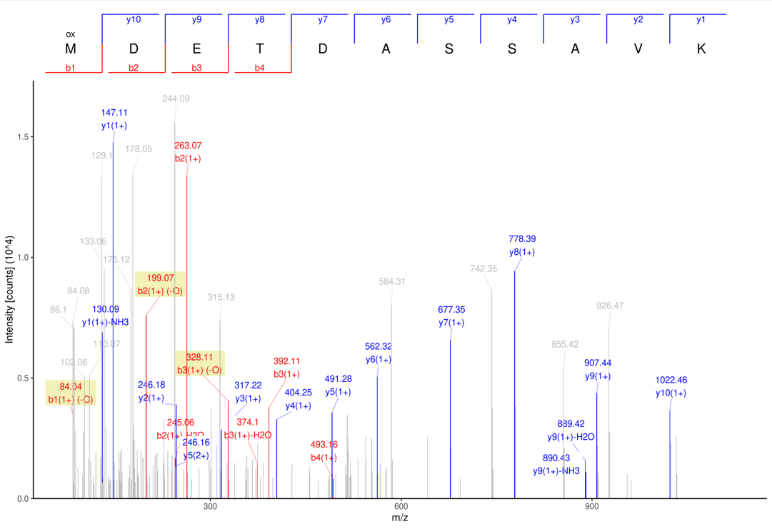


**Figure S****28.** The unique peptide of TARDBP. Co-IP assays coupled with LC-MS/MS analysis were performed in HepG2.2.15 cells using anti-CDK9 antibody. Shown was the representative image for unique peptide of TARDBP.

**Figure S29.** High expression of *TARDBP* predicts poor survival of HCC patients. A) The mRNA expression of *TARDBP* was analyzed in LIHC tissues (T) and control tissues (N) using TCGA database. B) The survival curves of HCC patients with high and low expression of *TARDBP*.

**
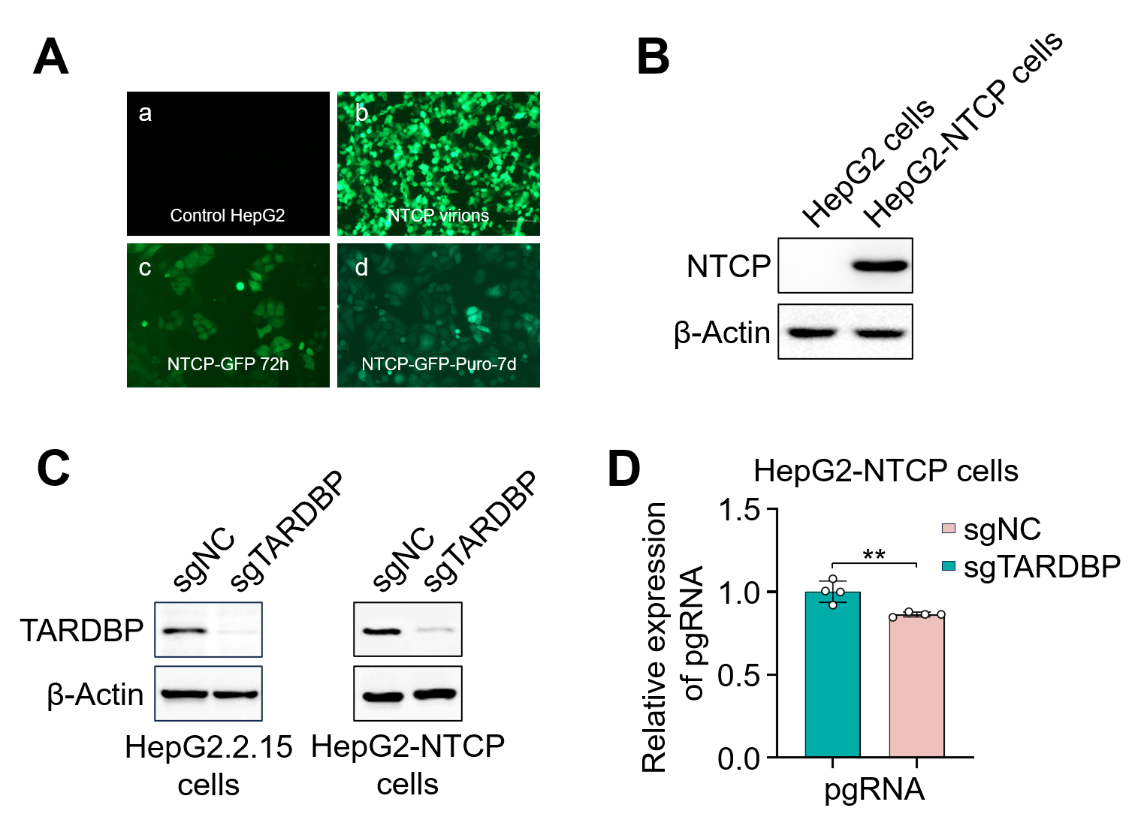
**

**Figure S30.** TARDBP knockout reduces pgRNA levels. A) The procedure of establishing HepG2-NTCP cells. HepG2 cells (a) were transfected with NTCP-GFP containing virions (b) for 72 h (c), and then supplemented with puromycin for 7 days (d). B) The expression of NTCP was measured by western blotting assay in HepG2 and HepG2-NTCP cells. C) TARDBP was knocked out using CRISPR-cas9 system in HepG2.2.15 and HepG2-NTCP cells. D) TARDBP was knocked out in HepG2-NTCP cells, and the mRNA expression of pgRNA was measured by qRT-PCR. *β-actin* was used as internal control for qRT-PCR and TaqMan-qPCR, and β-Actin was used as a loading control for western blotting analysis. The data were shown as the mean ± SD. **, *P* <0.01.

**
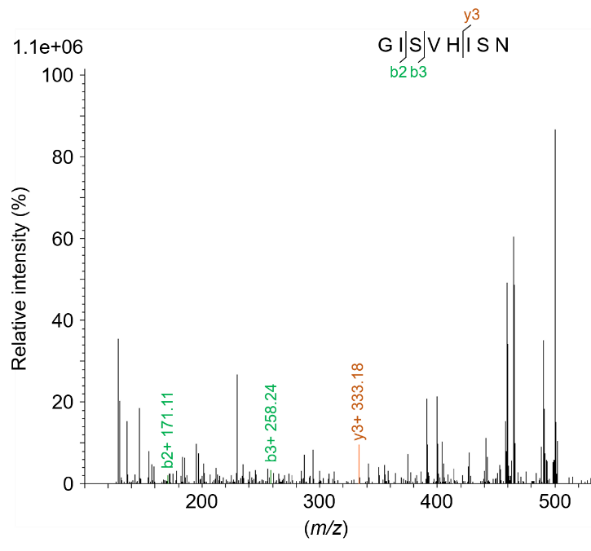
**

**Figure S31.** The phosphorylation of TARDBP at Ser254 is reduced upon CDK9 inhibition. HepG2.2.15 cells were treated with 10 μM FIT-039 or DMSO, and Co-IP assays and LC-MS/MS analysis were performed to detect the intensity of phosphorylation of TARDBP. Shown was the representative image for the phosphorylation of TARDBP at S254.

**
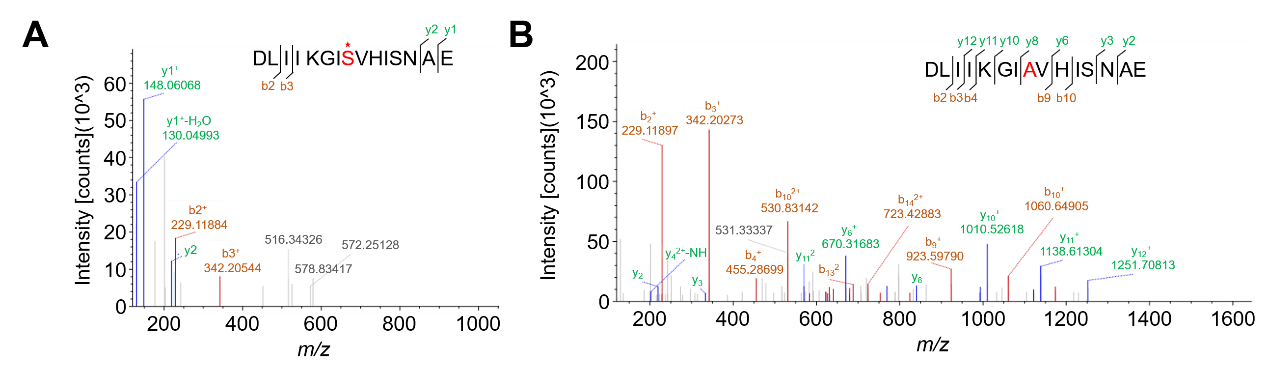
**

**Figure S32.** CDK9 phosphorylates TARDBP at S254. TARDBP S254-wild type peptides A) and TARDBP S254A-mutated peptides B) were mixed with CDK9/cyclinT1 recombined human protein and reacted *in vitro*. The phosphorylation sites were detected by LC-MS/MS. The representative images were showed in the panel.

**
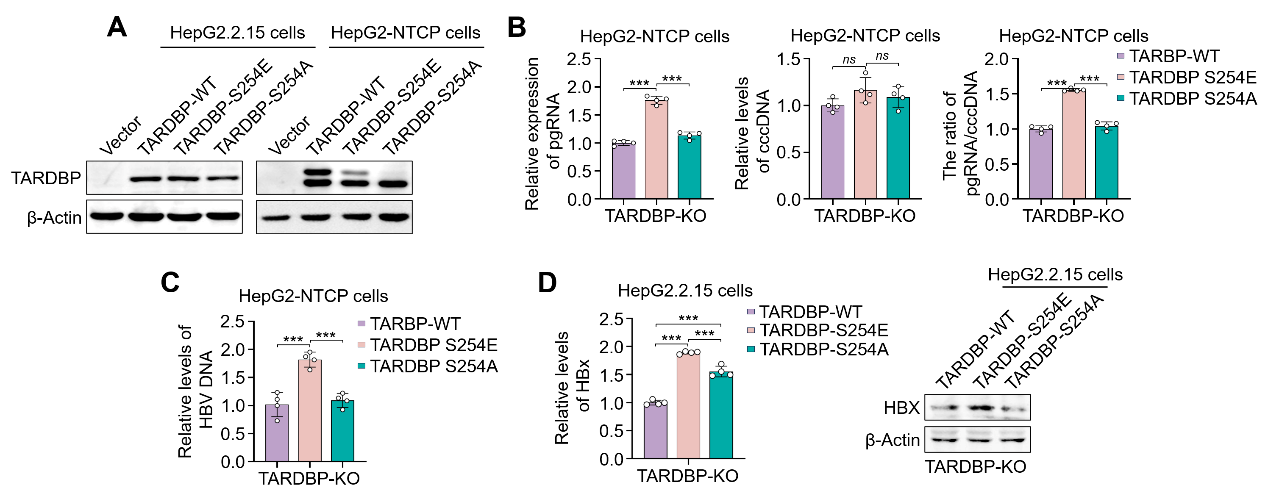
**

**Figure S33.** CDK9 enhances HBV replication via phosphorylation of TARDBP at Ser254. A) TARDBP were knocked out in HepG2.2.15 and HepG2-NTCP cells, and TARDBP-WT, -S254E and -S254A were then transfected into these cells. The overexpression of these plasmids was validated by western blotting analysis. B) TARDBP were knocked out in HepG2-NTCP cells, and TARDBP-WT, -S254E and -S254A were then transfected into these cells. Then, the levels of pgRNA (left panel) were evaluated by qRT-PCR, the levels of cccDNA (middle panel) were measured by TaqMan-qPCR and the ratio of pgRNA/cccDNA was calculated (right panel). C) TARDBP-knockout HepG2-NTCP cells were transfected with TARDBP-WT, -S254E and -S254A plasmids. Their effect on the content of HBV DNA in cellular supernatant was then determined using commercial kits. D) HepG2.2.15 cells were knocked out, and TARDBP-WT, -S254E and -S254A were then transfected into these cells. Their effects on the relative mRNA and protein expression of HBx were evaluated by qRT-PCR (left panel) and western blotting assays (right panel). *β-actin* was used as internal control of qRT-PCR and TaqMan-qPCR, β-Actin was used as a loading control for western blotting analysis. The data were shown as the mean ± SD. ***, *P* <0.001; *ns*, no significance.


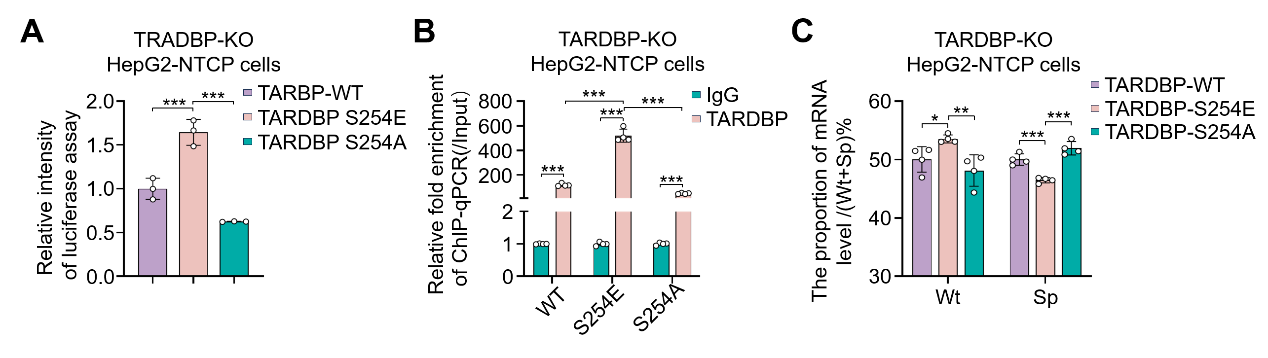


**Figure S34.** Phosphorylation of TARDBP at S254 promotes HBV replication. A) TARDBP was knocked out in HepG2-NTCP cells, and TARDBP-WT, -S254E and -S254A plasmids and luciferase reporter plasmid containing the HBV core promoter were then transfected into HepG2-NTCP cells for 48 h. Dual-luciferase reporter assays were then performed to assess the luciferase intensity with the intensity of Renilla luciferase as normalization control. B) TARDBP was knocked out in HepG2-NTCP cells, and TARDBP-WT, -S254E and -S254A plasmids were transfected into the above cells for 48 h. Their effect on the interaction of TARDBP with the HBV core promoter was determined by ChIP-qPCR. Input was used as normalization control. C) The plasmids containing TARDBP-WT, -S254E and -S254A were transfected into TARDBP-knockout HepG2-NTCP cells for 48 h. The relative levels of wild type (Wt) pgRNA and splicing variant (Sp) were evaluated by qRT-PCR. The proportions of mRNA level of Wt and Sp were then calculated. *β-actin* was used as internal control of qRT-PCR. The data were shown as the mean ± SD. *, *P* <0.05; **, *P* <0.01; ***, *P* <0.001; *ns*, no significance.

**Supplementary Tables**

**Table S1.** The terms related to the viral processes and life cycles (see excel files)

**Table S2.** The fold changes of molecules related to viral processes and life cycles

| **Gene names** | **Unique peptides**  **HepG2.2.15** | **Unique peptides**  **HepG2** | **Fold change（%）** |
| --- | --- | --- | --- |
| EIF3A | 3 | 1 | 3 |
| TARDBP | 2 | 1 | 2 |
| EIF3F | 2 | 1 | 2 |
| CANX | 7 | 4 | 1.75 |
| DDX5 | 7 | 4 | 1.75 |
| CCNT1 | 8 | 6 | 1.33 |
| PABPC1 | 4 | 3 | 1.33 |
| CDK9 | 9 | 7 | 1.29 |
| VCP | 5 | 4 | 1.25 |
| EEF1A1 | 6 | 5 | 1.2 |
| CHMP1A | 1 | 1 | 1 |
| DDX3X | 4 | 4 | 1 |
| EIF3L | 3 | 3 | 1 |
| HSP90AB1 | 5 | 5 | 1 |
| ILF3 | 4 | 4 | 1 |
| ITGB1 | 1 | 1 | 1 |
| LTF | 1 | 1 | 1 |
| PCBP1 | 4 | 4 | 1 |
| PDCD6IP | 2 | 2 | 1 |
| PPIA | 5 | 5 | 1 |
| RAB1A | 1 | 1 | 1 |
| RAN | 4 | 4 | 1 |
| SLC1A5 | 2 | 2 | 1 |
| SSB | 1 | 1 | 1 |
| STOM | 1 | 1 | 1 |
| HSPA1A | 7 | 8 | 0.88 |
| PPIB | 7 | 8 | 0.88 |
| PCBP2 | 2 | 3 | 0.67 |
| TRIM28 | 2 | 3 | 0.67 |
| P4HB | 8 | 13 | 0.62 |
| RPSA | 4 | 7 | 0.57 |
| LGALS1 | 1 | 2 | 0.5 |
| PTBP1 | 2 | 4 | 0.5 |
| TFRC | 3 | 10 | 0.3 |
| CFL1 | 1 | 4 | 0.25 |
| DEK | 1 | 4 | 0.25 |
| SLC3A2 | 2 | 8 | 0.25 |
| GSN | 1 | 5 | 0.2 |

**Table S3.** The effect of FIT-039 on phosphorylation intensity of TARDBP at Ser254 (see excel files)

**Table S4.** *In vitro* kinase activity assessment of CDK9 using TARDBP peptide (see excel files)

**Table S5.** TARDBP-S254 phosphorylation information from LC-MS (see excel files)

**Table S6.** TARDBP-S254 phosphorylation information from LC-MS (see text files)

**Table S7.** The sample information used for snRNA-seq analysis

| **Samples** | **Tissues** | **Diseases** | **HBV** |
| --- | --- | --- | --- |
| #1 | Liver | Normal | **-** |
| #2 | Liver | Normal | **-** |
| #3 | Liver | Tumor | **+** |
| #4 | Liver | Tumor | **+** |
| #5 | Liver | Tumor | **+** |
| #6 | Liver | Tumor | **+** |

**Abbreviations:** snRNA-seq: Single-nucleus RNA sequencing.

**Table S8.** The clinical data of the patients used for detecting the m^1^A levels

| **Patients** | **Sex** | **Age** | **HBV** | **HCV** | **AFP (μg/L)** |
| --- | --- | --- | --- | --- | --- |
| Patient#4 | Male | 55 | - | - | 1.3 |
| Patient#5 | Male | 59 | **+**（1,3,5） | - | 2.45 |
| Patient#6 | Female | 52 | - | - | >830 |
| Patient#7 | Male | 45 | **+**（1,4,5） | - |  |
| Patient#8 | Male | 25 | **+**（1,4,5） | - | 24208 |

**Table S9.** The clinical data of the frozen patient tissues used in this study

| **Controls** | **Sex** | **Age** | **HBV** | **HCV** | **AFP (μg/L)** |
| --- | --- | --- | --- | --- | --- |
| Control #1 | Male |  | - | - |  |
| Control #2 | Female |  | **+**（2,4,5+） |  |  |
| Control #3 | Male | 47 | **+** | **+** |  |
| Control #4 | Female | 59 | **+**（1） | - |  |
| Control #5 |  |  | **+**（2,5+） | - |  |
| Control #6 | Male | 50 | - | - |  |
| Control #7 | Male | 47 | **+**（2,4,5+） | - |  |
| Control #8 | Male |  | **+**（1,3,5） |  |  |
| Control #9 | Male | 39 | **+**（1,3,4,5） |  |  |
| Control #10 | Female | 35 | - | - |  |
| Control #11 | Male | 52 | **+**（1,4,5） | **+** |  |
| Control #12 | Male | 16 | - | - | 2.18 |
| Control #13 | Female | 48 | **+**（1,4,5） | - | 2.24 |
| Control #14 | Male | 66 | **+**（1,4,5） | - | 14.49 |
| Control #15 | Female | 59 | - | **+** | 0.66 |
| Control #16 | Male | 36 | **+**（1,4,5） | - | - |
| Control #17 | Female | 39 | **+** | **+** | 3.48 |
| Control #18 | Male | 38 | **+**（1,4,5） | - | 1.65 |
| Control #19 | Male | 44 | - | - | - |
| Control #20 | Male | 57 | - | - |  |
| Control #21 | Female | 56 | **+**（1,3,5） | - |  |
| Control #22 | Female |  | **+** | - | 47 |
| Control #23 | Male | 40 | **+**（1,4,5） | - |  |
| Patient#1 | Male | 66 | - | - |  |
| Patient#2 | Male | 41 |  |  | 7.3 |
| Patient#3 | Female | 64 | - | - | 20.72 |
| Patient#4 | Male | 55 | - | - | 1.3 |
| Patient#5 | Male | 59 | **+**（1,3,5） | - | 2.45 |
| Patient#6 | Female | 52 | - | - | >830 |
| Patient#7 | Male | 45 | **+**（1,4,5） | - |  |
| Patient#8 | Male | 25 | **+**（1,4,5） | - | 24208 |
| Patient#9 | Male | 54 | **+**（1） | **+** | 38 |
| Patient#10 | Male | 60 | - | **+** | 2.12 |
| Patient#11 | Male | 67 | **+**（1） | - | 323.4 |
| Patient#12 | Female | 59 | **+**（1,4,5） | - | 3267 |
| Patient#13 | Male | 58 | **+**（1） | - | 1.28 |
| Patient#14 | Male | 45 | **+**（1,3,5） | - | 3.3 |
| Patient#15 | Male | 63 | **+**（1,3,5） | - | 20.49 |
| Patient#16 | Male | 38 | **+**（1,4,5） | - | 72.69 |
| Patient#17 | Male | 53 | **+**（1,3,5） | **+** | 12.46 |
| Patient#18 | Male | 72 | **+**（1,4,5） | - | 3.11 |
| Patient#19 |  |  | **+** (1,4,5) |  | >830 |
| Patient#20 | Female | 49 | - | - | - |
| Patient#21 |  |  | **+**（1,3,5） |  |  |
| Patient#22 |  |  | **+**（1,4,5） |  |  |
| Patient#23 |  |  | **+**（1,3,5） |  |  |
| Patient#24 |  |  | - | - |  |
| Patient#25 |  |  | **+**（1,3,5） |  |  |
| Patient#26 |  |  | **+**（1,4,5） |  |  |
| Patient#27 |  |  | **+**（1,4,5） |  |  |
| Patient#28 |  |  | **+**（1） |  |  |
| Patient#29 |  |  | **+**（1,3,5） |  |  |
| Patient#30 |  |  | - | - |  |

**Table S10.** The clinical data of paraffin sections used in this study to evaluate the protein levels of TRMT6 and CDK9

| **Patients** | **Pathology number** | **Sex** | **Age** | **HBV** | **HBV DNA** |
| --- | --- | --- | --- | --- | --- |
| Patient #a | B201941116 | Male | 41 | **+** | **+** |
| Patient #b | B201936687 | Male | 44 | **+** | **+** |
| Patient #c | B201942519 | Male | 48 | **+** | **+** |
| Patient #d | B201937925 | Male | 46 | **+** |  |
| Patient #e | B201936414 | Female | 55 | **+** | **+** |
| Patient #f | B201933027 | Male | 43 | **+** | **+** |
| Patient #g | B201933036 | Male | 61 | **+** | **-** |
| Patient #h | B201934096 | Male | 52 | **+** | **+** |
| Patient #i | B201934322 | Male | 55 | **-** | **-** |
| Patient #j | B201934376 | Male | 60 | **-** | **-** |
| Patient #k | B201934687 | Male | 38 | **+** | **+** |
| Patient #l | B201935023 | Male | 66 | **+** | **-** |
| Patient #m | B201935081 | Female | 34 | **+** | **+** |
| Patient #n | B201935438 | Male | 56 | **+** | **-** |
| Patient #o | B201936216 | Male | 46 | **+** | **-** |
| Patient #p | B201936889 | Male | 63 | **+** | **-** |
| Patient #q | B201938369 | Male | 59 | **+** | **-** |
| Patient #r | B201939477 | Male | 60 | **+** | **-** |
| Patient #s | B201939741 | Male | 40 | **+** | **-** |
| Patient #t | B201941113 | Female | 69 | **+** | **-** |
| Patient #u |  |  |  |  |  |
| Patient #v |  |  |  |  |  |
| Patient #w |  |  |  |  |  |
| Patient #x |  |  |  |  |  |

**Table S11.** The clinical data of HCC tissues used for PDX model establishment

| **Patients** | **Sex** | **Age** | **HBV** | **HCV** | **AFP (μg/L)** |
| --- | --- | --- | --- | --- | --- |
| Patient#1 | Female | 68 | + | - | 1.46 |
| Patient#2 | Male | 45 | + | - | 451 |

**Table S12.** The primers used in this study

| **Genes** | **Primer sequences** |
| --- | --- |
| Human *TRMT6* | F (5’-3’), ACTGTTGTGAAGCCATCCA; |
|  | R (3’-5’), TGTTGCCAGCACGGATAT. |
| Human *TRMT61A* | F (5’-3’), GGCTCTGTGGTCTGTGAGTCT; |
|  | R (3’-5’), GATGGGATGTCCAGGAAGACG. |
| Human *MRPL4* | F (5’-3’), ACCTGCACATCATGGACTCCCT; |
|  | R (3’-5’), ATGCTCTGTGGCATCTCCTCGT. |
| Human *CDK9* | F (5’-3’), ATGGCAGAGATGTGGACC; |
|  | R (3’-5’), TCCTTCACCTTCCGCTTC. |
| Human *TARDBP* | F (5’-3’), CCTTGCGTTCATAGCGTTGATAC; |
|  | R (3’-5’), TGCCATAGGAATACTGTCTACATGC. |
| Human *18S* rRNA | F (5’-3’), CAGCCACCCGAGATTGAGCA; |
|  | R (3’-5’), TAGTAGCGACGGGCGGTGTG. |
| Human *β-actin* | F (5’-3’), GCACAGAGCCTCGCCTT; |
|  | R (3’-5’), GTTGTCGACGACGAGCG. |
| HBx | F (5’-3’), CCCGTCTGTGCCTTCTCATC; |
|  | R (3’-5’), CCCAACTCCTCCCAGTCTTT. |
| pgRNA | F (5’-3’), GCCTTAGAGTCTCCTGAGCA; |
|  | R (5’-3’), GAGGGAGTTCTTCTTCTAGG. |
| pgRNA Wt | F (5’-3’), TCTAGACTCGTGGTGGACTTCTCTC |
|  | R (5’-3’), CATAGCAGCAGGATGAAGAGGAA |
| pgRNA Sp | F (5’-3’), GCGTCGCCGAAGATCTCA; |
|  | R (5’-3’), GGATGGGAATACAAGTGCAGTTT. |

**Table S13.** The primary antibodies used in this study

| **Antibodies** | **Catalog#** | **Source** | **Dilution** |
| --- | --- | --- | --- |
| CGI-09 Recombinant Protein Antigen | Novus | NBP2-13486 | 1:3000 |
| Anti-CDK9 antibody | Abcam | ab76320 | 1:5000 |
| Anti-MRPL4 antibody | Proteintech | 27484-1-AP | 1:1000 |
| TDP 43 Rabbit mAb | Zenbio | R25881 | 1:1000 |
| BD Pharmingen™ Purified Mouse Anti-Ki-67 | BD Pharmingen | 550609 | 1:1000 |
| Normal Rabbit IgG | Cell Signaling Technology | 2729 |  |
| Anti-1-methyladenosine (m1A) antibody | Abcam | ab208196 | 1:1000 |
| MCL1 Rabbit mAb | Zenbio | R22875 | 1:1000 |
| Human BCL2 Polyclonal antibody | Proteintech | 12789-1-AP | 1:1000 |
| Phospho-Stat3 (Tyr705) (D3A7) XP® Rabbit mAb | Cell Signaling Technology | 9145S | 1:1000 |
| Stat3 (124H6) Mouse mAb | Cell Signaling Technology | 9139S | 1:1000 |
| beta Actin Ab | Abways | AB0035 | 1:10000 |
| Anti-Hepatitis B Virus X antigen | Abcam | ab39716 | 1:1000 |
| HRP-conjugated anti-rabbit IgG | ZSGB-Bio | ZB-2301 | 1:2500 |

**Table S14.** The primers and TaqMan probes used in HBV cccDNA detection

| **Items** | **Sequences** |
| --- | --- |
| HBV cccDNA | F (5’-3’), GGGGCGCACCTCTCTTTA |
|  | R (3’-5’), AGGCACAGCTTGGAGGC |
| β-actin qPCR | F (5’-3’), TCACCCACACTGTGCCCATCTACGA |
|  | R (3’-5’), TCGGTGAGGATCTTCATGAGGTA |
| TaqMan probe B1 | TCACCTCTGCCTAATCATCTC |
| TaqMan probe A1 | ATGCCCTCCCCCATGCCATCC |

**Table S15.** The sequences of siRNAs used in this study

| **siRNAs** | **Sequences（5’-3’）** |
| --- | --- |
| si-NC | UUCUCCGAACGUGUCACGUTT |
| si-TRMT6#1 | GGACAGTCTTCTACATGGA |
| si-TRMT6#2 | GGTCTACTGTCAGTACAAA |
| si-CDK9 | GGAGAAUUUUACUGUGUUUdTdT |

**Table S16.** The sequences of shRNAs and sgRNA used in this study

| **sh/sg-RNAs** | **Sequences（5’-3’）** |
| --- | --- |
| sh-TRMT6#1 | GGGCACTGATAATCGAAAT |
| sh-TRMT6#2 | GGTTCTACCTGGATAACGT |
| sg-TARDBP | TGAGCCCATTGAAATACCAT |

**Table S17.** Off-target and efficiency of sg-TARDBP (see csv file)

**Table S18.** The primers used for MeRIP-qPCR

| **Primers** | **Primer sequences** |
| --- | --- |
| CDK9-U | F（5’-3’），GCCTATGTGCGTGACCCAT; |
|  | R（5’-3’），ACCAGAAGAAGTCGTGGTTGA. |
| CDM9-M | F（5’-3’），GCCCTCAACCACGACTTCTT; |
|  | R（5’-3’），GGATTGCGACTCTGGTTGGT. |

**Table S19.** The primers used for ChIP-qPCR

| **Primer** | **Primer sequences** |
| --- | --- |
| HBV CP | F（5’-3’），GAGACCACCGTGAACGCCCACCA |
|  | R（5’-3’），AAGAGATGATTAGGCAGAG |

**Abbreviations:** ChIP: Chromatin Immunoprecipitation; CP: Core promoter.

**Table S20.** The peptides used in In vitro kinase activity assay

| Peptides | Sequence | Purity |
| --- | --- | --- |
| TARDBP S254 | DLIIKGISVHISNAE | 95.99% |
| TARDBP S254A | DLIIKGIAVHISNAE | 95.79% |
