# Supplementary material for: TRMT6‐Mediated m1A Modification of CDK9 mRNA is a Dual‐Pronged Pathogenic Driver for HBV‐Related Hepatocellular Carcinoma
Source: Adv Sci (Weinh). 2026 Apr 20;13(39):e14172. doi: 10.1002/advs.202514172 (PMC13334973; doi:10.1002/advs.202514172)
Supplement: Supplementary file 2 — Supporting File 2: advs75266‐sup‐0002‐Data.zip. [file ADVS-13-e14172-s001.zip › GSE Analysis code.pdf]

In [ ]:
```
setwd('./matrix+loom/release/mtx')
getwd()
```

In [ ]:
```
library(tidyverse)
library(Seurat)
library(DoubletFinder)
library(ggpubr)
library(ggplot2)
library(egg)
library(clustree)
library(harmony)
```

In [ ]:
```
theme_dimplot <- theme(legend.title=element_blank(),
      legend.text=element_text(colour="black", size=20),
      axis.text=element_text(color="black", size=20),
      axis.title=element_blank(),
      axis.line=element_line(colour="black"),
      panel.background=element_rect(fill="white"),
      panel.border=element_rect(fill=NA,color="black", linewidth=2, linetype=
```

In [ ]:
```
process_sample <- function(sample_name, rna_dir, virus_csv = NULL) {

  cat("Processing:", sample_name, "\n")
  cat(" - Loading RNA data from:", rna_dir, "\n")
  rna_data <- Read10X(data.dir = rna_dir, strip.suffix = TRUE)
  seurat_obj <- CreateSeuratObject(
    counts = rna_data,
    project = sample_name,
    min.cells = 3,
    min.features = 200
  )

  if (!is.null(virus_csv) && file.exists(virus_csv)) {
    cat(" - Loading virus data from:", virus_csv, "\n")
    virus_data <- read.csv(virus_csv, row.names = "barcode")
    cat(" - Virus data dimensions:", dim(virus_data), "\n")

    seurat_obj$virus_LC <- 0
    seurat_obj$virus_AB <- 0
    seurat_obj$virus_total <- 0
    matched_cells <- intersect(rownames(virus_data), colnames(seurat_obj))
    cat(" - Found", length(matched_cells), "cells with virus data\n")
    if (length(matched_cells) > 0) {
      seurat_obj@meta.data[matched_cells, "virus_LC"] <- virus_data[matched_c
      seurat_obj@meta.data[matched_cells, "virus_AB"] <- virus_data[matched_c
      seurat_obj@meta.data[matched_cells, "virus_total"] <- virus_data[matche
    }
    seurat_obj$HBV_POS <- seurat_obj$virus_total > 0
    cat(" -", sum(seurat_obj$HBV_POS), "virus-positive cells identified\n")

  } else {
    cat(" - No virus data provided or file not found\n")
    seurat_obj$virus_LC <- NA
    seurat_obj$virus_AB <- NA
    seurat_obj$virus_total <- NA
    seurat_obj$HBV_POS <- NA
  }
```

```
  seurat_obj$has_virus_data <- !is.null(virus_csv) && file.exists(virus_csv)

  return(seurat_obj)
}

sample_info <- list(

  list(name = "Sample_01",
       rna_dir = "./1NT_P21"),

  list(name = "Sample_02",
       rna_dir = "./2NT_P24"),

  list(name = "Sample_03",
       rna_dir = "./3NT_P25"),

  list(name = "Sample_04",
       rna_dir = "./4NT_P29"),

  list(name = "Sample_05",
       rna_dir = "./5NT_P36"),

  list(name = "Sample_06",
       rna_dir = "./zl/A_09_ZL_filtered_feature_bc_matrix",
       virus_csv = "./fj/A_09_FJ_filtered_UMI_new.csv"),

  list(name = "Sample_07",
       rna_dir = "./zl/A_10_ZL_filtered_feature_bc_matrix",
       virus_csv = "./fj/A_10_FJ_filtered_UMI.csv"),

  list(name = "Sample_08",
       rna_dir = "./zl/A_08_1_drop_ZL_filtered_feature_bc_matrix",
       virus_csv = "./fj/A_08_1_drop_FJ_filtered_UMI.csv"),

  list(name = "Sample_09",
       rna_dir = "./zl/SF_B1_1_ZL_filtered_feature_bc_matrix",
       virus_csv = "./fj/SF_B1_1_FJ_filtered_UMI.csv"),

  list(name = "Sample_10",
       rna_dir = "./zl/SF_B2_1_ZL_filtered_feature_bc_matrix",
       virus_csv = "./fj/SF_B2_1_FJ_filtered_UMI.csv"),

  list(name = "Sample_11",
       rna_dir = "./zl/SF_B4_3_ZL_filtered_feature_bc_matrix",
       virus_csv = "./fj/SF_B4_3_FJ_filtered_UMI.csv")
)

for (i in seq_along(sample_info)) {
  info <- sample_info[[i]]

  if ("virus_csv" %in% names(info)) {
    obj <- process_sample(
      sample_name = info$name,
      rna_dir = info$rna_dir,
      virus_csv = info$virus_csv
    )
  } else {
    obj <- process_sample(
      sample_name = info$name,
      rna_dir = info$rna_dir
    )
  }

  assign(info$name, obj, envir = .GlobalEnv)
```

```
  cat("\nCreated object:", info$name, "\n")
  cat(" - Cells:", ncol(get(info$name)), "\n")
  if ("virus_csv" %in% names(info)) {
    cat(" - Virus positive cells:", sum(get(info$name)$HBV_POS, na.rm = TRUE)
  } else {
    cat(" - No virus data available\n")
  }
  cat(" - Has virus data:", get(info$name)$has_virus_data[1], "\n\n")
}
```

In [ ]:

```
doublet_Function <- function(seurat_obj) {

  data <- NormalizeData(seurat_obj) %>%
    FindVariableFeatures(selection.method = "vst", nfeatures = 2000) %>%
    ScaleData(verbose = FALSE) %>%
    RunPCA(npcs = 30, verbose = FALSE) %>%
    RunUMAP(dims = 1:30) %>% FindNeighbors(dims = 1:30) %>%
    FindClusters(resolution = 0.5)

  sweep.res.list <- paramSweep_v3(data, PCs = 1:30, sct = FALSE)
  sweep.stats <- summarizeSweep(sweep.res.list, GT = FALSE)
  bcmvn <- find.pK(sweep.stats)
  pK_bcmvn <- bcmvn$pK[which.max(bcmvn$BCmetric)] %>% as.character() %>% as.n
  DoubletRate = ncol(data)*8*1e-6
  annotations <- data@meta.data$seurat_clusters
  homotypic.prop <- modelHomotypic(annotations)
  nExp_poi <- round(DoubletRate*ncol(data))
  nExp_poi.adj <- round(nExp_poi*(1-homotypic.prop))

 data <- doubletFinder_v3(data, PCs = 1:30, pN = 0.25, pK = pK_bcmvn, nExp =
  seurat_obj$doubFind_res = data@meta.data %>% select(contains('DF.classifica
  seurat_obj$doubFind_score = data@meta.data %>% select(contains('pANN'))
  return(seurat_obj)
}
```

In [ ]:

```
Sample_01 <- doublet_Function(Sample_01)
print(head(Sample_01@meta.data, 5))
Sample_02 <- doublet_Function(Sample_02)
print(head(Sample_02@meta.data, 5))
Sample_03 <- doublet_Function(Sample_03)
print(head(Sample_03@meta.data, 5))
Sample_04 <- doublet_Function(Sample_04)
print(head(Sample_04@meta.data, 5))
Sample_05 <- doublet_Function(Sample_05)
print(head(Sample_05@meta.data, 5))
Sample_06 <- doublet_Function(Sample_06)
print(head(Sample_06@meta.data, 5))
Sample_07 <- doublet_Function(Sample_07)
print(head(Sample_07@meta.data, 5))
Sample_08 <- doublet_Function(Sample_08)
print(head(Sample_08@meta.data, 5))
Sample_09 <- doublet_Function(Sample_09)
print(head(Sample_09@meta.data, 5))
Sample_10 <- doublet_Function(Sample_10)
print(head(Sample_10@meta.data, 5))
Sample_11 <- doublet_Function(Sample_11)
print(head(Sample_11@meta.data, 5))
```

In [ ]:

```
sce_obj <- merge(Sample_01,y = c(Sample_02,Sample_03,Sample_04,Sample_05,
                                 Sample_06,Sample_07,Sample_08,Sample_09,
```

```
                                    Sample_10,Sample_11),project = "HBV")
```

In [ ]:

```
sce_obj$Accession <- sce_obj$orig.ident
replacement <- c( "Sample_01"="GSE242889","Sample_02"="GSE242889","Sample_03"
                 "Sample_04"="GSE242889","Sample_05"="GSE242889",
                 "Sample_06"="Own_data", "Sample_07"="Own_data","Sample_08"="
                 "Sample_09"="Own_data", "Sample_10"="Own_data", "Sample_11"=
sce_obj$Accession <- replacement[sce_obj$Accession]
level_Accession = c('GSE242889', 'Own_data')
sce_obj$Accession <- factor(sce_obj$Accession, levels = level_Accession, orde
unique(sce_obj$Accession)

level_orig.ident = c('Sample_01','Sample_02','Sample_03','Sample_04','Sample_
                     'Sample_06','Sample_07','Sample_08','Sample_09','Sample_
sce_obj$orig.ident <- factor(sce_obj$orig.ident, levels = level_orig.ident, o
unique(sce_obj$orig.ident)

sce_obj$disease <- sce_obj$orig.ident
replacement <- c( "Sample_01"="N", "Sample_02"="N", "Sample_03"="N",
                 "Sample_04"="N", "Sample_05"="N", "Sample_06"="N",
                 "Sample_07"="N", "Sample_08"="T", "Sample_09"="T",
                 "Sample_10"="T", "Sample_11"="T")
sce_obj$disease <- replacement[sce_obj$disease]
level_disease = c('N', 'T')
sce_obj$disease <- factor(sce_obj$disease, levels = level_disease, ordered =
unique(sce_obj$disease)
```

In [ ]:

```
sce <- subset(sce_obj, doubFind_res == 'Singlet')
dim(sce)
```

In [ ]:

```
sce[["percent.mt"]] <- PercentageFeatureSet(sce, pattern = "^MT")

p1=VlnPlot(sce, features = c("nFeature_RNA"),group.by='orig.ident', pt.size =
p2=VlnPlot(sce, features = c("nCount_RNA"), group.by='orig.ident', pt.size =
p3=VlnPlot(sce, features = c("percent.mt"), group.by='orig.ident', pt.size =
p1
p2
p3
p=ggarrange(p1, p2, p3, labels = c("A", "B", "C"),ncol = 1,nrow = 3)
ggsave("./QC_all.pdf",p,width = 8, height = 24)

FeatureScatter(sce, feature1 = "nCount_RNA", feature2 = "percent.mt", raster=
theme(axis.text = element_text(colour = 'black',size = 30))
FeatureScatter(sce, feature1 = "nCount_RNA", feature2 = "nFeature_RNA", raste
theme(axis.text = element_text(colour = 'black',size = 30))
```

In [ ]:

```
sce <- subset(sce, subset = nFeature_RNA > 200 & nFeature_RNA < 6000 & percen
dim(sce)
```

In [ ]:

```
p1=VlnPlot(sce, features = c("nFeature_RNA"),group.by='orig.ident', pt.size =
theme(axis.text = element_text(colour = 'black',size = 30))
p2=VlnPlot(sce, features = c("nCount_RNA"),group.by='orig.ident', pt.size = 0
theme(axis.text = element_text(colour = 'black',size = 30))
p3=VlnPlot(sce, features = c("percent.mt"),group.by='orig.ident', pt.size = 0
theme(axis.text = element_text(colour = 'black',size = 30))
p1
p2
```

```
p3

FeatureScatter(sce, feature1 = "nCount_RNA", feature2 = "percent.mt", raster=
theme(axis.text = element_text(colour = 'black',size = 30))
FeatureScatter(sce, feature1 = "nCount_RNA", feature2 = "nFeature_RNA", raste
theme(axis.text = element_text(colour = 'black',size = 30))
```

In [ ]:

```
# ############################################################################
# ## Normalization
# ############################################################################
sce <- NormalizeData(sce, normalization.method = "LogNormalize", scale.factor

# ############################################################################
# ## Feature selection
# ############################################################################
sce <- FindVariableFeatures(sce, selection.method = "vst", nfeatures = 2000)
# Identify the 10 most highly variable genes
top10 <- head(VariableFeatures(sce), 10)
# plot variable features with and without labels
plot1 <- VariableFeaturePlot(sce)
plot2 <- LabelPoints(plot = plot1, points = top10, repel = TRUE)
plot2

all.genes <- rownames(sce)
sce <- ScaleData(sce, features = all.genes)
```

In [ ]:

```
sce <- RunPCA(sce, features = VariableFeatures(object = sce))
DimPlot(sce, reduction = "pca", raster=FALSE)

DimHeatmap(sce, dims = 1:2, cells = 200, balanced = TRUE)
ElbowPlot(sce, ndims = 50)

sce <- RunUMAP(sce, dims = 1:50) # umap tsne
FeaturePlot(sce, features = c("IGLC2", "IGHG1", "DLK1"), reduction = "umap",
```

In [ ]:

```
p=ggarrange(p1, p2, p3, labels = c("A", "B", "C"),ncol = 1,nrow = 3)

ggsave("./QC.pdf",p,width = 8, height = 24)
```

In [ ]:

```
Harmony_Function <- function(data, group.by, lambda = 1, dims.use = 1:30) {
  Har <- RunHarmony(data, group.by = group.by, lambda = lambda,
                    theta = 2, plot_convergence = TRUE, dims = dims.use)

  DimPlot(object = Har, reduction = "harmony", pt.size = 0.1, group.by = grou
  VlnPlot(object = Har, features = c("harmony_1", "harmony_2"), group.by = gr

  Har <- Har %>%
    RunUMAP(reduction = "harmony", dims = dims.use) %>%
    FindNeighbors(reduction = "harmony", dims = dims.use) %>%
    FindClusters(resolution = 1.0) %>%
    identity()
    return(Har)
}
```

In [ ]:

```
sce <- Harmony_Function(sce, group.by = "orig.ident", lambda = 1, dims.use =
```

In [ ]:
```
DimPlot_function <- function(SeuratObj, group.by, figure_outpath) {
  plot <- DimPlot(SeuratObj, reduction = "umap", group.by = group.by, raster
  ggsave(paste0(figure_outpath,"/DimPlot_", group.by, ".png"),
       egg::set_panel_size(plot, width=unit(4, "in"), height=unit(4, "in")),
       width = 10, height = 12,units = 'in', dpi = 300)
}

DimPlot_function (sce, group.by = "disease",outpath)
DimPlot_function (sce, group.by = "Accession",outpath)
DimPlot_function (sce, group.by = "orig.ident",outpath)
```

In [ ]:
```
library(clustree)
sce <- FindClusters(sce, resolution = seq(0.2,1,by=0.2))
p1=clustree(sce@meta.data, prefix = "RNA_snn_res.",node_colour = "percent.mt"
         node_colour_aggr = "mean", layout = "sugiyama")
p1
ggsave(file='./clustree.png',p1)
```

In [ ]:
```
DimPlot(sce, reduction = "umap", group.by = "RNA_snn_res.0.6",
        raster = FALSE, label = TRUE) +theme_dimplot
```

In [ ]:
```
Idents(object = sce) <- sce[["RNA_snn_res.0.6"]]
celltype <- c('0'= 'HEP','1'= 'HEP','2'= 'HEP','4'= 'HEP','7'= 'HEP','11'= 'H
              '17'= 'HEP','21'= 'HEP','23'= 'HEP','24'= 'HEP','25'= 'HEP',
                 '16'= 'EPI','8'= 'EN','15'= 'EN','22'= 'EN',
                 '13'= 'FC',
                 '3'= 'MC','6'= 'MC','10'= 'MC','19'= 'MC','20'= 'MC','26'= '
                 '5'='T_NK','12'='T_NK','16'='T_NK',
                 '9'= 'BC', '18'= 'BC',
                 '14'= 'PC','28'= 'Doubled','29'= 'Doubled'
                   )

sce <- RenameIdents(sce, celltype)
levels(sce)
sce[["Major_cell_type"]] <- Idents(object = sce)
levels_celltype <- c('HEP','EPI','EN','FC','MC','T_NK','BC','PC','Doubled')
sce$Major_cell_type <- factor(sce$Major_cell_type, levels = levels_celltype,
levels(sce)
```

In [ ]:
```
genes = c('APOA2','APOB','ALB','ALDOB',#malignant cells --MC
            "KRT19",'ANXA4',#normal epithelial cells--EPI
           'PECAM1','VWF',#ENDO
           'MRC1',"CD163",#Kupffer Cell
          'ACTA2',"COL1A1", #fibroblast
           "CD3D", "CD3E",#T cell
           "NKG7", #NK cell
           "CD79A", "MS4A1",
          "MKI67"
         )

plot4=DotPlot(sce, features = unique(genes),cluster.idents=TRUE)+
    theme_bw()+
    theme(panel.grid = element_blank(),
        axis.text.x=element_text(colour="black",size=20,hjust = 1,vjust=0.5,a
          axis.text.y=element_text(colour="black",size=20),
          legend.text=element_text(colour="black", size=20),
          legend.title=element_text(colour="black", size=20),
          axis.line=element_line(colour="black"),
```

```
            panel.background=element_rect(fill="white"),
            panel.border=element_rect(fill=NA,color="black", linewidth=2, linet
      labs(x=NULL,y=NULL)+guides(size=guide_legend(order=3))+
      scale_color_gradientn(values = seq(0,1,0.2),colours = c('#330066','#33669
plot4
ggsave(file=paste0("./rename.maker.expression.pdf",
                   sep=""),
       egg::set_panel_size(plot4, width=unit(4, "in"), height=unit(3, "in")),
       width = 10, height = 10,units = 'in', dpi = 300)
```

In [ ]:

```
DimPlot_function (sce, group.by = "Major_cell_type",outpath)
```

In [ ]:

```
cell_ratio <- function(sce_hep, group_var) {
    if (!group_var %in% colnames(sce_hep@meta.data)) {
        stop(paste("Metadata column", group_var, "not found in the Seurat obj
    }

    Idents(sce_hep) <- group_va
    clusterratio <- prop.table(table(Idents(sce_hep), sce_hep@meta.data[["dis
    clusterratio <- as.data.frame(clusterratio)
    colnames(clusterratio) <- c("Cluster", "Sample", "Freq")

    p2 <- ggplot(clusterratio) +
        geom_bar(aes(x = Cluster, y = Freq, fill = Sample),
                 stat = "identity", width = 0.7, color = "black") +
        theme_classic() +
        RotatedAxis() +
        labs(x = "Cluster", y = "Ratio") +
        theme(panel.border = element_rect(fill = NA, color = "black", linewid
    ggsave(paste0("./", group_var, "_cluster_ratio.pdf"), p2, width = 10, hei
}
```

In [ ]:

```
cell_ratio(sce,'Major_cell_type')
```

In [ ]:

```
outpath <- './hep'
setwd(outpath)
```

In [ ]:

```
sce_hep <- subset(sce,cell_type == 'HEP')
sce_hep <- Harmony_Function(sce_hep, group.by = "orig.ident",
                            lambda = 1,theta =2,dims.use = 1:50)
DimPlot(sce_hep, reduction = "umap", group.by = "disease", raster = FALSE, la
DimPlot(sce_hep, reduction = "umap", group.by = "Accession", raster = FALSE,
```

In [ ]:

```
DimPlot_function (sce_hep, group.by = "orig.ident",outpath)
DimPlot_function (sce_hep, group.by = "disease",outpath)
```

In [ ]:

```
library(clustree)
sce_hep <- FindClusters(sce_hep, resolution = seq(0.2,1.0,by=0.2))
p1=clustree(sce_hep@meta.data, prefix = "RNA_snn_res.",node_colour = "percent
         node_colour_aggr = "mean", layout = "sugiyama")
p1
ggsave(file='./clustree.png',p1)
```

In [ ]:
```
DimPlot(sce_hep, reduction = "umap", group.by = "RNA_snn_res.1", raster = FAL
```

In [ ]:
```
p1=FeaturePlot(sce_hep, features = c('APOA2','APOB','ALB','ALDOB','MKI67'),
               order = TRUE)
p1
ggsave(file='./hep_FeaturePlot.png',p1,width = 16, height = 16)
```

In [ ]:
```
Idents(object = sce_hep) <- sce_hep[["RNA_snn_res.1"]]
subcelltype <- c('0'= 'Tumor Cells','1'= 'Tumor Cells','2'= 'Tumor Cells',
                 '3'= 'Tumor Cells','4'= 'Tumor Cells','5'= 'Tumor Cells',
                 '7'= 'Tumor Cells','8'= 'Tumor Cells','10'= 'Tumor Cells',
                 '12'= 'Tumor Cells', '13'= 'Tumor Cells',
                 '6'= 'Hepatocytes','9'= 'Hepatocytes','11'= 'Hepatocytes',
                '14'= 'Hepatocytes')
sce_hep <- RenameIdents(sce_hep, subcelltype)
levels(sce_hep)
sce_hep[["sub_cell_type"]] <- Idents(object = sce_hep)
levels_subcelltype <- c('Hepatocytes','Tumor Cells')
sce_hep$sub_cell_type <- factor(sce_hep$sub_cell_type, levels = levels_subcel
levels(sce_hep)
```

In [ ]:
```
DimPlot_function (sce_hep, group.by = "sub_cell_type",outpath)
```

In [ ]:
```
genes = c('TRMT6','TRMT61A',
         'TRMT61B','TRMT10C','BMT2',
         'YTHDF1','YTHDF2','YTHDF3','YTHDC1',
         'ALKBH1','ALKBH3','FTO')

plot4=DotPlot(sce_hep, group.by='disease',
              features = unique(genes),cluster.idents=TRUE)+
    theme_bw()+
    theme(panel.grid = element_blank(),
        axis.text.x=element_text(colour="black",size=20,hjust = 1,vjust=0.5,a
          axis.text.y=element_text(colour="black",size=20),
          legend.text=element_text(colour="black", size=20),
          legend.title=element_text(colour="black", size=20),
          axis.line=element_line(colour="black"),
          panel.background=element_rect(fill="white"),
          panel.border=element_rect(fill=NA,color="black", linewidth=2, linet
    labs(x=NULL,y=NULL)+guides(size=guide_legend(order=3))+
    scale_color_gradientn(values = seq(0,1,0.2),colours = c('#330066','#33669
plot4
ggsave(file=paste0("./HEP_disease_expression.pdf",sep=""),
       egg::set_panel_size(plot4, width=unit(3, "in"), height=unit(2, "in")),
       width = 10, height = 10,units = 'in', dpi = 300)
```

In [ ]:
```
genes = c('TRMT6','TRMT61A',
         'TRMT61B','TRMT10C','BMT2',
         'YTHDF1','YTHDF2','YTHDF3','YTHDC1',
          'ALKBH1','ALKBH3','FTO')

plot4=DotPlot(sce_hep, group.by='sub_cell_type',
              features = unique(genes),cluster.idents=TRUE)+
    theme_bw()+
    theme(panel.grid = element_blank(),
        axis.text.x=element_text(colour="black",size=20,hjust = 1,vjust=0.5,a
```

```
            axis.text.y=element_text(colour="black",size=20),
            legend.text=element_text(colour="black", size=20),
            legend.title=element_text(colour="black", size=20),
            axis.line=element_line(colour="black"),
            panel.background=element_rect(fill="white"),
            panel.border=element_rect(fill=NA,color="black", linewidth=2, linet
    labs(x=NULL,y=NULL)+guides(size=guide_legend(order=3))+
    scale_color_gradientn(values = seq(0,1,0.2),colours = c('#330066','#33669
plot4
ggsave(file=paste0("./HEP_sub_cell_type_expression.pdf",sep=""),
       egg::set_panel_size(plot4, width=unit(3, "in"), height=unit(2, "in")),
       width = 10, height = 10,units = 'in', dpi = 300)
```

In [ ]:

```
genes = c('TRMT6')

plot4=DotPlot(sce_hep, group.by='sub_cell_type',
              features = unique(genes),cluster.idents=TRUE)+
    theme_bw()+
    theme(panel.grid = element_blank(),
        axis.text.x=element_text(colour="black",size=20,hjust = 1,vjust=0.5,a
          axis.text.y=element_text(colour="black",size=20),
          legend.text=element_text(colour="black", size=20),
          legend.title=element_text(colour="black", size=20),
          axis.line=element_line(colour="black"),
          panel.background=element_rect(fill="white"),
          panel.border=element_rect(fill=NA,color="black", linewidth=2, linet
    labs(x=NULL,y=NULL)+guides(size=guide_legend(order=3))+
    scale_color_gradientn(values = seq(0,1,0.2),colours = c('#330066','#33669
plot4
ggsave(file=paste0("./HEP_sub_cell_type_TRMT6.pdf",sep=""),
       egg::set_panel_size(plot4, width=unit(1, "in"), height=unit(2, "in")),
       width = 6, height = 6,units = 'in', dpi = 300)
```

In [ ]:

```
genes = c('TRMT6')

plot4=DotPlot(sce_hep, group.by='disease',
              features = unique(genes),cluster.idents=TRUE)+
    theme_bw()+
    theme(panel.grid = element_blank(),
        axis.text.x=element_text(colour="black",size=20,hjust = 1,vjust=0.5,a
          axis.text.y=element_text(colour="black",size=20),
          legend.text=element_text(colour="black", size=20),
          legend.title=element_text(colour="black", size=20),
          axis.line=element_line(colour="black"),
          panel.background=element_rect(fill="white"),
          panel.border=element_rect(fill=NA,color="black", linewidth=2, linet
    labs(x=NULL,y=NULL)+guides(size=guide_legend(order=3))+
    scale_color_gradientn(values = seq(0,1,0.2),colours = c('#330066','#33669
plot4
ggsave(file=paste0("./HEP_disease_TRMT6.pdf",sep=""),
       egg::set_panel_size(plot4, width=unit(1, "in"), height=unit(2, "in")),
       width = 6, height = 6,units = 'in', dpi = 300)
```

In [ ]:

```
p1=FeaturePlot(sce_hep, features = c('APOA2','APOB','ALB','ALDOB','MKI67'),
               order = TRUE)
p1
ggsave(file="./hep.Feature.pdf",p1)
```

In [ ]:

```
p1=FeaturePlot(sce_hep, features = c('TRMT6','MKI67','MKI67'),
               order = TRUE)
p1
ggsave(file="./TRMT6.Feature.pdf",p1)
```

In [ ]:

```
cell_ratio <- function(sce_hep, group_var) {

    if (!group_var %in% colnames(sce_hep@meta.data)) {
        stop(paste("Metadata column", group_var, "not found in the Seurat obj
    }

    clusterratio <- prop.table(table(Idents(sce_hep), sce_hep@meta.data[["ori
    clusterratio <- as.data.frame(clusterratio)
    colnames(clusterratio) <- c("Cluster", "Sample", "Freq")

    p2 <- ggplot(clusterratio) +
        geom_bar(aes(x = Cluster, y = Freq, fill = Sample),
                 stat = "identity", width = 0.7, color = "black") +

        RotatedAxis() +
        labs(x = "Cluster", y = "Ratio") +
        theme(panel.border = element_rect(fill = NA, color = "black", linewid

    ggsave(paste0("./", group_var, "_cluster_ratio.pdf"), p2, width = 10, hei
}

cell_ratio(sce_hep,'disease')
```

In [ ]:

```
cols_disease=c('Hepatocytes'='#0090CC', 'Tumor Cells'='#fa94a7')
gene_list = c('TFB1M', 'NSUN3', 'TRMT61A', 'LIN9', 'SNRPE', 'TFB2M',
              'NSUN6', 'TRDMT1', 'METTL5', 'LCMT2', 'SNRPB', 'RBM15',
              'TYW3', 'NSUN4', 'ZCCHC4', 'MEPCE', 'SNRPG', 'THUMPD3',
              'SNRPD2', 'FBL', 'TARBP1', 'GTPBP3', 'MTO1', 'RBM15B',
              'WDR6', 'FTSJ1', 'SNRPD3', 'SNRPF', 'METTL1', 'FBLL1',
              'FTSJ3', 'DIMT1', 'NSUN2', 'METTL6', 'METTL3', 'TRMT12',
              'BCDIN3D', 'THADA', 'TRMT61B', 'THUMPD2', 'TRMT112', 'HENMT1',
              'METTL2B', 'METTL2A', 'TRMT13', 'TRMT44', 'TRMT10A', 'MRM2', 'T
#RNA_Methylation%20(GO_0001510)_from_GO_Biological_Process_2025
score = Seurat::AddModuleScore(sce_hep, features = list(gene_list), name = 'R
p1=VlnPlot(score, features = 'RNA_methylation1',group.by='sub_cell_type',
        cols=cols_disease,pt.size=0,flip =T,add.noise = T,adjust = 1)+theme_d
  stat_compare_means()
p1
ggsave(file='./RNA_Methylation_subcelltype.pdf', p1)
```

In [ ]:

```
gene_list = c('METTL3', 'METTL14', 'WTAP', 'RBM15', 'ZC3H13',
              'VIRMA', 'KIAA1429', 'METTL4', 'METTL5', 'METTL16',
              'FTO', 'ALKBH3', 'ALKBH5', 'YTHDF1', 'YTHDF2', 'YTHDF3',
              'YTHDC1', 'YTHDC2', 'IGFBP1',
              'IGFBP2', 'IGFBP3', 'HNRNPC', 'HNRNPG', 'HNRNPA2B1')
#M6A_Methylation%
score = Seurat::AddModuleScore(sce_hep, features = list(gene_list), name = 'M
p1=VlnPlot(score, features = 'M6A_methylation1',group.by='sub_cell_type',
        cols=cols_disease,pt.size=0,flip =T,add.noise = T,adjust = 1)+theme_d
  stat_compare_means()
p1
ggsave(file='./M6A_methy_subcelltype.pdf', p1)
```
