# Supplementary material for: TRMT6‐Mediated m1A Modification of CDK9 mRNA is a Dual‐Pronged Pathogenic Driver for HBV‐Related Hepatocellular Carcinoma
Source: Adv Sci (Weinh). 2026 Apr 20;13(39):e14172. doi: 10.1002/advs.202514172 (PMC13334973; doi:10.1002/advs.202514172)
Supplement: Supplementary file 2 — Supporting File 2: advs75266‐sup‐0002‐Data.zip. [file ADVS-13-e14172-s001.zip › Supplementary Figures WB Original Stripps.pptx]

## Slide 1
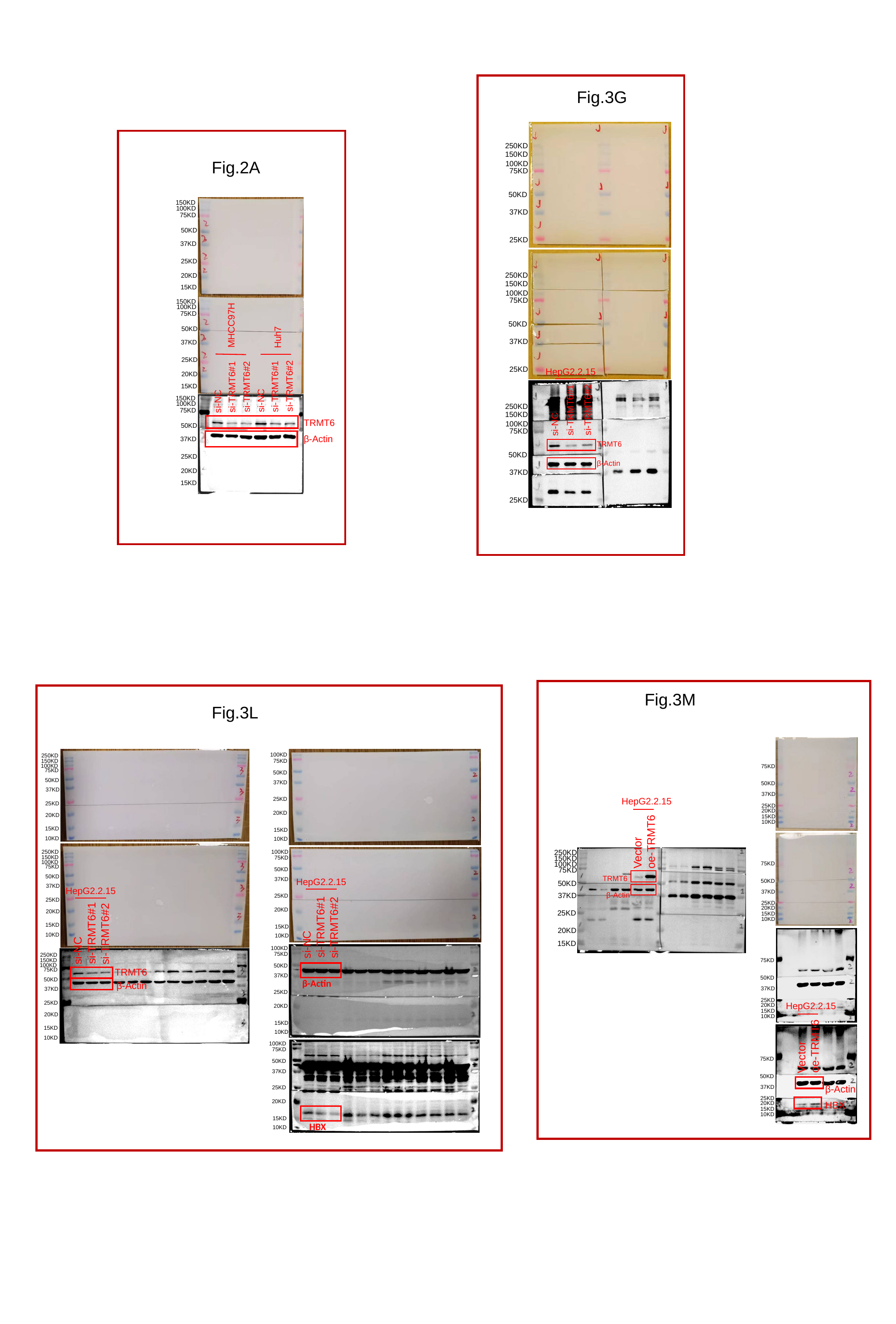

Fig.3G
250KD
150KD
Fig.2A
100KD
75KD
50KD
150KD
100KD
75KD
50KD
37KD
25KD
20KD
15KD
150KD
100KD
75KD
MHCC97H
50KD
Huh7
37KD
25KD
20KD
si-TRMT6#2
si-TRMT6#1
si-TRMT6#2
si-TRMT6#1
15KD
150KD
si-NC
si-NC
100KD
75KD
TRMT6
50KD
β-Actin
37KD
25KD
20KD
15KD
37KD
25KD
250KD
150KD
100KD
75KD
50KD
37KD
25KD
HepG2.2.15
250KD
si-TRMT6#2
si-TRMT6#1
150KD
si-NC
100KD
75KD
TRMT6
50KD
β-Actin
37KD
25KD
Fig.3M
Fig.3L
75KD
50KD
37KD
25KD
20KD
15KD
10KD
75KD
50KD
37KD
25KD
20KD
15KD
10KD
75KD
50KD
37KD
25KD
20KD
15KD
10KD
oe-TRMT6
Vector
75KD
50KD
β-Actin
37KD
25KD
HBX
20KD
15KD
10KD
HepG2.2.15
100KD
75KD
50KD
37KD
25KD
20KD
15KD
10KD
100KD
75KD
50KD
37KD
25KD
20KD
si-TRMT6#1
si-TRMT6#2
15KD
10KD
si-NC
100KD
75KD
50KD
37KD
β-Actin
25KD
20KD
15KD
10KD
100KD
75KD
50KD
37KD
25KD
20KD
15KD
HBX
10KD
250KD
150KD
100KD
75KD
50KD
37KD
25KD
20KD
15KD
10KD
250KD
150KD
100KD
75KD
50KD
37KD
25KD
20KD
15KD
si-TRMT6#1
si-TRMT6#2
10KD
si-NC
250KD
150KD
100KD
TRMT6
75KD
50KD
β-Actin
37KD
25KD
20KD
15KD
10KD
HepG2.2.15
HepG2.2.15
HepG2.2.15
oe-TRMT6
Vector
250KD
150KD
100KD
75KD
TRMT6
50KD
β-Actin
37KD
25KD
20KD
15KD

## Slide 2
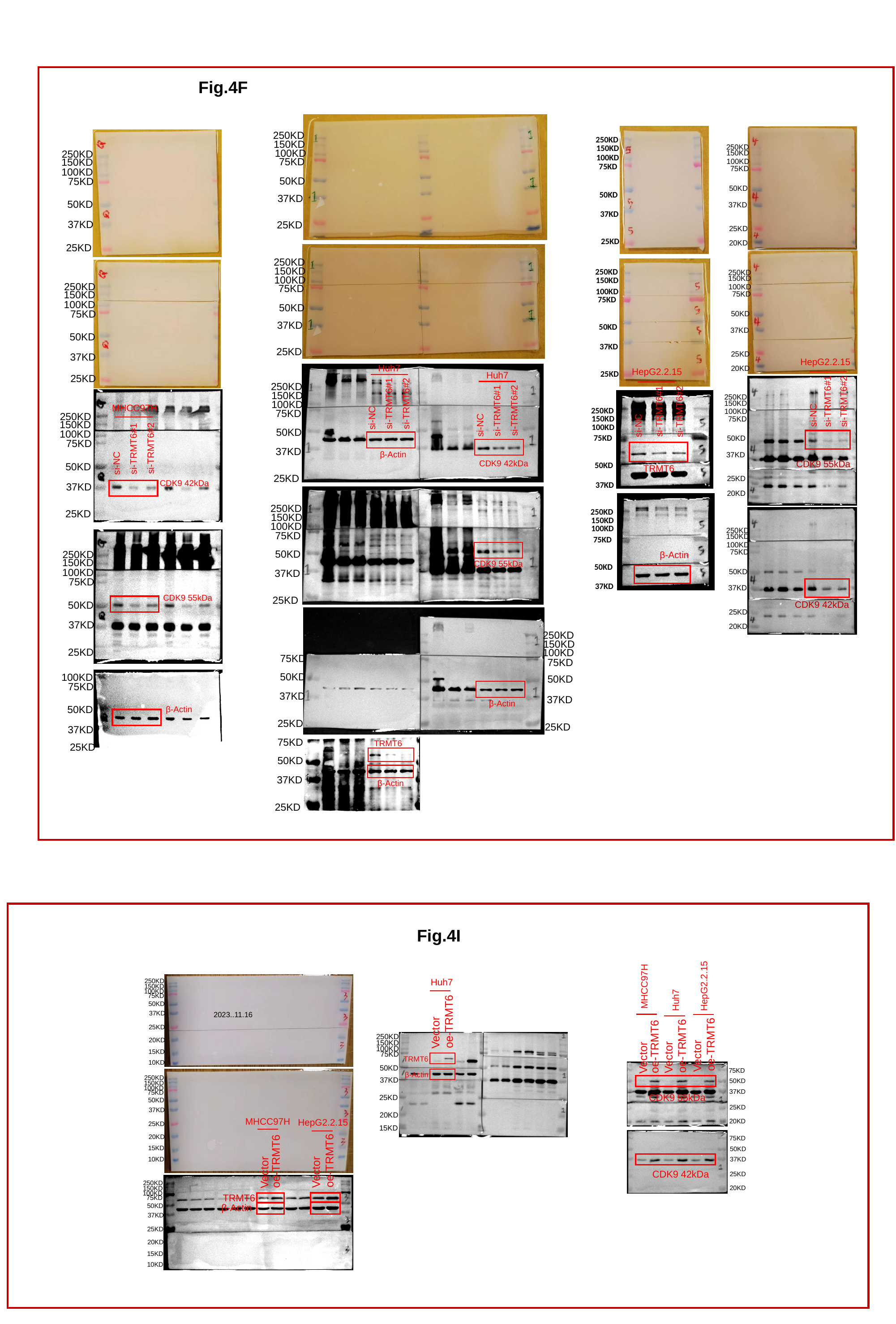

Fig.4F
250KD
150KD
100KD
75KD
50KD
37KD
25KD
250KD
150KD
100KD
75KD
50KD
37KD
25KD
250KD
150KD
100KD
75KD
50KD
37KD
25KD
250KD
250KD
150KD
Huh7
150KD
100KD
75KD
100KD
75KD
50KD
50KD
37KD
37KD
25KD
20KD
25KD
250KD
150KD
100KD
75KD
50KD
37KD
25KD
250KD
150KD
250KD
100KD
150KD
75KD
100KD
75KD
50KD
37KD
50KD
25KD
37KD
HepG2.2.15
Huh7
20KD
HepG2.2.15
250KD
150KD
100KD
75KD
50KD
37KD
CDK9 42kDa
25KD
Huh7
25KD
250KD
250KD
150KD
100KD
75KD
50KD
TRMT6
37KD
si-TRMT6#1
si-TRMT6#2
si-TRMT6#2
si-TRMT6#1
150KD
MHCC97H
si-TRMT6#2
si-TRMT6#1
si-TRMT6#1
si-TRMT6#2
100KD
si-NC
250KD
si-NC
75KD
150KD
si-NC
si-NC
100KD
50KD
75KD
si-TRMT6#2
si-TRMT6#1
β-Actin
37KD
si-NC
CDK9 55kDa
50KD
25KD
CDK9 42kDa
37KD
20KD
250KD
150KD
100KD
75KD
50KD
CDK9 55kDa
37KD
25KD
250KD
150KD
100KD
75KD
β-Actin
50KD
37KD
25KD
250KD
150KD
100KD
75KD
250KD
150KD
100KD
50KD
75KD
37KD
CDK9 55kDa
50KD
CDK9 42kDa
25KD
250KD
150KD
100KD
75KD
75KD
50KD
50KD
37KD
37KD
25KD
25KD
37KD
20KD
25KD
100KD
75KD
50KD
β-Actin
37KD
25KD
β-Actin
75KD
TRMT6
50KD
37KD
β-Actin
25KD
Fig.4I
HepG2.2.15
MHCC97H
Huh7
oe-TRMT6
oe-TRMT6
oe-TRMT6
Vector
Vector
Vector
75KD
50KD
37KD
CDK9 55kDa
25KD
20KD
75KD
50KD
37KD
CDK9 42kDa
25KD
20KD
Huh7
oe-TRMT6
Vector
250KD
150KD
100KD
75KD
TRMT6
50KD
β-Actin
37KD
25KD
20KD
15KD
250KD
150KD
100KD
75KD
50KD
37KD
2023..11.16
25KD
20KD
15KD
10KD
250KD
150KD
100KD
75KD
50KD
37KD
MHCC97H
HepG2.2.15
25KD
20KD
15KD
oe-TRMT6
oe-TRMT6
10KD
Vector
Vector
250KD
150KD
100KD
TRMT6
75KD
50KD
β-Actin
37KD
25KD
20KD
15KD
10KD

## Slide 3
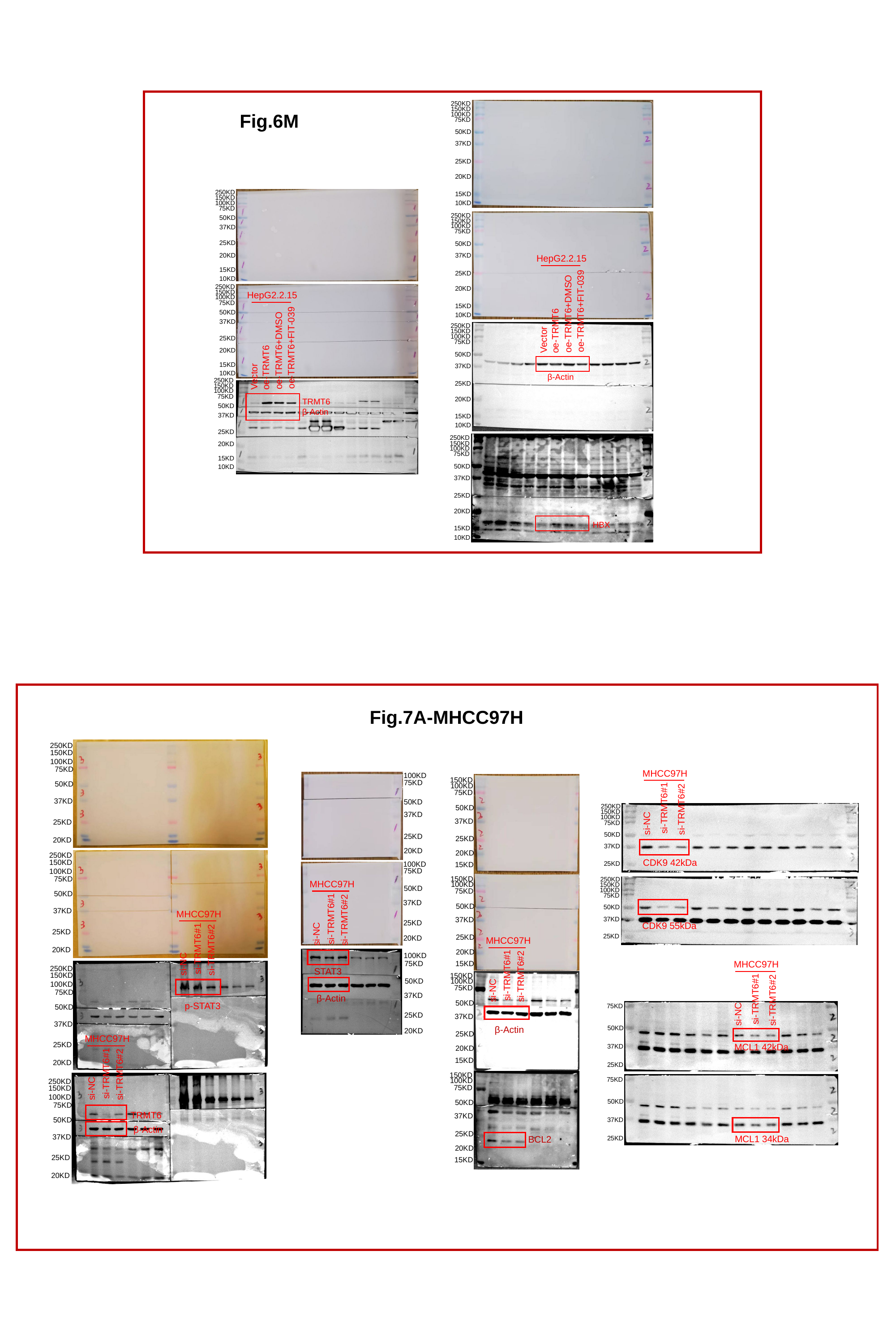

250KD
150KD
Fig.6M
100KD
75KD
50KD
37KD
25KD
20KD
250KD
15KD
150KD
100KD
10KD
75KD
250KD
50KD
150KD
100KD
37KD
75KD
25KD
50KD
20KD
37KD
HepG2.2.15
15KD
25KD
10KD
250KD
20KD
150KD
HepG2.2.15
100KD
75KD
15KD
oe-TRMT6+FIT-039
50KD
oe-TRMT6+DMSO
10KD
37KD
250KD
oe-TRMT6
150KD
100KD
25KD
Vector
75KD
oe-TRMT6+FIT-039
oe-TRMT6+DMSO
20KD
50KD
15KD
37KD
oe-TRMT6
10KD
Vector
β-Actin
250KD
25KD
150KD
100KD
75KD
20KD
TRMT6
50KD
β-Actin
37KD
15KD
10KD
25KD
250KD
150KD
20KD
100KD
75KD
15KD
50KD
10KD
37KD
25KD
20KD
HBX
15KD
10KD
Fig.7A-MHCC97H
250KD
150KD
100KD
75KD
MHCC97H
100KD
150KD
75KD
50KD
100KD
75KD
37KD
50KD
250KD
si-TRMT6#1
50KD
si-TRMT6#2
150KD
37KD
100KD
37KD
25KD
si-NC
75KD
50KD
25KD
25KD
20KD
37KD
20KD
20KD
250KD
150KD
100KD
75KD
50KD
37KD
25KD
20KD
CDK9 42kDa
25KD
100KD
15KD
75KD
150KD
250KD
MHCC97H
100KD
150KD
50KD
100KD
75KD
75KD
37KD
50KD
50KD
MHCC97H
si-TRMT6#1
si-TRMT6#2
37KD
37KD
25KD
CDK9 55kDa
si-NC
25KD
25KD
20KD
MHCC97H
si-TRMT6#1
si-TRMT6#2
20KD
100KD
si-NC
MHCC97H
15KD
75KD
250KD
150KD
100KD
75KD
p-STAT3
50KD
37KD
25KD
20KD
STAT3
si-TRMT6#1
si-TRMT6#2
150KD
50KD
100KD
75KD
si-NC
37KD
β-Actin
si-TRMT6#1
si-TRMT6#2
50KD
75KD
si-NC
25KD
37KD
β-Actin
50KD
20KD
25KD
MHCC97H
MCL1 42kDa
37KD
20KD
15KD
25KD
si-TRMT6#1
si-TRMT6#2
150KD
75KD
100KD
250KD
75KD
si-NC
150KD
100KD
50KD
50KD
75KD
TRMT6
37KD
50KD
37KD
β-Actin
25KD
37KD
MCL1 34kDa
BCL2
25KD
20KD
25KD
15KD
20KD

## Slide 4
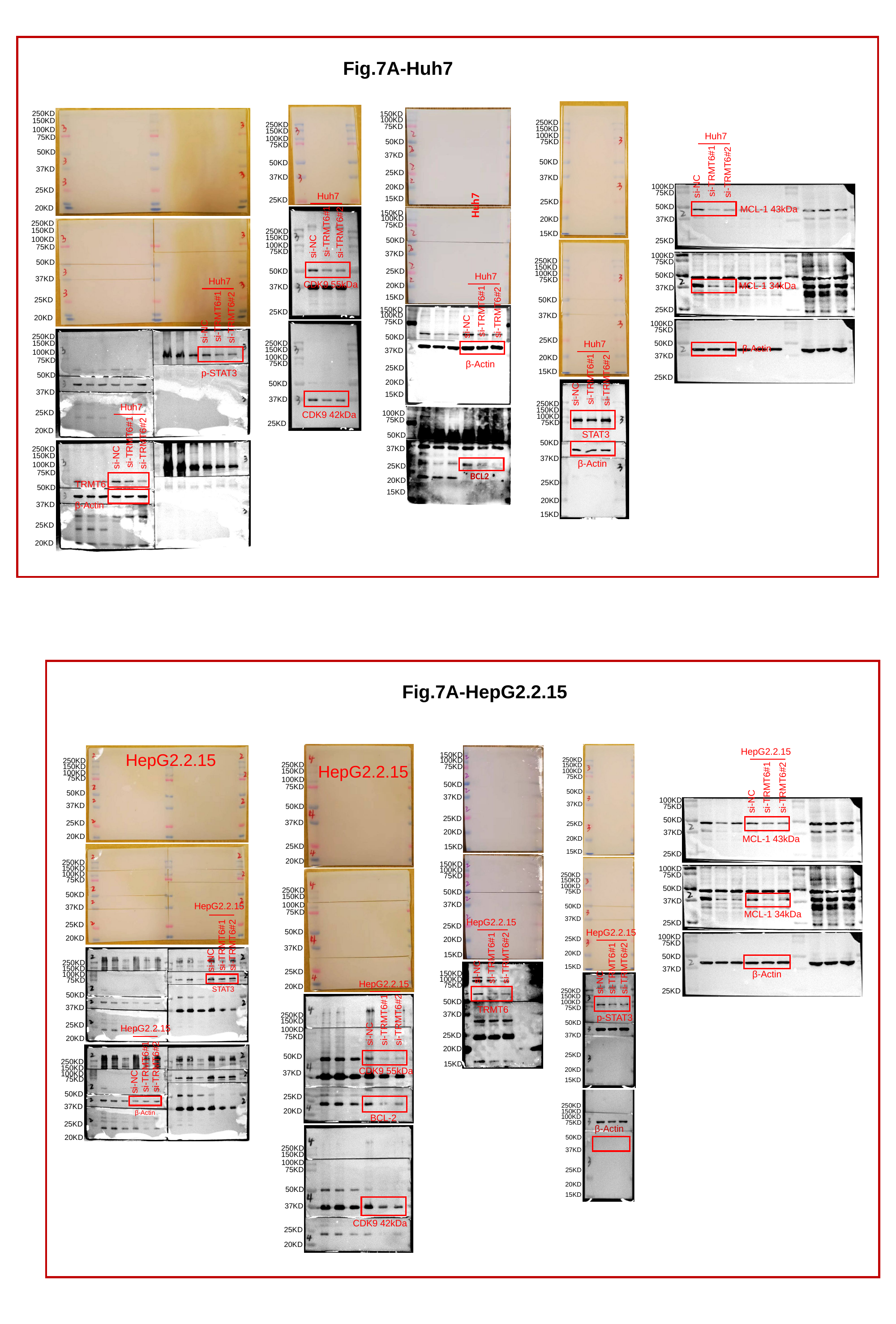

Fig.7A-Huh7
250KD
150KD
100KD
75KD
50KD
37KD
25KD
20KD
250KD
150KD
100KD
75KD
50KD
37KD
Huh7
25KD
si-TRMT6#1
si-TRMT6#2
20KD
si-NC
250KD
150KD
100KD
75KD
p-STAT3
50KD
37KD
Huh7
25KD
20KD
si-TRMT6#1
si-TRMT6#2
250KD
150KD
si-NC
100KD
75KD
TRMT6
50KD
β-Actin
37KD
25KD
20KD
150KD
100KD
250KD
250KD
75KD
150KD
150KD
Huh7
100KD
100KD
50KD
75KD
75KD
37KD
50KD
50KD
si-TRMT6#1
si-TRMT6#2
25KD
37KD
37KD
si-NC
100KD
20KD
75KD
Huh7
15KD
25KD
25KD
Huh7
50KD
MCL-1 43kDa
150KD
100KD
37KD
20KD
75KD
si-TRMT6#1
250KD
si-TRMT6#2
15KD
150KD
50KD
25KD
100KD
si-NC
75KD
37KD
100KD
250KD
75KD
150KD
25KD
50KD
100KD
Huh7
50KD
75KD
CDK9 55kDa
MCL-1 34kDa
20KD
37KD
37KD
15KD
50KD
150KD
25KD
si-TRMT6#1
si-TRMT6#2
25KD
100KD
37KD
75KD
100KD
si-NC
75KD
50KD
25KD
Huh7
50KD
250KD
β-Actin
150KD
37KD
37KD
100KD
20KD
β-Actin
75KD
25KD
15KD
25KD
si-TRMT6#1
si-TRMT6#2
20KD
50KD
si-NC
15KD
37KD
250KD
150KD
100KD
CDK9 42kDa
100KD
75KD
75KD
25KD
STAT3
50KD
50KD
37KD
37KD
β-Actin
25KD
BCL2
20KD
25KD
15KD
20KD
15KD
Fig.7A-HepG2.2.15
HepG2.2.15
HepG2.2.15
150KD
250KD
100KD
250KD
250KD
HepG2.2.15
150KD
75KD
150KD
150KD
100KD
100KD
75KD
75KD
100KD
50KD
si-TRMT6#1
75KD
si-TRMT6#2
50KD
50KD
37KD
100KD
si-NC
37KD
37KD
50KD
75KD
25KD
50KD
37KD
25KD
25KD
20KD
37KD
20KD
MCL-1 43kDa
20KD
25KD
15KD
15KD
25KD
20KD
250KD
150KD
150KD
100KD
100KD
100KD
75KD
250KD
75KD
75KD
150KD
100KD
50KD
250KD
75KD
50KD
50KD
150KD
37KD
37KD
100KD
HepG2.2.15
50KD
37KD
75KD
MCL-1 34kDa
37KD
HepG2.2.15
25KD
25KD
25KD
HepG2.2.15
50KD
100KD
20KD
25KD
20KD
75KD
si-TRMT6#2
si-TRMT6#1
37KD
20KD
15KD
50KD
si-TRMT6#2
si-TRMT6#1
si-NC
250KD
15KD
si-TRMT6#2
si-TRMT6#1
150KD
37KD
si-NC
25KD
β-Actin
150KD
100KD
100KD
75KD
si-NC
HepG2.2.15
75KD
20KD
STAT3
25KD
250KD
50KD
150KD
50KD
100KD
37KD
TRMT6
75KD
37KD
250KD
p-STAT3
si-TRMT6#2
si-TRMT6#1
150KD
50KD
25KD
HepG2.2.15
100KD
si-NC
25KD
37KD
75KD
20KD
20KD
25KD
50KD
250KD
15KD
si-TRMT6#2
si-TRMT6#1
150KD
CDK9 55kDa
20KD
37KD
100KD
75KD
15KD
si-NC
50KD
25KD
250KD
37KD
20KD
150KD
β-Actin
BCL-2
100KD
75KD
25KD
β-Actin
20KD
50KD
250KD
37KD
150KD
100KD
75KD
25KD
20KD
50KD
15KD
37KD
CDK9 42kDa
25KD
20KD

## Slide 5
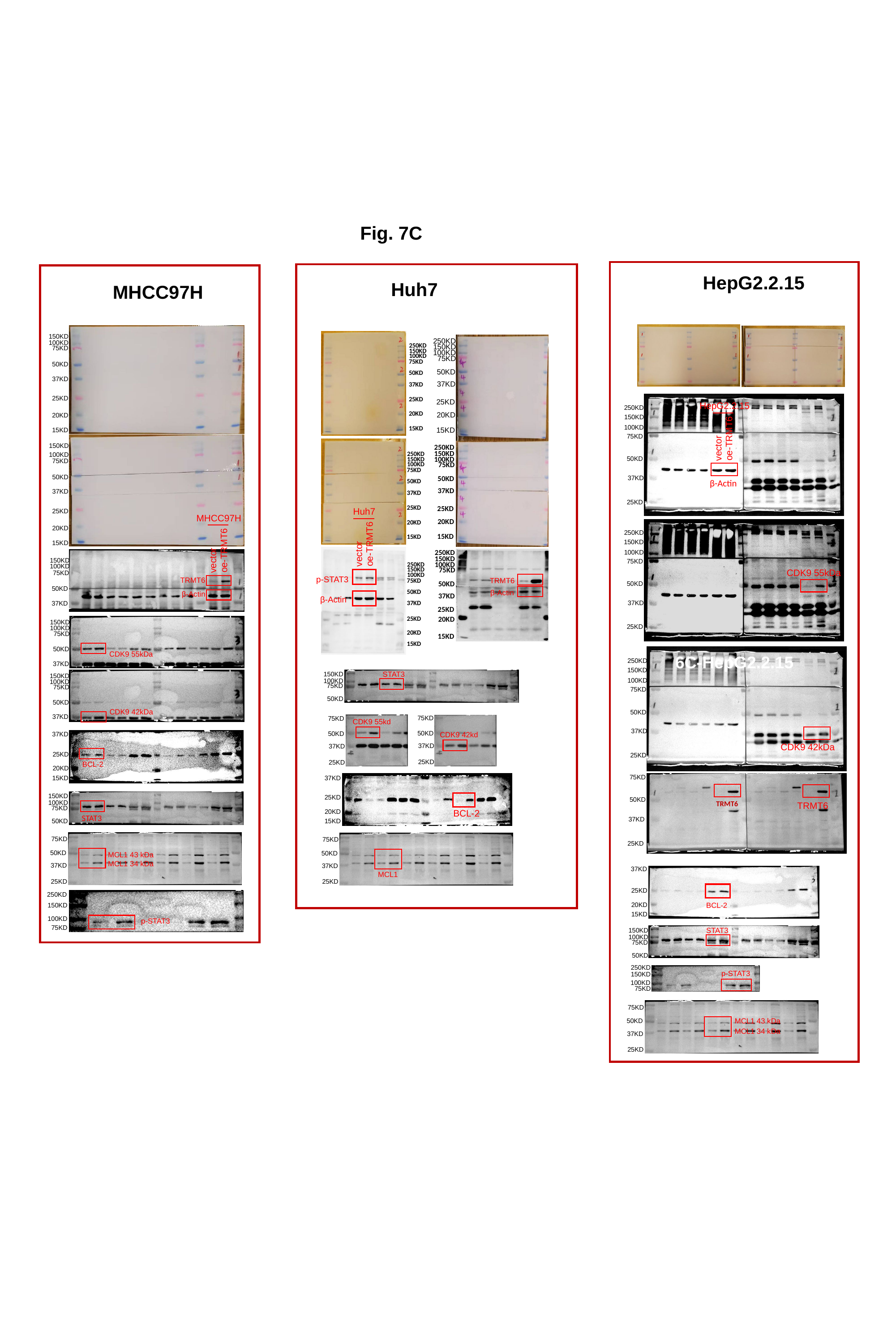

Fig. 7C
6C-HepG2.2.15
HepG2.2.15
Huh7
MHCC97H
150KD
250KD
100KD
250KD
150KD
75KD
150KD
100KD
100KD
75KD
75KD
50KD
50KD
50KD
37KD
37KD
37KD
25KD
25KD
25KD
HepG2.2.15
250KD
20KD
20KD
20KD
150KD
100KD
15KD
15KD
15KD
75KD
oe-TRMT6
150KD
vector
250KD
150KD
250KD
100KD
100KD
50KD
150KD
75KD
100KD
75KD
75KD
50KD
50KD
37KD
50KD
β-Actin
37KD
37KD
37KD
25KD
25KD
25KD
Huh7
25KD
MHCC97H
20KD
20KD
20KD
250KD
15KD
15KD
150KD
oe-TRMT6
15KD
oe-TRMT6
250KD
vector
100KD
150KD
vector
150KD
75KD
100KD
250KD
100KD
150KD
75KD
CDK9 55kDa
75KD
100KD
p-STAT3
TRMT6
TRMT6
75KD
50KD
50KD
50KD
50KD
β-Actin
β-Actin
37KD
β-Actin
37KD
37KD
37KD
25KD
25KD
20KD
150KD
25KD
100KD
20KD
75KD
15KD
15KD
50KD
CDK9 55kDa
250KD
37KD
150KD
STAT3
150KD
150KD
100KD
100KD
100KD
75KD
75KD
75KD
50KD
50KD
CDK9 42kDa
50KD
37KD
75KD
75KD
CDK9 55kd
37KD
50KD
50KD
37KD
CDK9 42kd
CDK9 42kDa
37KD
37KD
25KD
25KD
25KD
25KD
BCL-2
20KD
75KD
37KD
15KD
150KD
25KD
50KD
100KD
TRMT6
TRMT6
75KD
20KD
BCL-2
STAT3
37KD
15KD
50KD
75KD
75KD
25KD
50KD
50KD
MCL1 43 kDa
MCL1 34 kDa
37KD
37KD
37KD
MCL1
25KD
25KD
25KD
250KD
20KD
BCL-2
150KD
15KD
100KD
p-STAT3
75KD
STAT3
150KD
100KD
75KD
50KD
250KD
p-STAT3
150KD
100KD
75KD
75KD
MCL1 43 kDa
50KD
MCL1 34 kDa
37KD
25KD

## Slide 6
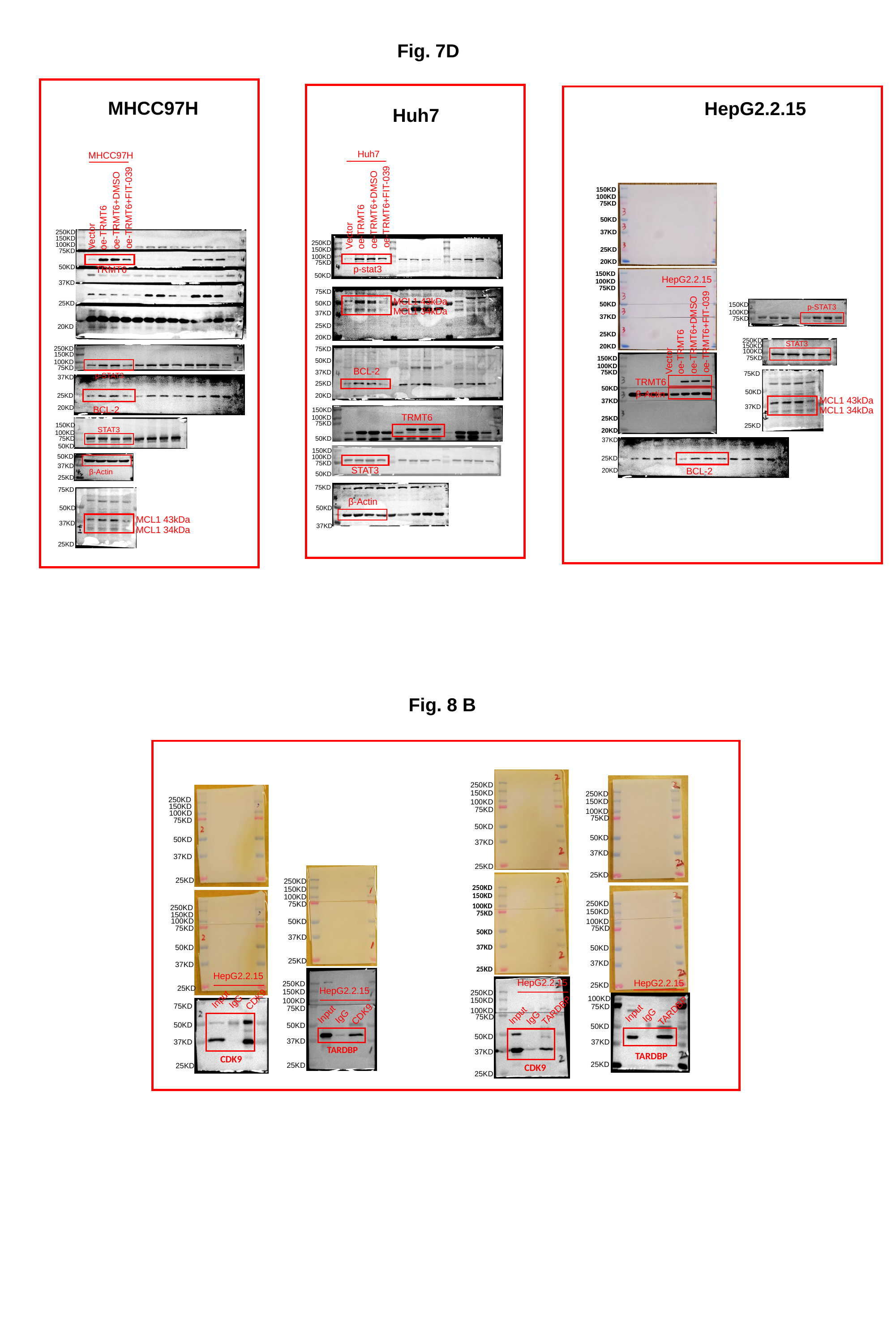

Fig. 7D
MHCC97H
HepG2.2.15
Huh7
Huh7
MHCC97H
150KD
100KD
75KD
oe-TRMT6+FIT-039
oe-TRMT6+FIT-039
oe-TRMT6+DMSO
oe-TRMT6+DMSO
50KD
oe-TRMT6
oe-TRMT6
37KD
250KD
Vector
Vector
150KD
250KD
100KD
25KD
150KD
75KD
100KD
20KD
75KD
50KD
p-stat3
TRMT6
150KD
50KD
HepG2.2.15
100KD
37KD
75KD
75KD
MCL1 43kDa
50KD
25KD
50KD
150KD
p-STAT3
MCL1 34kDa
100KD
37KD
37KD
75KD
25KD
20KD
oe-TRMT6+FIT-039
oe-TRMT6+DMSO
25KD
20KD
250KD
STAT3
150KD
20KD
250KD
75KD
oe-TRMT6
100KD
150KD
75KD
150KD
Vector
50KD
100KD
100KD
75KD
BCL-2
37KD
75KD
75KD
p-STAT3
37KD
TRMT6
25KD
50KD
50KD
β-Actin
25KD
20KD
MCL1 43kDa
37KD
37KD
20KD
BCL-2
MCL1 34kDa
150KD
TRMT6
100KD
25KD
75KD
150KD
25KD
STAT3
20KD
100KD
75KD
50KD
37KD
50KD
150KD
50KD
100KD
25KD
75KD
37KD
STAT3
BCL-2
20KD
β-Actin
50KD
25KD
75KD
75KD
β-Actin
50KD
50KD
MCL1 43kDa
37KD
37KD
MCL1 34kDa
25KD
Fig. 8 B
250KD
150KD
250KD
250KD
150KD
100KD
150KD
75KD
100KD
100KD
75KD
75KD
50KD
50KD
50KD
37KD
37KD
37KD
25KD
25KD
25KD
250KD
250KD
150KD
100KD
75KD
50KD
37KD
25KD
150KD
100KD
250KD
75KD
250KD
150KD
150KD
100KD
50KD
100KD
75KD
75KD
37KD
50KD
50KD
25KD
37KD
37KD
HepG2.2.15
HepG2.2.15
HepG2.2.15
250KD
25KD
25KD
HepG2.2.15
150KD
250KD
Input
CDK9
100KD
IgG
150KD
100KD
75KD
75KD
75KD
TARDBP
TARDBP
100KD
Input
Input
CDK9
IgG
Input
IgG
IgG
75KD
50KD
50KD
50KD
50KD
37KD
37KD
37KD
TARDBP
37KD
TARDBP
CDK9
25KD
25KD
25KD
CDK9
25KD

## Slide 7
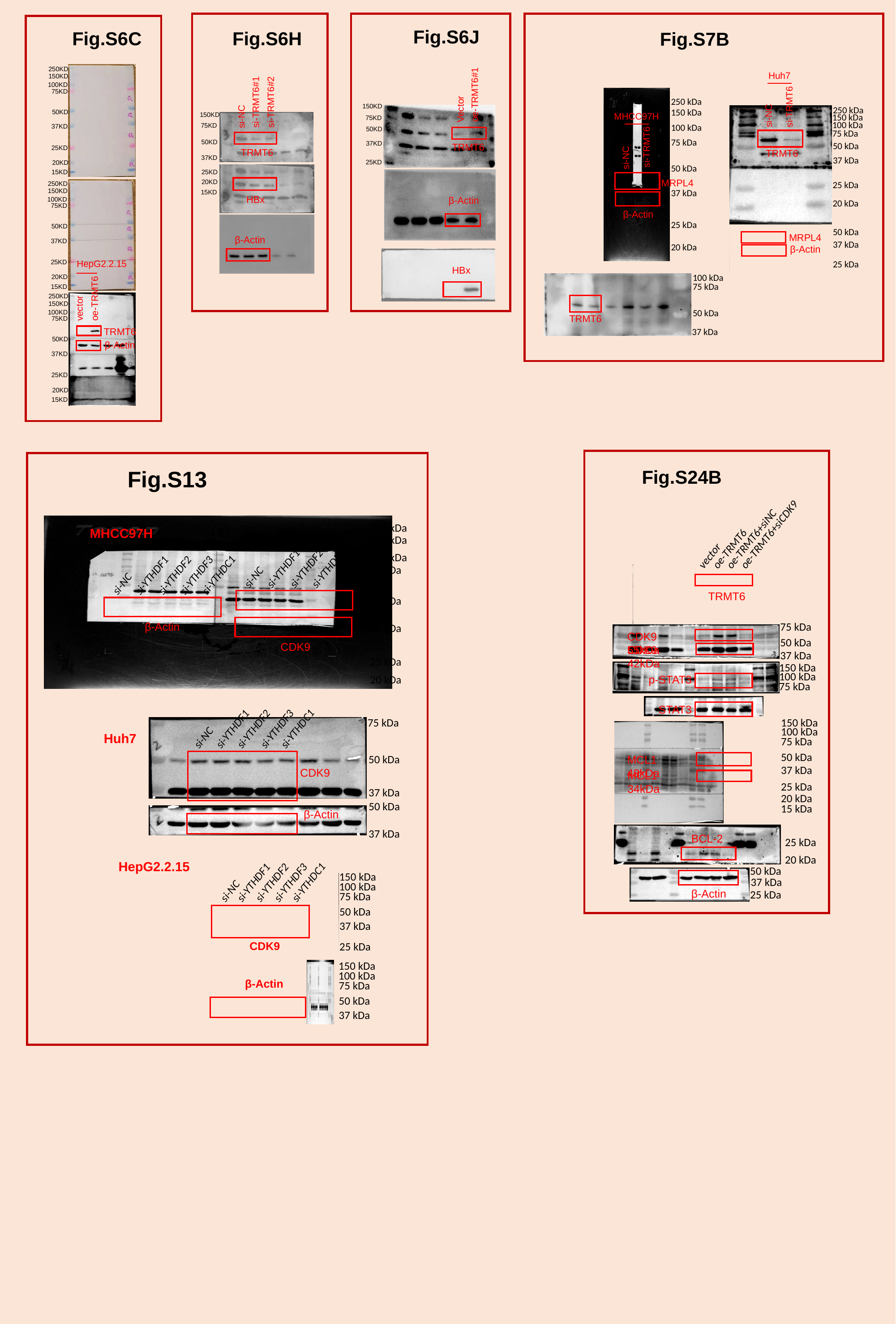

Fig.S6J
Fig.S6C
Fig.S6H
Fig.S7B
oe-TRMT6#1
150KD
Vector
75KD
50KD
37KD
TRMT6
25KD
β-Actin
HBx
250KD
Huh7
si-TRMT6
250 kDa
si-NC
150 kDa
100 kDa
75 kDa
50 kDa
TRMT6
37 kDa
25 kDa
20 kDa
50 kDa
MRPL4
37 kDa
β-Actin
25 kDa
150KD
si-TRMT6#2
si-TRMT6#1
si-NC
75KD
50KD
TRMT6
37KD
25KD
20KD
15KD
HBx
β-Actin
150KD
100KD
75KD
250 kDa
150 kDa
MHCC97H
100 kDa
75 kDa
si-TRMT6
si-NC
50 kDa
MRPL4
37 kDa
β-Actin
25 kDa
20 kDa
50KD
37KD
25KD
20KD
15KD
250KD
150KD
100KD
75KD
50KD
37KD
HepG2.2.15
25KD
100 kDa
20KD
75 kDa
15KD
250KD
oe-TRMT6
150KD
vector
50 kDa
100KD
TRMT6
75KD
TRMT6
37 kDa
50KD
β-Actin
37KD
25KD
20KD
15KD
Fig.S13
Fig.S24B
oe-TRMT6+siCDK9
oe-TRMT6+siNC
oe-TRMT6
vector
250 kDa
MHCC97H
150 kDa
si-YTHDF1
si-YTHDF2
si-YTHDF3
si-YTHDC1
si-NC
si-YTHDF1
si-YTHDF2
si-YTHDF3
si-YTHDC1
si-NC
100 kDa
75 kDa
50 kDa
β-Actin
37 kDa
CDK9
25 kDa
20 kDa
TRMT6
75 kDa
CDK9 55kDa
50 kDa
CDK9 42kDa
37 kDa
150 kDa
100 kDa
p-STAT3
75 kDa
75 kDa
si-YTHDF1
si-YTHDF2
si-YTHDF3
si-YTHDC1
si-NC
Huh7
50 kDa
CDK9
37 kDa
50 kDa
β-Actin
37 kDa
STAT3
150 kDa
100 kDa
75 kDa
50 kDa
MCL1 43kDa
37 kDa
MCL1 34kDa
25 kDa
20 kDa
15 kDa
BCL-2
25 kDa
20 kDa
HepG2.2.15
150 kDa
si-YTHDF1
si-YTHDF2
si-YTHDF3
si-YTHDC1
100 kDa
si-NC
75 kDa
50 kDa
37 kDa
CDK9
25 kDa
150 kDa
100 kDa
β-Actin
75 kDa
50 kDa
37 kDa
50 kDa
37 kDa
β-Actin
25 kDa

## Slide 8
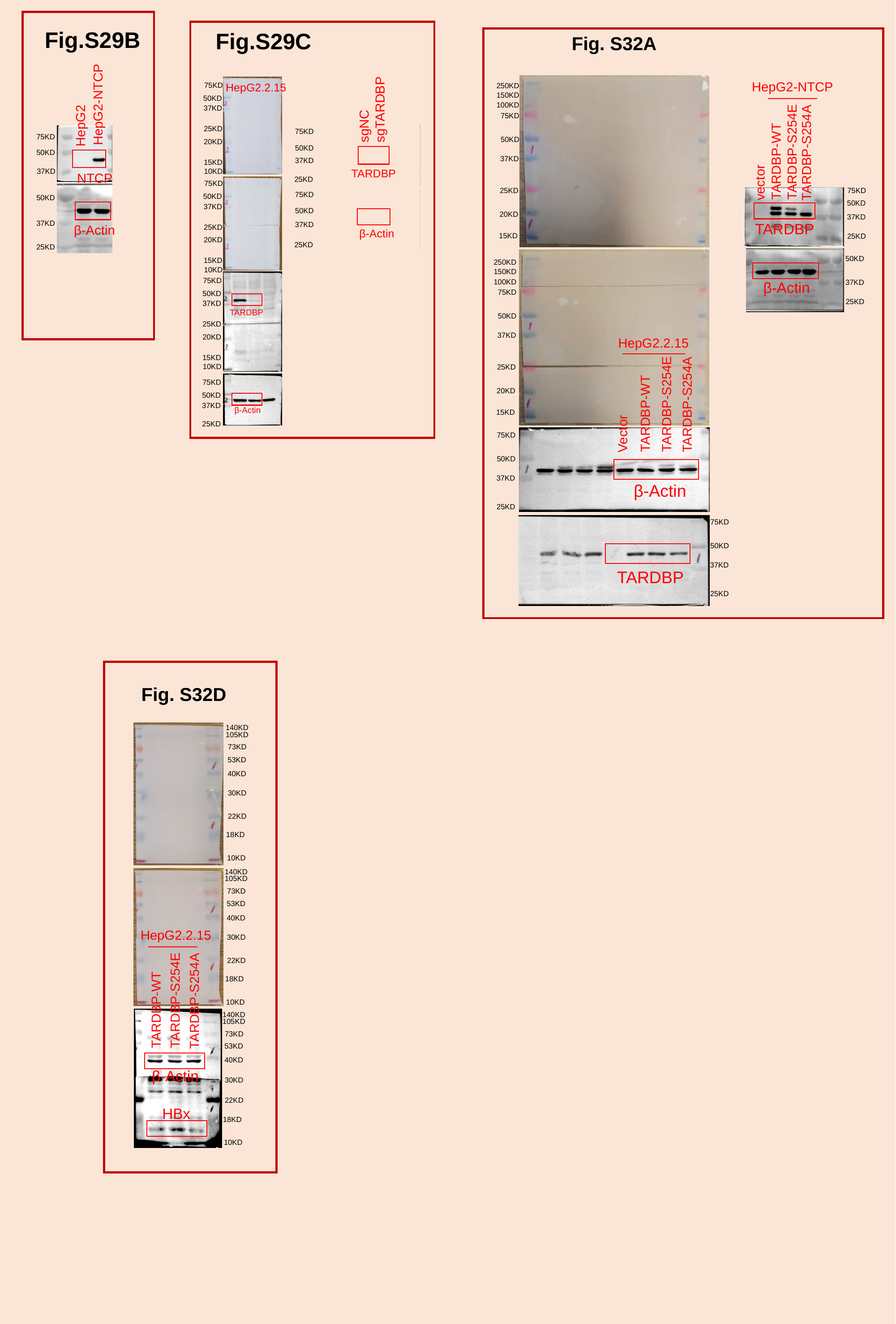

Fig.S29B
Fig.S29C
Fig. S32A
HepG2-NTCP
HepG2
75KD
50KD
37KD
NTCP
50KD
37KD
β-Actin
25KD
sgTARDBP
sgNC
75KD
50KD
37KD
TARDBP
25KD
75KD
50KD
37KD
β-Actin
25KD
250KD
150KD
100KD
75KD
50KD
37KD
25KD
20KD
15KD
250KD
150KD
100KD
75KD
50KD
37KD
HepG2.2.15
25KD
20KD
TARDBP-S254E
TARDBP-S254A
TARDBP-WT
15KD
Vector
75KD
50KD
37KD
β-Actin
25KD
75KD
50KD
37KD
TARDBP
25KD
HepG2-NTCP
HepG2.2.15
75KD
50KD
37KD
25KD
20KD
15KD
10KD
75KD
50KD
37KD
25KD
20KD
15KD
10KD
75KD
50KD
37KD
TARDBP
25KD
20KD
15KD
10KD
75KD
50KD
37KD
β-Actin
25KD
TARDBP-S254E
TARDBP-S254A
TARDBP-WT
vector
75KD
50KD
37KD
TARDBP
25KD
50KD
37KD
β-Actin
25KD
Fig. S32D
140KD
105KD
73KD
53KD
40KD
30KD
22KD
18KD
10KD
140KD
105KD
73KD
53KD
40KD
HepG2.2.15
30KD
22KD
18KD
TARDBP-S254E
TARDBP-S254A
10KD
TARDBP-WT
140KD
105KD
73KD
53KD
40KD
β-Actin
30KD
22KD
HBx
18KD
10KD
